# Evolutionary origin and genomic organisation of runt-domain containing genes in arthropods

**DOI:** 10.1186/1471-2164-9-558

**Published:** 2008-11-25

**Authors:** Elizabeth J Duncan, Megan J Wilson, James M Smith, Peter K Dearden

**Affiliations:** 1 Laboratory for Evolution and Development, Department of Biochemistry, University of Otago, Aotearoa-New Zealand; 2 National Research Centre for Growth and Development, Department of Biochemistry, University of Otago, PO Box 56, Dunedin, Aotearoa-New Zealand

## Abstract

**Background:**

Gene clusters, such as the *Hox *gene cluster, are known to have critical roles in development. In eukaryotes gene clusters arise primarily by tandem gene duplication and divergence. Genes within a cluster are often co-regulated, providing selective pressure to maintain the genome organisation, and this co-regulation can result in temporal or spatial co-linearity of gene expression. It has been previously noted that in *Drosophila melanogaster*, three of the four runt-domain (RD) containing genes are found in a relatively tight cluster on chromosome 1, raising the possibility of a putative functional RD gene cluster in *D. melanogaster*.

**Results:**

To investigate the possibility of such a gene cluster, orthologues of the *Drosophila melanogaste*r RD genes were identified in several endopterygotan insects, two exopterygotan insects and two non-insect arthropods. In all insect species four RD genes were identified and orthology was assigned to the *Drosophila *sequences by phylogenetic analyses. Although four RD genes were found in the crustacean *D. pulex*, orthology could not be assigned to the insect sequences, indicating independent gene duplications from a single ancestor following the split of the hexapod lineage from the crustacean lineage.

In insects, two chromosomal arrangements of these genes was observed; the first a semi-dispersed cluster, such as in *Drosophila*, where *lozenge *is separated from the core cluster of three RD genes often by megabases of DNA. The second arrangement was a tight cluster of the four RD genes, such as in *Apis mellifera*.

This genomic organisation, particularly of the three core RD genes, raises the possibility of shared regulatory elements. *In situ *hybridisation of embryonic expression of the four RD genes in *Drosophila melanogaster *and the honeybee *A. mellifera *shows no evidence for either spatial or temporal co-linearity of expression during embryogenesis.

**Conclusion:**

All fully sequenced insect genomes contain four RD genes and orthology can be assigned to these genes based on similarity to the *D. melanogaster *protein sequences. Examination of the genomic organisation of these genes provides evidence for a functional RD gene cluster. RD genes from non-insect arthropods are also clustered, however the lack of orthology between these and insect RD genes suggests this cluster is likely to have resulted from a duplication event independent from that which created the insect RD gene cluster. Analysis of embryonic RD gene expression in two endopterygotan insects, *A. mellifera *and *D. melanogaster*, did not show evidence for coordinated gene expression, therefore while the functional significance of this gene cluster remains unknown its maintenance during insect evolution implies some functional significance to the cluster.

## Background

Runt-domain (RD) containing proteins are transcriptional regulators that are able to activate or repress transcription dependent on the biological context of the cell [1] and are involved in cell fate specification and cell differentiation during development in metazoans [2]. The RD, a DNA binding domain, consists of a 128 amino acid motif that mediates DNA binding, heterodimerisation with a β-subunit (similar to the mammalian CBFβ proteins) [3] and also nucleotide binding [4] Outside of the RD itself, the only conserved motif in this protein family is a C-terminal pentapeptide (VWRPY, or I/LWRPF) which is thought to mediate the interaction with the transcriptional co-repressor Groucho [5].

The number of RD genes reported in metazoan genomes varies, and it is generally acknowledged that the RD genes have been independently duplicated in the chordate and insect lineages [6,7]. Studies of sea urchin (*Strongylocentrotus purpuratus*) runt and the mammalian runt orthologues Runx1, Runx2 and Runx3 have shown that RD proteins play an essential role in actively cycling cells and regulate cell proliferation prior to terminal differentiation [8]. All three RD proteins in humans are associated with disease, particularly cancer. Runx1, for example, is required for haematopoiesis and mutations in this gene cause leukaemia. Runx2 is required for bone development, and Runx3 for growth and differentiation of the gastric epithelium [8]. The *Caenorhabditis elegans *and *S. purpuratus runt *genes are also expressed in the gut [9,10], implying that runx3 may be the ancestral *runx *gene in chordates [2], although this is not supported by molecular data [6].

The number of RD genes found in ecdysozoan genomes also varies, with a single RD gene in *C. elegans*, and three in the mosquito *Anopheles gambiae *[6]. In *Drosophila*, four RD genes are present in the genome: *runt *and *lozenge*, which have been very well characterised and two uncharacterised RD containing genes, *CG42267 *(previously *CG15455*) and *CG34145 *[6].

In *Drosophila*, *runt *is involved in many developmental processes, including embryonic segmentation [11,12], sex determination [13,14] and neurogenesis [15,16]. *Runt *is initially expressed in a broad domain in the centre of the syncitial blastoderm embryo regulated by *bicoid *[17,18] and *tailless *[17], and is required for activation of *sex-lethal*, the major switch in *Drosophila *sex-determination [13]. *Runt *is a pair-rule gene but also has gap gene like properties [19]. During segmentation *runt *is expressed in the blastoderm with a dual segment periodicity, becoming expressed in seven stripes of cells. The expression of each of the seven stripes appears to be regulated independently by the gap genes, but possibly also by the maternal coordinate gene *bicoid *[17]. Other pair-rule genes, particularly *hairy*, act to maintain the *runt *expression pattern [17,18]. Later in the segmentation cascade *runt *is expressed with segmental periodicity, resulting in the formation of the secondary stripe pattern. This pattern is dependent on all of the pair-rule genes except *sloppy-paired *[17]. Following segmentation *runt *is expressed in a subset of cells in the developing nervous system and determines cell fate in specific neural lineages [16].

The other well-characterised *Drosophila *RD protein, *lozenge*, also has roles in several developmental processes. It has been shown to function in the development of antennae and tarsal claws, as well as haematopoiesis and female fertility [20]. *Lozenge *was first identified on the basis of its role in eye development and is critical for the differentiation of both photoreceptor neurons and non-neuronal cone cells [21,22]

Gene clusters, such as the *Hox *gene complex, are often ancient and are found in animals separated by hundreds of millions of years of evolution. While gene clustering as a phenomenon appears more common in genomes of higher complexity, for instance in mammalian as compared to insect genomes, very ancient clusters such as those involving the *wnt *family of genes [23] and the *Fox *gene transcription factors [24] have been reported across evolution. In the well-studied cases of the *Hox*, *Wnt *and *Fox *clusters, genes within the cluster show coordinated expression during embryogenesis, implying the genes may be regulated by shared cis-regulatory elements, which invoke stabilising selection to maintain the gene cluster. For the majority of gene clusters, however, it is not clear whether the genomic organisation is maintained as a result of functional constraints, or whether it is merely by chance that no chromosomal rearrangements disrupting the complex have become fixed.

It has been previously noted that of the *Drosophila *RD genes, *runt*, *CG34145 *and *CG42267 *are closely linked on chromosome 1 [6], and are likely to have evolved as a result of tandem gene duplication. The close linkage of these genes may be due to the recent evolution of multiple RD genes in *Drosophila *or alternatively may indicate the presence of functional constraints that retain these genes in close proximity. It is possible to distinguish between these possibilities by comparison of the genomic organisation amongst species that are sufficiently diverged to have had numerous chromosomal rearrangements become fixed, resulting in a shuffling of both the order and spacing of genes.

A comprehensive survey of runt domain containing genes in a wide range of arthropods is now possible due to the availability of genome sequence for several key species. Here we identify the RD genes in the genomes of several endopterygotan insects, including the twelve *Drosophila *species [25], *Apis mellifera *(the honeybee) [26], *Nasonia vitripennis *(the jewel wasp) [27], *Tribolium castaneum *(the red flour beetle) [28]*Bombyx mori *(the silkmoth) [29] and *Aedes aegypti *(the yellow fever mosquito) [30]. The genomes of two fully sequenced exopterygotan insects, *Acyrthosiphon pisum *(the pea aphid) [31] and the *Pediculus humanus *(the human body louse) [32] were also included in this analysis. In addition two non-insect arthropod genome sequences are now available: the crustacean *Daphnia pulex *[33] and the chelicerate *Ixodes scapularis *[34]. We examine the phylogenetic relationships and genomic organisations of the RD genes in these organisms, and present evidence for a putative conserved runt complex in insects. To determine if the close genomic proximity of these RD genes in insects relates to functional conservation of regulatory elements, the embryonic expression profiles of the four RD genes were examined in *D. melanogaster *and the hymenopteran insect *A. mellifera*.

## Results

### Identification of a runt gene complex in *Drosophila *species

Three of the four RD genes in *D. melanogaster *are clustered within 163 kb on chromosome 1 and are likely to have arisen by tandem gene duplication [6]. There is very tight linkage between *CG42267 *and *CG34145*, but there are three annotated functionally unrelated genes that lie between *CG34145 *and *runt*. *Lozenge *is separated from the other three genes by a distance of over 11 Mb.

To determine whether the genomic organisation of this 'runt complex' is the result of functional constraint or has been maintained purely by chance, we utilised the recent release of the twelve *Drosophila *species genome sequences [25]. These species are approximately 40 – 60 million years diverged [35], and extensive gene shuffling within the chromosome arms has occurred even between moderately diverged genomes such as *D. melanogaster *and *D. yakuba *[25,36].

**Figure 1:**
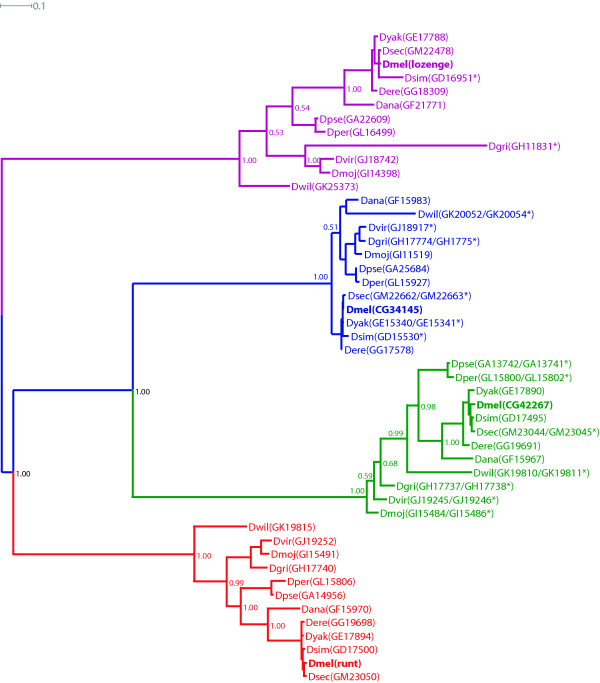
**Phylogeny and genomic organisation of *Drosophila *runt domain protein sequences**. An unrooted Bayesian phylogeny of *Drosophila* full-length RD protein sequences.  Phylogeny was constructed using MrBayes [67] under the Jones amino acid substitution model.   Posterior probabilities are shown on internal branches.  The RD proteins are subdivided into the four established orthologue groups.  Species names are abbreviated as follows: Dmel = *D. melanogaster*, Dsim = *D. simulans*, Dsec = *D. sechellia*, Dyak = *D. yakuba*, Dere = *D. erecta*, Dana = *D. ananassae*, Dpse = *D. pseudoobscura*, Dper = *D. persimilis*, Dwil = *D. wilistoni*, Dmoj = *D. mojavensis*, Dvir = *D. virilis*, Dgri = *D. grimshawi* and species names are followed by the FlyBase accession numbers.  Asterisks indicate protein sequences that have been manually annotated.  Orthologue groups are colour coded, the red group shows homology to the *Drosophila* runt protein, the purple to the *Drosophila* lozenge protein, the green to the *Drosophila* CG42267 protein and the blue to the *Drosophila* CG34145 protein.

Four RD genes were identified in each of these *Drosophila *genomes; in some cases the gene prediction was incomplete and complete predictions were obtained using Augustus [37]. In all cases the presence of both the RD and C-terminal VWRPY motif was confirmed. Identity was then assigned to these orthologues based on homology to the *D. melanogaster *protein sequences, as inferred by Bayesian phylogeny (Fig. 1) [see Additional file 1 for multiple sequence alignment].

The orthologues of *CG34145*, *CG42267 *and *Runt *are all found tightly linked (within 140 – 225 kb) in all 12 *Drosophila *species. In contrast, the orthologue of *lozenge *is usually present on the same chromosome but not closely linked to the other three RD genes. In species where scaffolds have been assigned to chromosome groups, this complex falls on the X chromosome (chromosome 1 in *D. melanogaster*).

**Figure 2:**
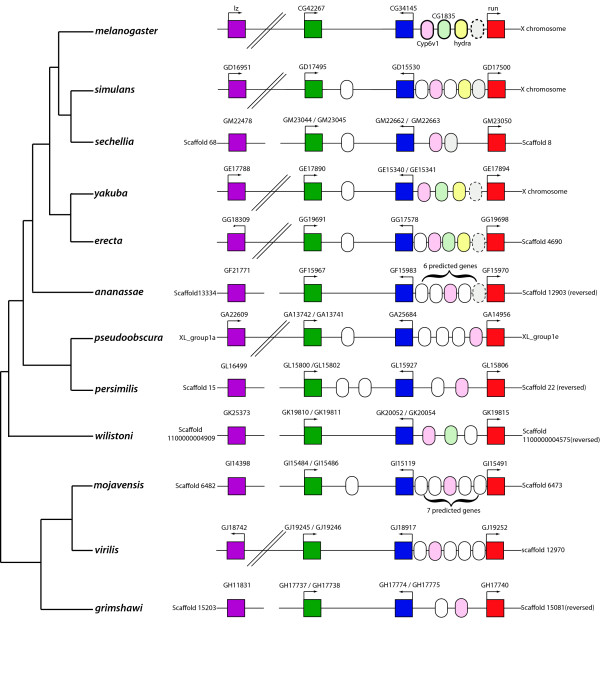
**Genomic organisation of the *Drosophila* RD gene clusters**. Orthologous genes are colour coded consistent with the phylogeny in Fig. 1.  In *D. melanogaster Cyp6v1, CG1835 *and* hydra* are annotated to fall between *CG34145* and *runt, Drosophila* orthologs of these genes are coloured pale pink, pale green and pale yellow, respectively.  A fourth gene, which is unannotated in *D. melanogaster* (as denoted by the dashed lines) is shown in light grey.

The number and identity of intervening genes is not well conserved (Fig. 2), although *Cyp6v1 *is always found between *CG34145 *and *runt*. *Hydra and CG1835 *are found between these two genes in the melanogaster sub-group, with the exception of *D. sechellia *where both genes are localised to an orphan scaffold (scaffold 600) which is likely to be an assembly error. *CG1835 *is also found in a conserved position in *D. willistoni*. There is also an unannotated gene likely to fall within this region in *D. melanogaster *that shows homology to *D. simulans GD17499*, this gene is found in all members of the melanogaster group but is unannotated in most (Fig. 2). It is possible that this sequence does not produce an mRNA transcript, although there is EST evidence in *D. melanogaster *to suggest that it does (BK003230). Despite the variation in number of intervening genes, the approximate spacing between the three genes is well conserved; with ~100 kb between *runt *and *CG34145 *(in all species except *D. sechellia*, where the distance is 20 kb) and ~168 kb between *CG23415 *and *CG42267*. The orientation of transcription is also conserved; orthologues of *CG42267 *and *runt *are transcribed from the sense strand, while *CG34145 *is transcribed from the anti-sense strand. The transcriptional orientation of *lozenge *is not well conserved, but it is transcribed from the sense strand in the majority of species, except *D. erecta *and *D. virilis*, possibly resulting from independent genome inversion events in these species.

Together, the phylogenetic and genomic organisation supports the duplication of the RD genes prior to the radiation of the *Drosophila *lineage, and the resultant cluster of RD genes shows remarkable stability since the divergence of *Drosophila *species 60 million years ago [35].

### Identification of a runt gene complex in other insect species

**Figure 3:**
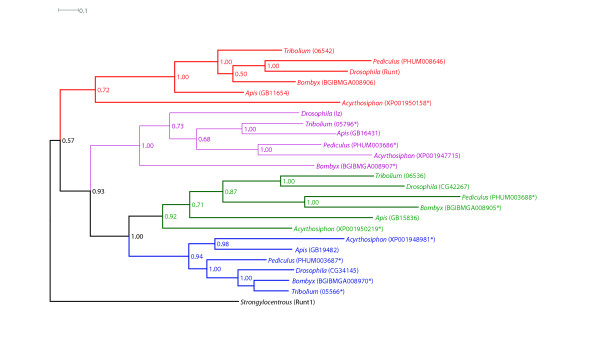
**Phylogeny of the runt domain protein sequences in endopterygotan and exopterygotan insects. **Rooted Bayesian phylogeny of full-length runt protein sequences.  Phylogeny was constructed in MrBayes; posterior probabilities are shown on internal branches; the tree was rooted with runt from the sea urchin *S. purpuratus*.  Orthologue groups are colour coded, the red group shows homology to the *Drosophila* runt protein, the purple to the *Drosophila* lozenge protein, the green to the *Drosophila* CG42267 protein and the blue to the *Drosophila* CG34145 protein.  Asterisks indicate protein sequences that have been manually annotated.

A survey of other fully sequenced insect genomes [26-28,31,32,38], shows that aspects of this runt gene complex are conserved, not only in the endopterygotan insects *A. mellifera*, *A. aegypti, B. mori *and *T. castaneum*, but also in the exopterygotan insects *P. humanus *(a Phthiapteran) and *A. pisum *(a Hemipteran). In all of these insects four RD genes were identified, and phylogenetic analyses (Fig. 3) were used to assign orthology to these genes. Where RD genes were incompletely or incorrectly annotated, particularly in the genomes of *T. castaneum*, *A. aegypti, A. pisum *and *B. mori*, annotation was performed manually with the assistance of Augustus [37] and GenomeScan [39], in all cases the presence of both the RD and C-terminal VWRPY motif was confirmed (the C-terminal pentapeptide in *A. pisum *XP001950158 is modified to IWRPF, and followed by an additional 5 amino acids prior to the stop codon). Proteins that were manually annotated in this way are indicated with an asterisk on the phylogeny presented in Fig. 3 [see Additional file 2 for multiple sequence alignment and phylogeny for endopterygotan insects, and Additional file 3 for multiple sequence alignment for both endopterygotan and exopterygotan insects].

It has been generally asserted that the RD gene family has derived from serial duplication of a single ancestral gene [6]. The structure of the insect RD gene phylogeny presented in Fig. 3, indicates that runt is ancestral to the other three RD proteins, and has been duplicated twice; one copy became the *runt *gene, while the second was further duplicated to evolve the *CG34145 *and *CG42267 *genes.

**Figure 4:**
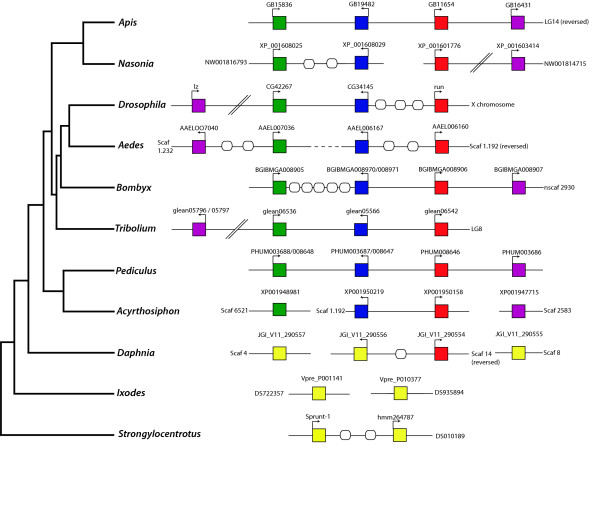
** Genomic organisation of the RD genes in insect and non-insect arthropod species**. Genomic organisation of the RD genes in insect and non-insect arthropod species.  Orthologous genes are colour coded, the red group shows homology to the *Drosophila* runt protein, the purple to the *Drosophila* lozenge protein, the green to the *Drosophila* CG42267 protein and the blue to the *Drosophila* CG34145 protein.

Comparison of the genomic organisation of these RD genes (Fig. 4) confirms a tight cluster of all four RD genes in *B. mori *and the most basally branching endopterygotan insect, *A. mellifera *[40]. In *A. mellifera *there are no intervening genes and the total cluster spans only 158 kb, however the *B. mori *cluster spans 330 kb and there are 5 annotated genes falling between *BmCG42267 (BGIBMGA008905) and BmCG34145 (BGIBMGA008970/008971)*. Surprisingly, a very tight cluster of all four RD genes can also be found in the exopterygotan insect *P. humanus*, where all four genes are found within 70 kb, with no intervening genes.

Data for *A. aegypti *and *A. pisum *were inconclusive due to gaps in the genome assemblies, but the orthologues of both *runt *and *CG34145 *were found on the same contig in close proximity, consistent with that seen in the other insects. In all cases the transcriptional orientation of these three core RD genes is completely conserved.

Interestingly, in the species where the four RD genes are tightly linked (*A. mellifera*, *B. mori *and *P. humanus*), the orthologue of *lozenge *lies proximal to the orthologue of *runt*. This contrasts to the remainder of species where *lozenge *often lies distal to the RD cluster, separated by a megabase or more.

### Expression of the RD genes: comparison of *Drosophila *and *A. mellifera*

The persistence of an intact RD gene cluster in all insects examined, despite more than 400 million years of evolution, and significant changes in life history and developmental modes, implies some functional relevance to the organisation of these genes. Analysis of *g*ene expression patterns has previously provided important insight into the nature of selective forces acting on gene clusters [41]. The expression patterns of *runt *and *lozenge *are well-understood in *D. melanogaster*, but the expression patterns of the other two RD genes remains uncharacterised. As the *Drosophila *RD complex shows evidence of dispersal, of *lozenge *in particular, we also chose to examine the expression patterns of all four RD genes in the basally branching endopterygotan insect, *A. mellifera*, which possesses a tight cluster of all four RD genes (Fig. 4).

**Figure 5:**
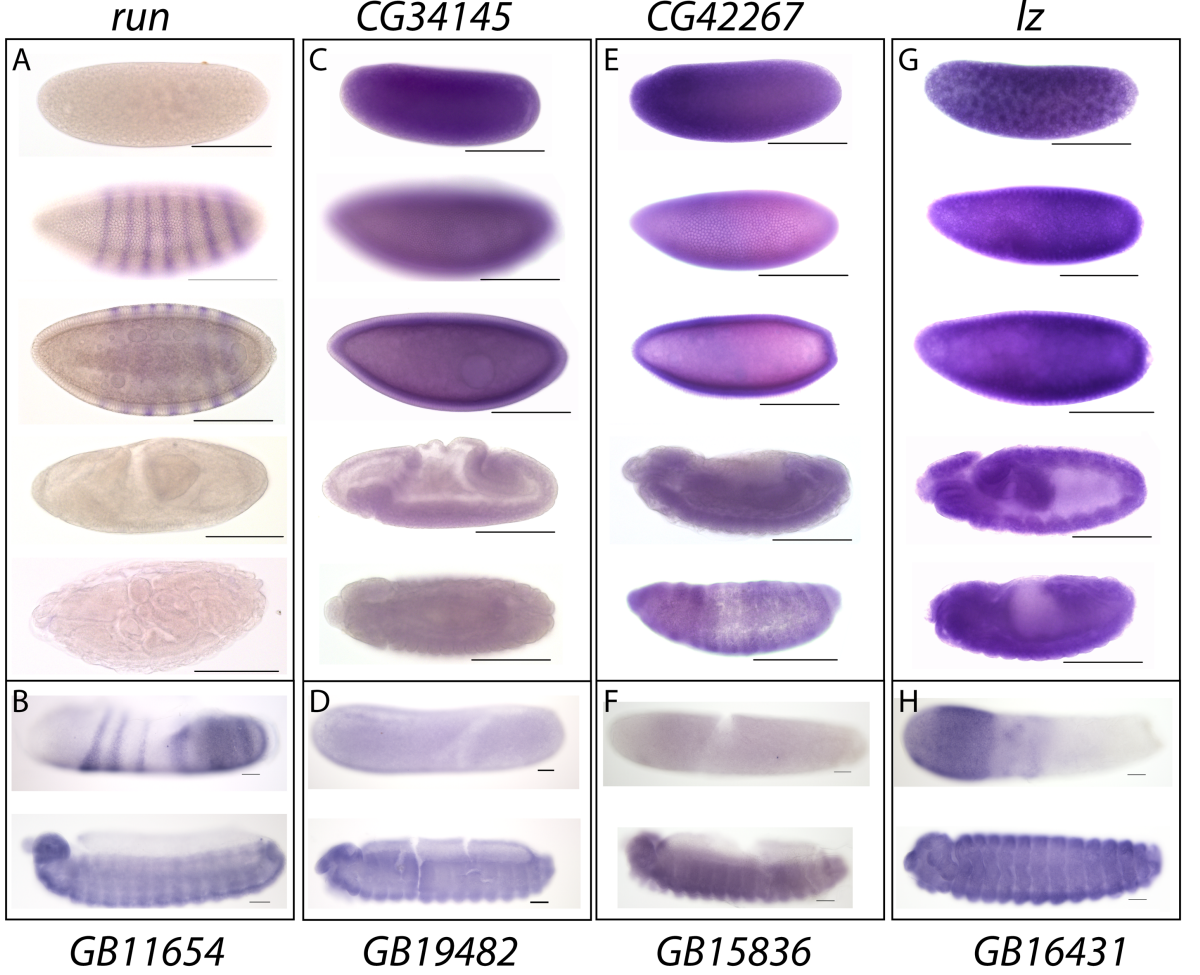
**Expression of the RD mRNAs in *Drosophila* and *A. mellifera* embryos. **Expression of RD gene RNA in *Drosophila* and Honeybee embryos using *in-situ* hybridisation. Genes arranged by orthology based on Fig. 3. A,C,E, and G Drosophila, scale bars =100 µm B, D, F and H honeybee, scale bars= 50 µm A) *run* (stage 2, 5 (two focal planes), 9 and 16) is in seven stripes in blastoderm embryos, no maternal expression is seen and expression is low in later development. B) *GB11654* (stages 5 and 9) is expressed in stripes in the blastoderm, and is expressed widely in later embryos. C) *CG34145* (stages 2, 5 (two focal planes), 7 and 15) is expressed maternally and then ubiquitously until stage 14. D) *GB19482* (stage 5 and 9) is expressed weakly in stage 5 embryos but ubiquitous later. E) *CG42267* (stage 2, 5 (two focal planes), 13 and 16) is expressed maternally and then ubiquitously. F) *GB15836* (stages 5 and 9) is expressed ubiquitously after blastoderm stages. G) *lz* (stages 3, 5 (two focal planes),11 and 15) is maternally provided localised to nuclei and ubiquitous by stage 5. H) *GB16431* (stages 5 and 9) is expressed in the anterior of blastoderm embryos and ubiquitously by stage 9.

*In situ *hybridisation of *Drosophila *and *A. mellifera *embryos was used to compare expression domains of the four RD mRNAs. *Drosophila runt *(Fig. 5a) is expressed early in development in a broad domain, then in a dual-segment periodicity in embryonic stages 4–6, and later in a segmental periodicity following gastrulation, as previously reported [42]. *A. mellifera runt *is expressed in a similar pattern (Fig. 5b), in stripes across the embryo that form in an anterior to posterior succession in a pattern that is typical of *A. mellifera *pair-rule genes [43].

The expression of *Drosophila lozenge *(Fig. 5g) is not consistent with that previously reported; a previous study demonstrated that by embryonic stage 7 *lozenge *expression is restricted to the anterior of the embryo in a sub-population of prohaemocytes, which terminally differentiate to form crystal cells (which are involved in innate immunity and wound healing) by embryonic stage 10 [44]. In this study *lozenge *mRNA is initially maternally provided and is localised to nuclei at early stages, expression later in development is ubiquitous. This difference in expression patterns is probably because the previous study used an enhancer fragment linked to a reporter gene to determine the expression pattern. *A. mellifera lozenge *(Fig. 5h) expression is detected very early (stage 1) in the anterior region of the embryo and is then detected in a distinct cell type, that develop from head mesoderm (Fig. 5h). These may be differentiating crystal cells, which develop from the head mesoderm in *Drosophila *[45]. By stage 9 (just prior to hatching), *Amlz *is expression is detected throughout the embryo (data not shown).

The embryonic expression of the other two RD genes in *Drosophila*, *CG34145 *(Fig. 5c) and *CG42267 *(Fig. 5e), have not been previously described. At all embryonic stages expression of both of these genes is ubiquitous, with *in situ *hybridisation of imaginal discs also showing no evidence of localised expression (data not shown). Both *Am*GB11654 and *Am*GB19482, the *A. mellifera *orthologues of these genes, are also expressed ubiquitously throughout late stages of embryonic development (Fig. 5d and 5f).

While clustered genes often show coordinated expression, neither the *Drosophila *nor *A. mellifera *RD genes (Fig. 5) demonstrate strong coordination of either temporal or spatial expression during embryonic development.

### The evolution of RD proteins in metazoa and arthropods

The presence of a runt domain cluster (Fig. 4) in all insect species examined, together with the phylogenetic data presented in Fig. 3, suggests that the duplication of the RD genes occurred prior to the divergence of the insect lineage into these two branches. In order to examine the evolution of these RD genes, and this cluster in a wider context, the genome sequence of two non-insect arthropods were utilised. Examination of the genome sequences of the crustacean *D. pulex *and the chelicerate *I. scapularis *clearly demonstrates multiple RD genes in both of these species, contradicting previous assertions that non-insect arthropods have only a single RD gene [7]. The water flea *D. pulex *has four RD genes, and two of these fall on the same scaffold (Fig. 4). In contrast *I. scapularis *only has two RD genes, and at present these fall on two independent scaffolds. Extensive searches of the *I. scapularis *genome have been unable to identify any further orthologues, however this could be due to the preliminary nature of the genome assembly. Two RD genes have also been identified in the spider *Cupiennius salei *[46], and one in the two-spotted spider mite, *Tetranychus urticae *[47].

**Figure 6:**
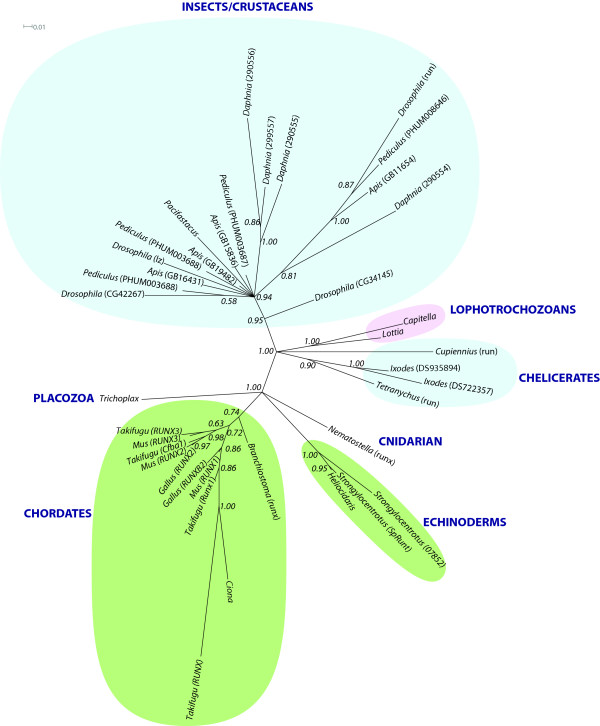
**Phylogenetics of the runt-domain protein families in Metazoan species. **Phylogeny of metazoan Runt/runx proteins based on alignment of 125 amino acids of their RD sequences. Unrooted phylogram constructed using MrBayes [67] under the Jones amino acid substitution model.   Deuterostome species are highlighted in green, arthropod species in blue and lophotrochozoans in pink.   Posterior probabilities are shown on internal branches.

This presence of multiple RD genes in the genomes of non-insect arthropods raises the possibility that the duplication of the RD genes occurred prior to the radiation of arthropod species and that multiple RD genes and their genomic organisation were present in the last common ancestor of arthropods. To examine this possibility phylogenetic analyses were carried out using the RD domain sequence from the proteins of known RD genes. Representatives from several non-arthropod metazoan clades were selected, including the basally branching non-bilaterian *Trichoplax adhaerens *[48], the cnidarian *Nematostella *[49], and the RD genes from two sequenced lophotrochozoan species, the gastropod snail *Lottia gigantea*, and the polychaete *Capitella *(Fig. 6) [see Additional file 4 for multiple sequence alignment].

Phylogenetic analysis indicates that the duplication of the RD genes has occurred independently in the deuterostome and arthropod lineages, and that the duplication events that gave rise to the multiple vertebrate *runx *genes, have occurred independently of the duplications giving rise to the multiple RD genes in the sea urchin, *S. purpuratus*. Somewhat surprisingly however, the phylogeny also indicates independent duplication events have given rise to the chelicerate RD genes and the crustacean/insect RD genes. The phylogeny indicates a single origin for chelicerate RD genes independent of the duplications that gave rise to the RD genes in *Daphnia *and in insects. Supporting this is the expression pattern of the RD gene cloned from *Tetranychus urticae *(the two-spotted spider mite), which clearly suggests a role for *Tu-run *in segmentation, but also in limb specification [47], a role not seen for either the *A. mellifera *or *Drosophila *orthologues of *runt*, but consistent with expression of *Cs*-*runt *in *C. salei*.

Interestingly, a single *D. pulex *gene (*Dp290554*) groups with the clade of insect RD proteins, while the other three *D. pulex *genes form an independent clade. This seems to indicate that runt is the ancestral protein in crustaceans and chelicerates. However, the crustacean/insect clade on the whole is not well resolved, as by necessity, this phylogeny only included 125 amino acids of the RD as two of the *D. pulex *sequences are incorrectly annotated and are missing 10 amino acids of the RD, thus reducing the informational content of the alignment.

**Figure 7:**
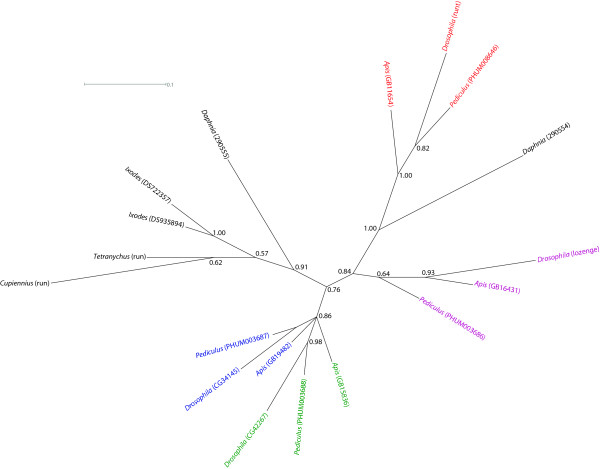
**Phylogenetics of the runt-domain protein families in Arthropods. **Phylogeny of arthropod RD proteins based on alignment of the full-length RD sequences (133 amino acids). Unrooted phylogram drawn from Bayesian phylogenetic analysis of RD domain sequences under the Jones amino acid substitution model.  The insect proteins are colour coded based on homology to the *Drosophila* sequences as in Figs. 1 and 3. Posterior probabilities are shown on internal branches.

Bayesian phylogeny of the full RD from arthropod species, minus the truncated *D. pulex *sequences (Fig. 7) [see Additional file 5 for multiple sequence alignment], supports the independent duplication of RD genes in chelicerates and in crustaceans and insects. As before Dp290554 groups reliably with the insect runt RD sequence, providing evidence for runt being the ancestral RD protein in these species, which was suggested (albeit weakly) by the phylogeny of endopterygotan and exopterygotan full-length protein sequences in Fig. 3.

## Discussion

In this study we identified four RD genes in the twelve sequenced *Drosophila *genomes [25] and in several non-dipteran endopterygotan insects including *A. mellifera*, *A. aegypti*, *B. mori*, and *T. castaneum*. Four RD genes were also identified in the exopterygotan insects, *P. humanus *and *A. pisum*, and the crustacean *D. pulex*. Two different arrangements of RD genes were observed in these species: a semi-dispersed cluster was seen in *Drosophila *species and in *T. castaneum*; and an intact, tight cluster with few or no intervening genes was seen in *A. mellifera*, *B. mori *and *P. humanus*. In the other species examined, the genome assembly was fragmented such that one or more RD genes was localised to a different contig and thus no assertions about cluster organisation could be made. In species with an intact RD gene cluster, *lozenge *was found to be located proximal to *runt*, while in the species with a semi-dispersed cluster *lozenge *was often localised to the same chromosome, but separated from the core cluster by megabases of DNA. The genomic organisation of the remaining three RD genes, including gene spacing and transcriptional orientation, was remarkably conserved.

The number and identity of intervening genes in this cluster varies; between two and seven genes lie between the *CG34145 *and *runt *orthologues in *Drosophila *species, including *Cyp6v1*. *Cyp6v1 *is a P450 enzyme, and is part of a large gene family that is prone to duplication [50]. *Hydra *and *CG1835 *are localised between *CG34145 *and *runt *in *D. melanogaster*, *D simulans*, *D. yakuba *and *D. erecta*, these two genes are localised to an orphan scaffold in *D. sechellia *(scaffold 600), but this is likely a problem with the genome assembly. *Hydra *is found exclusively in the melanogaster sub-group, with no orthologues found in any other dipterans or insects (data not shown), suggesting recent evolution of this gene. Conversely *CG1835 *is also found in *D. willistoni *suggesting at least two independent losses of this gene (in the obscura group and *D. ananassae*) A fourth gene is found in this region in the melanogaster species group, however this gene is unannotated in *D. melanogaster *and therefore may represent a conserved regulatory sequence rather than a protein coding gene. *CG42267 *and *CG34145 *are in much tighter linkage, and the majority of *Drosophila *species have either one or no intervening genes in this region, other than *D. persimilis *which has three. Based on established taxonomic relationships between *Drosophila *species it is likely that there has been multiple gains or losses of genes in these genomic regions, although this effect may be exaggerated due to inaccuracies in the genome assemblies or gene prediction models. In all but one non-dipteran insect species, no intervening genes were identified in the RD gene cluster, the only exception being in *B. mori *where five annotated genes fall between *BmCG42267 *and *BmCG34145*. The presence of intervening genes in the Dipteran RD cluster, seemingly unrelated in either expression or function, is consistent with the RD complex being considered a genomic regulatory block [51] and is reminiscent of the Hox complex in *Drosophila*, where invasion of cuticle genes into the ANT-C complex between *labial *and *proboscipedia *has been attributed to the accelerated rate of evolution in this lineage, and may have contributed to splitting of the Hox complex in *Drosophila *[52].

The presence of an intact cluster in the endopterygotan insects *A. mellifera *and *B. mori*, and the exopterygotan insect *P. humanus*, strongly supports the phylogenetic data that duplication of the RD genes occurred prior to the radiation of insect species, and implies that the ancestral insect genome had a very tight cluster of four RD genes. The semi-dispersed gene cluster seen in *Drosophila *and *T. castaneum *may indicate that the functional constraint holding *lozenge *in the gene cluster has been lost or modified in the Coleopteran and Dipteran lineages. The pattern of RD gene dispersal in these species is similar to that seen for the Fox family of transcription factors, where relaxation of selection on the cluster has resulted in cluster fragmentation [24]. The dissolution of such clusters also may be the result of accelerated genome evolution associated with *Drosophila *lineages [25]. However, the *T. castaneum *genome is not noted to have undergone particularly rapid evolution [53] and consistent with this, the *T. castaneum *genome has just a single Hox complex, with no evidence of dispersal [53]. A recent study has shown that while up to 91% of orthologues remain in synteny between *D. melanogaster *and the most diverged *Drosophila *species, *D. virilis *synteny is only 3% between *D. melanogaster *and *B. mori*, and 10% between *D. melanogaster *and *A. mellifera *[54]. This, along with the high observed rate of turnover of intervening genes within the RD cluster, would indicate that the conservation of genomic organisation seen for RD genes across insect genomes would be very unlikely to have occurred simply by chance, and is likely to have been maintained by selection, presumably in favour of retaining function.

Functionally related genes are known to have, in some cases, conserved genomic organisation [55]. In general, genes that have evolved as a result of tandem gene duplications tend to maintain a similar function to their parental copy due to sharing of the same regulatory elements [56], and this sharing of regulatory elements can facilitate the evolution of co-regulation [41]. To examine the possibility of co-regulation acting as a selective force to maintain the RD gene cluster, the expression of the four RD genes was examined in *D. melanogaster *(which houses a semi-dispersed RD cluster) and in *A. mellifera *(which houses an intact 'ancestral' RD cluster) as a means of investigating functional conservation of RD gene orthologues between these species. *In situ *hybridisation did not reveal any strong evidence for spatial or temporal co-regulation of RD gene orthologues during *Drosophila *or *A. mellifera *embryogenesis. Although it is possible that localization or expression of the protein (which would not be detected by *in situ *hybridisation) is altered independently of that of its mRNA. Three of the four RD genes are expressed ubiquitously in the blastoderm stage of embryogenesis in *Drosophila*, while the fourth, *runt *is expressed in a pair-rule pattern in seven stripes. Therefore the cluster of RD genes in insects could be driving co-regulation of expression at the blastoderm stage, while *runt *expression is negatively regulated by fushi tarazu [18], however this is not the case for *A. mellifera*. It is possible that overlapping rather than shared regulatory elements are responsible for the retention of the gene cluster. However, this scenario would also appear unlikely; while the *runt *enhancer in *Drosophila *covers a distance of approximately 14 kb [57], the intergenic distance between *CG34145 *and *runt *averages around 100 kb across *Drosophila *species, and the number of intervening genes in this region varies markedly, highlighting that this genomic region is free to evolve.

There has been much speculation about the origin of the RD proteins, and it is largely accepted that the RD genes have been independently duplicated in the deuterostome and protostome lineages [6,7]. However, the question of RD gene evolution in arthropods has not been addressed specifically. Phylogenetic analyses presented here support the notion of gene duplication prior to the radiation of insect species, as all insect RD proteins could be placed into four orthologue groups. However, two non-insect arthropods whose genomes have been sequenced completely, the chelicerate *I. scapularis *and the crustacean *D. pulex*, also have multiple RD genes, and phylogenetic analyses support the notion that these genes have been independently duplicated in insect and non-insect arthropod lineages. This phylogeny implies that the ancestor of arthropods had a single RD gene. However, gene conversion events are known to result in phylogenetic analyses that overestimate the incidence of gene duplication. This is an issue for linked genes, such as those generated by tandem gene duplication [58], and has resulted in spurious phylogenies for insect gene families [59].

The phylogeny presented here indicates that there have been multiple duplications of RD genes across bilaterian evolution, and as there is no evidence for the duplicated copies decaying into pseudogenes, these duplicated copies are likely to have undergone rapid divergence resulting in subfunctionalisation or neofunctionalisation of the protein functions. This kind of rapid divergence could be due to the chromosomal location of the ancestral gene, as it is known that areas of the genome are more prone to this phenomenon, such as the X chromosome in *Drosophila*, where the RD gene complex is localised [60]. Consistent with the hypothesis of rapid divergence following duplication, there is clear evidence for neofunctionalisation of the duplicated genes, in both ecdysozoan and deuterostome species. For instance, the *C. elegans *RD gene and one of the sea urchin RD genes, which are most closely related to mammalian *runx3*, are both expressed in the developing gut, a role not seen in the cnidarian *Nematostella*. This has led to the assertion that *runx3 *may be the ancestral RD gene in chordates [2], although this hypothesis is yet to be supported by molecular data [6].

To date there has been no discussion about the evolution of RD genes in arthropods, and the phylogenetic analyses presented here indicates that the last common ancestor of all arthropods had a single RD gene, which has been independently duplicated in the chelicerate and crustacean/insect lineages. All arthropod RD genes possess a runt DNA binding domain and a Groucho binding domain, suggesting that these proteins are able to act as transcriptional regulators, and both runt and lozenge have been shown to modulate developmental gene expression in *Drosophila *[20]. The functions of CG34145 and CG42267 are unknown, although CG42267 was identified as having a role in the control of cell survival in an RNAi screen [61] and it has been suggested that cross-talk between RD proteins and the cell cycle may modulate cell cycle progression [62]; this would also be consistent with the ubiquitous expression of *CG42267 *and *CG34145 *during embryogenesis in *Drosophila *and *A. mellifera *[62]. Interestingly, one of the two sea urchin RD genes is also highly expressed in proliferating cells during embryogenesis [10], perhaps supporting an ancestral role for the RD genes in cell-cycle modulation.

The phylogenetic data indicates that runt is the ancestral RD gene in insects and crustaceans. In insects runt has a key role in segmentation [42], and orthologs of runt have been shown to be involved in the segmentation of two chelicerates [46,47], raising the possibility that one of the functions of the single RD gene in the last common ancestor of all arthropods was a role in segmentation, and that the duplication and diversification of the RD genes has served to recruit the transcriptional co-repressor Groucho into new developmental niches, such as eye development [21].

## Conclusion

RD genes are present in the genomes of organisms throughout metazoa, often found in multiple copies. Phylogenetic evidence supports the notion that these duplication events leading to multiple genomic copies of RD genes have occurred independently in the deuterostome and ecdysozoan lineages. Within arthropods, RD genes are also clustered, however phylogenetic data supports the independent duplication of RD genes in the chelicerate and crustacean/insect lineages. The RD genes in insects bear all the hallmarks of a functional gene cluster, in particular the very tight association of three of the four RD genes; CG42267, CG34145 and runt. However, *in situ *hybridisation of RNA expression did not suggest any temporal or spatial co-regulation of these RD genes in either *Drosophila*, which has a semi-dispersed cluster formation or in *A. mellifera*, which has an intact complex. It is possible that the retention of such a complex is not associated with functional constraints but is merely an artefact of tandem gene duplication; however, given the accelerated genome evolution observed in both *Drosophila *and mosquito species [63], but not *T. castaneum *[53], this would seem unlikely. While the significance of this RD gene cluster in insect species remains unknown the persistence of this cluster across all insect species implies functional importance.

## Methods

### Beekeeping

*Apis mellifera *were cultured using standard techniques in Dunedin, New Zealand. *A. mellifera *embryos were collected from frames removed from nucleus boxes containing small *A. mellifera *colonies.

### Phylogenetic analyses and cluster identification

Full-length RD metazoan protein sequences were obtained from relevant genome databases [25-28,30-34], or from NCBI. Unannotated RD proteins were predicted with the assistance of Augustus [37], and GeneScan [39]. Where required, runt domains were extracted from full length protein sequences using the hmmer suite of programs [64], against the Pfam runt hmm model (PF00853, 138 amino acids) [65].

Multiple alignments were carried out using ClustalX [66]. The multiple alignments were analysed using MrBAYES v3.1.2 [67] under the Jones model with default priors. The Jones model was chosen as the most appropriate model of amino acid substitution after preliminary analyses using MrBAYES with mixed models. The Monte Carlo Markov Chain search was run with four chains over 1000000 generations with trees sampled every 1000 generations. The first 250000 trees were discarded as 'burn-in'.

### Identification and cloning of RD genes

Drosophila RD genes were identified in FlyBase [68], and *A. mellifera *orthologues of these *Drosophila *genes were identified by tBlastN [69] searches of the *A. mellifera *genome [26].

RNA was extracted from *A. mellifera *embryos using the RNeasy Mini Kit (Qiagen). cDNA was generated using Superscript II reverse transcriptase (Invitrogen) and an oligo-dT primer. The cDNA was used as a template for amplification of putative RD genes using the following oligonucleotide primers:

CG34145R ATGTGATCCATGACGCTCTG

CG34145F ATGTGCACTCCAGCCAGAAT

CG15455F AACAGCAGCAGCAACATCAG

CG15455R ATGTGGAGATCCCGTCTTGA

GB19482l GAGCAATTCATGGGGATACG

GB19482r ACTGGTTCCCGTACAACTGG

GB16431l CAAAACGAGGCAGACTCACA

GB 16431r GTGTCCCGACGGAAGAACAGT

GB 15836f AAGCGGTAGAGGAAAGAGTT

GB 15836r GGTGAAGACCTTGAAAGTGA

GB 11654 ATGCACTTACCGGAGGGCCCACTA

GB 11654 CTCGTGCTCGAGTCGCCCTAGTAG

runl CCACGACGAGTGTGATTAC

runr GACGACGCGTCCAAATA

lz3 TGATTCTGATTGACCGTGGA

lz5 CATGGGCATGAATCACTACG

Amplified DNA fragments were purified using the High Pure PCR purification kit (Roche) and cloned into pCRII-TOPO (Invitrogen). The sequence and orientation of each cloned gene fragment was confirmed by DNA sequencing.

### *In situ *hybridisation

*A. mellifera *embryos were collected and fixed as described [70]. Antisense or sense digoxigenin (DIG) labelled RNA probes were produced by *in vitro *transcription from linearised DNA templates containing cDNA fragments. *In situ *hybridisation on *A. mellifera *embryos were performed as previously described [70].

## Authors' contributions

EJD and PKD performed the bioinformatics and phylogenetic analyses. MJW performed the *A. mellifera *ISH experiments. PKD performed the *Drosophila *ISH experiments and conceived the study. EJD, JMS, MJW and PKD wrote the manuscript.

## Supplementary Material

Additional file 1 **multiple sequence alignment of full-length *Drosophila *RD proteins**. ClustalX alignment of full-length RD protein sequences from 12 *Drosophila *species: *D. melanogaster*, *D. simulans*, *D. sechellia*, *D. yakuba*, *D. erecta*, *D. ananassae*, *D. pseudoobscura*, *D. persimilis*, *D. wilistoni*, *D. mojavensis*, *D. virilis*, and *D. grimshawi*. Click here for file

Additional file 2 **Phylogeny and multiple sequence alignment of endopterygotan insects full-length RD protein sequences**. ClustalX alignment and Bayesian phylogeny of full-length RD protein sequences from a six endopterygotan insects: *Drosophila melanogaster*, *Aedes. aegypti*, *Tribolium castaneum*, *Nasonia vitripennis*, *Apis mellifera *and *Bombyx mori*. Alignment includes the outgroup *Strongylocentrotus purpuratus*. Click here for file

Additional file 3 **Multiple sequence alignment of full-length RD protein sequences from exopterygotan and endopterygotan insects**. ClustalX alignment of full-length RD protein sequences from six insect species: *Drosophila melanogaster, Tribolium castaneum, Acyrthosiphon pisum*, *Pediculus humanus, Bombyx mori and Apis mellifera*. Alignment includes the outgroup *Strongylocentrotus purpuratus*. Click here for file

Additional file 4 **Multiple sequence alignment of the RD protein sequences from a number of metazoan species**. ClustalX alignment of RD protein sequences from a number of metazoan species including representative species from the insects, crustaceans, chelicerates, cnidarians, lophotrochozoans, echinoderms and chordates. Click here for file

Additional file 5 **Multiple sequence alignment of arthropod RD domain sequences**. ClustalX alignment of the RD from a number of arthropod species, including three insect species and four non-insect arthropods. Click here for file
