# Supplementary material for: Evolutionary origin and genomic organisation of runt-domain containing genes in arthropods
Source: BMC Genomics. 2008 Nov 25;9:558. doi: 10.1186/1471-2164-9-558 (PMC2631020; doi:10.1186/1471-2164-9-558)
Supplement: Additional file 1 — multiple sequence alignment of full-length Drosophila RD proteins. ClustalX alignment of full-length RD protein sequences from 12 Drosophila species: D. melanogaster, D. simulans, D. sechellia, D. yakuba, D. erecta, D. ananassae, D. pseudoobscura, D. persimilis, D. wilistoni, D. mojavensis, D. virilis, and D. grimshawi. [file 1471-2164-9-558-S1.pdf]

Additional File A1: multiple sequence alignment of full-length *Drosophila* RD proteins.

```
DmeRun           1 AAAAAAAAAAAAAVAQGPGPQQ-SSNAT------------TASAIAINPAQS-----------LANTSTHSASSTGSSTP
DvirGJ19252      1 QVLAAAAAAAAAAAAATNVSQ-GNISTH------------SASSTGSSTPDN-----------NSSSNNNNNSSNSS---
DanaGF15983      1 MHLTTTSSNSTASNANNNNNNNNNNNT-----------------------------TNNNNNS-----SSNNNNNSSQTN
DvirGJ18917*     1 MHLTTTSGNSTASNANSNNNNN----------------------------------SNNNNNN-----TANNNNSSSSNN
DseGM22662/63*   1 MHLTTTSSNSTASNANNNNNN-----------------------------------TANNNN------TSSNNNTTNTNG
DpseGA13742/42*  1 MHISAEVSSTTSNQIQQQQQQQQQQQQQQHQQL---------------HQQLHQQHQQQTTPTATTTTGGSTTKRRNAES
DyakGE17890      1 MHISAEVSSTTSNQIQQQQQQHQQQ---HQQQQ---------------QQQQQQQQLLQQQQQQ--TATTTTTKRRNAES
DvirGJ19245/46*  1 MHISAEVSSTTSNQTAHQQQQQQQQQHQQHQ-----------------QQQQQSTQQQLQQHTAATTTTGSTTKRRNAES
DyakGE17788      1 MVTSSSTSPTGNGWSSSTGDFKGITALATATGG------GAGGATQGATASTGATTAEVLAVSSSASVGSSSPTGGASNG
DpseGA22609      1 SMVTSSTSPTGNG-WSTPGDYKGATAATAPAAPSVV---TAATASTAAAAAAASVMCEVLPSSS-ASVGSSSPTGGASNG
DwilGK25373      1 SMVTSSTSPTGTAGWTTTGDYKVAT----------------VGSGGSSSAATAVAMCDVLPVSSNASVGSSSPTGGASNG
DperGL15806      1 QVLAAAAAAAAAAAVVNSGSSGVSVAQSTSLAN------TSTHSASSSTGSS--------TPDISATANTTANSSSSSNS
DyakGE15340/41*  1 MHLTTTSSNSTASNANNNNNN-----------------------------------NTANNNN-----TSSNNNTTNTNG
DgriGH17774/75*  1 MHLTTTSTNSTASNANSNNNNN----------------------------------NNNNNNNNNSNNTANNNNSSSSNN
DsimGD15530*     1 MHLTTTSSNSTASNANNNNNN-----------------------------------TANNNN------TSSNNNTTNTNG
DmeCG42267       1 MHISAEVSSTTSNQIQQ------------QQQQ---------------HQQQQQHQLLQHQQQQ--TATTTTTKRRNAES
DperGL15800/02*  1 MHISAEVSSTTSNQIQQQQQQQQ---------L---------------HQQLHQQHQQQTTPTATTTTGGSTTKRRNAES
DwilGK19810/11*  1 MHISAEVSSTTSNQQQQHQQQQQ------QHNP---------------SATATAAAATTTTTSTTPITTGSNTKRRNAET
DereGG19698      1 AAAAAAAAAAAAAVAQGPGLQQ-SSNAASA----------TASASAINPAQS-----------LANTSTHSASSTGSSTP
DmojGI15491      1 QVLAAAAAAAAAAAAATNVSQ-GNISTH------------SASSTGSSTPDNSHHSSSNNNNNNSSSTNNNNNSSSSNNN
DereGG18309      1 MVTSSSTSPTGNGWSSSTGDFKGITAVATATGG------GAGGATQGATASTGATVAEVMAVSSSASVGSSSPTGGASNG
DgriGH11831*     1 -MVTSSASPTGTG-WSSPGDYKSAS-----------------TAVAAAAVAAAAVMCEVLPSSS-ASVGSSSPTGGASNG
DperGL16499      1 SMVTSSTSPTGNG-WSTPGDYKGATAATAPAAPSVV---TAATASTAAAAAAASVMCEVLPSSS-ASVGSSSPTGGASNG
DwilGK19815      1 TQATAAAAAAAAAVANTQVLQAAAAAAAAVAAANVSSASTTASSTGSSTPDN-INNSSNNNNNNNNNNSHSNNNNNSSNN
DereGG17578      1 MHLTTTSSNSTASNANNNNNN-----------------------------------TANNNNN-----TSSNNNTTNTNG
DmojGI11519      1 MHLTTTSSNSTASNANSNNNNTNNN--------------------------------NNNNNN-----TANNNNTASSNN
DpseGA25684      1 --------------------------------------------------------------------------------
DanaGF15967      1 MHISAEVSSTTSNQIQQQHQQQQQQQQQQQQHQ---------------QQQQQQQQQHQQLQQQHQQTTNTTTKRRNAES
DsiGD17495       1 MHISAEVSSTTSNQIQQQQ----------QQQQ---------------HQQQQQHQLLQQQQQQ--TATTTTTKRRNAES
DgriGH17737/38*  1 MHISAEVSSTTSNQTHQQQQQHQQLQQQQH------------------QQQQQVTQQQHTTATTTITTTSSTTKRRNVES
DseGM22478       1 MVTSSSTSPTGNGWSSSTGDFKGITAVATATGG------GASGATQGATASTGATAAEVLAVSSSASVGSSSPTGGASNG
DanaGF21771      1 MVTSSSASPTGNGWTS-TGDFKGLTAVPAVVAA------TGASGGN----SAGGTTAKVLAISSSASVGSSSPTGGASNG
DvirGJ18742      1 -MVTSSASPTGTG-WSAPADYKSAT--------------TAAAAAAA----AAAVMCEVIPSSS-ASVGSSSPTGGASNG
DseGM23050       1 AAAAAAAAAAAAAVAQGPGPQQ-SNNVA------------TASAIAINPAQS-----------LANTSTHSASSTGSSTP
DanaGF15970      1 SSHTQVLQAAAAAAAAAAAISG-------------------ASSAGSSAANN-----------QANTSTHSASSTGSSTP
DpseGA14956      1 QVLAAAAAAAAAAAVVNSGSSGVSVAQSTSLAN------TSTHSASSSTGSS--------TPDISATANTTANSSSSSSS
DperGL15927      1 ---MANNNNNNSTTNNNNNST-----------------------------------TNNNNNS-----STANNNTSNTNG
DwilGK20052/54*  1 MHLTTTTSNTTSSTVNNNANN-----------------------------------NNNNNNTS--SNNSNNNNTSNTNG
DseGM23044/45*   1 MHISAEVSSTTSNQIQHQ-----------QQQQ---------------HQLLQ-----QQQQQQ--TATTTTTKRRNAES
DereGG19691      1 MHISAEVSSTTSNQIHQQQQQQQQQQQ-HQQQQ---------------QQQQQQHQLLQQQQQQQQTATTTTTKRRNAES
DmeCG34145       1 MHLTTTSSNSTASNANNNNNN-----------------------------------TANNNN------TSSNNNTTNTNG
DmojGI15484/86*  1 MHILAEVSSTTSNQTHQQQQQQQQQQQQQPT-----------------QQQQ-----HPAAATTTITTTASTTK------
DsiGD17500       1 AAAAAAAAAAAAAVAQGPGPQQ-SSNVA------------SASAIAINPAQS-----------LANTSTHSASSTGSSTP
DyakGE17894      1 AAAAAAAAAAAAAVAQGPGPQQQSSNAATATAT------ATASAIAINPAQS-----------LANTSTHSASSTGSSTP
Dmelz            1 MVTSSSTSPTGNGWSSSTGDFKGITAVATATGG------GVGGATQGATASTGATAAEVLAVSSSASVGSSSPTGGASNG
DsiGD16951*      1 --------------------------------------------------------------------------------
DgriGH17740      1 QVLAAAAAAAAAAAAATNVGHPGNISTH------------SASSTGSSTPDN-------TNNNSSNTSSNNNNTSSSNNN
DmojGI14398      1 AMVTSSASPTGTG-WSAPAEYKSAN--------------TAVAAATAGGSAAAAIMCEVLPSSS-ASVGSSSPTGGASNG
```

```
DmeRun            57 DLSTNNTSSSS-----------NATTSPQNS----------------AKMPSSMTDMFASLHEMLQEYHGELAQTGSPSI
DvirGJ19252       54 --SSNNNNNNS----------NS---NAANL----------------AKMPSSMTDMFASLHEMLQEYHGELAQTGSPSI
DanaGF15983       47 GSNNNNNNT---------SSNNNNNSNNNNNTEQNTPPTPAQLLNEAYTKMTSDILAERTLGDFLTEHPGELIRTSSPLF
DvirGJ18917*      42 NN-TSNTNG--------SNNNNSSSSNNN--TEQNTPPTPAQLLNEAYTKMTSDILAERTLGDFLTEHPGELIRTSSPLF
DseGM22662/63*    40 SSNN--TSG----------NNNNSSGNN-SNTEQNTPPTPAQLLNEAYTKMTSDILAERTLGDFLTEHPGELIRTSSPLF
DpseGA13742/42*   66 SSNNNNNNSNN---NNNNNSTTNNNNNNNNNNNNNSSNNAKTKPVDTSPYLTPENLIERTVDVLLAEHPGELVKTGSPHV
DyakGE17890       61 SASNNNNNTS----TTNNNNNNNNNNSTTNNNNNNNNN-VKTKPVDTSPYLTPENLIERTVDVLLAEHPGELVKTGSPHV
DvirGJ19245/46*   64 NNNNNNNNNSN---SNNNN----NNSNNNNSNNNNTNSSSKNKPVDTSPYLTPENLIERTVDVLLAEHPGELVKTGSPHV
DyakGE17788       75 TAHSGHSGHTGG-------HSSSTASNNNNIGASNSNSNSN--NNNNNAVHQDLLWMERLVQKRQQEHPGELVRTSNPYF
DpseGA22609       76 TAHSGHSGGGNS---SSTTIATTTNNNNNNNSNNSNNNNNN-----NNAVHQDLLWMERLVLKRQQEHPGELVRTSNPYF
DwilGK25373       65 TANSGHSGGGGG--------GNGGNSNSNSNNNSNNNNNNN-----NNAVHQDLLWMERYVAERQLEYPGELVRTSNPYF
DperGL15806       67 NSNTNTANSTA-----------NNGSSNSNS----------------AKMPSSMTDMFASLHEMLQEYHGELAQTGSPSI
DyakGE15340/41*   41 SSNNNNTSG--------NNNNNNSSGNNNSNTEQNTPPTPAQLLNEAYTKMTSDILAERTLGDFLTEHPGELIRTSSPLF
DgriGH17774/75*   47 NNNTSNTNG--------SNNNNSSSSNNNNSTEQNTPPTPAQLLNEAYTKMTSDILAERTLGDFLTEHPGELIRTSSPLF
DsimGD15530*      40 SSNNN-TSG----------NNNNSSGNN-SNTEQNTPPTPAQLLNEAYTKMTSDILAERTLGDFLTEHPGELIRTSSPLF
DmeCG42267        52 SASSNNNNNNN-TSTTNNNNTNNNNNSTTNNNNNNNNNNVKTKPVDTSPYLTPENLIERTVDVLLAEHPGELVKTGSPHV
DperGL15800/02*   57 SSNNNNNNSSS---NNNNN------NNSNNNNNNSSSNNAKTKPVDTSPYLTPENLIERTVDVLLAEHPGELVKTGSPHV
DwilGK19810/11*   60 NNNNNNNNNNN---------------------NNNNNSNAKTKPVDTSPYLTPENLIERTVDVLLAEHPGELVKTGSPHV
DereGG19698       59 DLSTNNTSSSS-----------NATTSPQNS----------------AKMPSSMTDMFASLHEMLQEYHGELAQTGSPSI
DmojGI15491       68 NTSSNNNNNSS----------NS---SNANA----------------AKMPSSMTDMFASLHEMLQEYHGELAQTGSPSI
DereGG18309       75 TAHSGHSGHTGG-------HSSSSASHNNNNGASNSNSNSNSNNNNNNAVHQDLLWMERLVQKRQQEHPGELVRTSNPYF
DgriGH11831*      61 TANSAHSASGNK----------NNSNNNNNN---------------NNAVHQDLLWMERFVRERQQEYPGELVRTSNPYF
DperGL16499       76 TAHSGHSGGGQQ---QQHHYCHHHHQQHNNN-----NNNNN-----NNAVHQDLLWMERLVLKRQQEHPGELVRTSNPYF
DwilGK19815       80 NNNTNSSSTSSSS--------ASSSSASSSS----------------AKMPSSMTDMFASLHEMLQEYHGELAQTGSPSI
DereGG17578       41 SSNNN-TSGGG-----NNNNNNNSSGNN-SNTEQNTPPTPAQLLNEAYTKMTSDILAERTLGDFLTEHPGELIRTSSPLF
DmojGI11519       44 NNNNSNTNG---------SNNNNSSSSNNNNTEQNTPPTPAQLLNEAYTKMTSDILAERTLGDFLTEHPGELIRTSSPLF
DpseGA25684        1 --------------------------------------------------MTSDILAERTLGDFLTEHPGELIRTSSPLF
DanaGF15967       66 SSNNNNNNNTTNNNKNNNNNSNNNNNNNNTTNNNNNNNNVKTKAVDTSPYLTPENLIERTVDVLLAEHPGELVKTGSPHV
DsiGD17495        54 SASSNNNNNN--TSTTNNNNTNNNNNSTTNNNNNNNN--VKTKPVDTSPYLTPENLIERTVDVLLAEHPGELVKTGSPHV
DgriGH17737/38*   64 NNNNNSTTNNN---NNNNN----NNNNNN---------SSKSKPVDTSPYLTPENLIERTVDVLLAEHPGELVKTGSPHV
DseGM22478        75 TAHSGHSGHTGG-------HSSSTASNSNNNGASNSN--------NNNAVHQDLLWMERLVQKRQQEHPGELVRTSNPYF
DanaGF21771       70 TAHSGHSGST----------TSNSGNNNNNNSNSNNNSNSN---NNNNALHQDLKWMERLVMKRQQEHPGELVRTSNPYF
DvirGJ18742       60 TANSTHSGS-----------NKNNNNNNNNSRN--NNNNNS-----NNAVHQDLLWMERFVLERQQEYPGELVRTSNPYF
DseGM23050        57 DLSTNNTSSSS-----------NATTSPQNS----------------AKMPSSMTDMFASLHEMLQEYHGELAQTGSPSI
DanaGF15970       51 DLNTANISTSTTSSSSTASSANNNSSSNTNS----------------AKMPSSMTDMFASLHEMLQEYHGELAQTGSPSI
DpseGA14956       67 NS--NTANNTA-----------NNGSSNSNS----------------AKMPSSMTDMFASLHEMLQEYHGELAQTGSPSI
DperGL15927       38 TNNTN---------------------TNNNNTEQNTPPTPAQLLNEAYTKMTSDILAERTLGDFLTEHPGELIRTSSPLF
DwilGK20052/54*   44 SNNNNNNNS--------SSSNNNNNNNNNNNTEQNTPPTPAQLLNEAYTKMTSDILAERTLGDFLTEHPGELIRTSSPLF
DseGM23044/45*    49 SASSNNNNN-----------TNNNNNSTTNNNNNNNN--VKTKPVDTSPYLTPENLIERTVDVLLAEHPGELVKTGSPHV
DereGG19691       65 SASNNNNNNTS-TTNNNNNTSNNNNNNNSTTNNNNNNN-VKTKPVDTSPYLTPENLIERTVDVLLAEHPGELVKTGSPHV
DmeCG34145        40 SSNNN-TSG----------NNNNSSGNN-SNTEQNTPPTPAQLLNEAYTKMTSDILAERTLGDFLTEHPGELIRTSSPLF
DmojGI15484/86*   53 --------------------------------------------LDTSPYLTPENLIERTVDVLLAEHPGELVKTGSPHI
DsiGD17500        57 DLSTNNTSSSS-----------NATTSPQNS----------------AKMPSSMTDMFASLHEMLQEYHGELAQTGSPSI
DyakGE17894       64 DLSTNNTSSSS-----------NATTSPQNS----------------AKMPSSMTDMFASLHEMLQEYHGELAQTGSPSI
Dmelz             75 TAHSGHSGHTGG-------HSSSTASNNNNNGASNSNSN------NNNAVHQDLLWMERLVQKRQQEHPGELVRTSNPYF
DsiGD16951*        1 -----------------------------------------------------------------------LVRTSNPYF
DgriGH17740       62 SSNSNNNNSSS----------NTSAASAANS----------------AKMPSSMTDMFASLHEMLQEYHGELAQTGSPSI
DmojGI14398       65 TANSANSGSGNKININNNNNNNSSNNNNNNTNSRNNNNNS-----NNAVHQDLLWMERFVLERQQEYPGELVRTSNPYF
```

```
DmeRun           110 LCSALPNHWRSNKSLPGAFKVIALDDVPDGTLVSIKCGNDENYCGELRNCTTTMKNQVAKFNDLRFVGRSGRGKSFTLTI
DvirGJ19252      103 LCSALPNHWRSNKSLPGAFKVIALDDVPDGTLVSIKCGNDENYCGELRNCTTTMKNQVAKFNDLRFVGRSGRGKSFTLTI
DanaGF15983      118 VCTVLPPHWRSNKTLPVAFKVVSLGDIMDGTMVTVRAGNDENYCAELRNCTAVMKNQVAKFNDLRFVGRSGRGKSFTLTI
DvirGJ18917*     111 VCTVLPPHWRSNKTLPVAFKVVSLGDIMDGTMVTVRAGNDENYCAELRNCTAVMKNQVAKFNDLRFVGRSGRGKSFTLTI
DseGM22662/63*   107 VCTVLPPHWRSNKTLPVAFKVVSLGDIMDGTMVTVRAGNDENYCAELRNCTAVMKNQVAKFNDLRFVGRSGRGKSFTLTI
DpseGA13742/42*  143 VCTTLPTHWRSNKTLPIAFKVLALGEVMDGTIVTIRAGNDENFCGELRNCTAVMKNQVAKFNDLRFVGRSGRGKESTQVD
DyakGE17890      136 VCTTLPTHWRSNKTLPIAFKVLALGEVMDGTIVTIRAGNDENFCGELRNCTAVMKNQVAKFNDLRFVGRSGRGKSFTLTI
DvirGJ19245/46*  137 VCTTLPTHWRSNKTLPIAFKVLALGEVMDGTIVTIRAGNDENFCGELRNCTAVMKNQVAKFNDLRFVGRSGRGKSFTLTI
DyakGE17788      146 LCSALPAHWRSNKTLPMAFKVVALAEVGDGTYVTIRAGNDENCCAELRNFTTQMKNDVAKFNDLRFVGRSGRGKSFTLTI
DpseGA22609      148 LCSALPSHWRSNKTLPLAFKVVALAEVGDGTYVTIRAGNDENCCAELRNCTAQMKNDVAKFNDLRFVGRSGRGKSFTLTI
DwilGK25373      132 LCSALPSHWRSNKTLPLAFKVVALADVGDGTYVTIRAGNDENCCAELRNCTAQMKNCVAKFNDLRFVGRSGRGKSFTLTI
DperGL15806      120 LCSALPNHWRSNKSLPGAFKVIALDDVPDGTLVSIKCGNDENYCGELRNCTTTMKNQVAKFNDLRFVGRSGRGKSFTLTI
DyakGE15340/41*  113 VCTVLPPHWRSNKTLPVAFKVVSLGDIMDGTMVTVRAGNDENYCAELRNCTAVMKNQVAKFNDLRFVGRSGRGKSFTLTI
DgriGH17774/75*  119 VCTVLPPHWRSNKTLPVAFKVVSLGDIMDGTMVTVRAGNDENYCAELRNCTAVMKNQVAKFNDLRFVGRSGR--------
DsimGD15530*     108 VCTVLPPHWRSNKTLPVAFKVVSLGDIMDGTMVTVRAGNDENYCAELRNCTAVMKNQVAKFNDL------SVGKSFTLTI
DmeCG42267       131 VCTTLPTHWRSNKTLPIAFKVLALGEVMDGTIVTIRAGNDENFCGELRNCTAVMKNQVAKFNDLRFVGRSGRGKSFTLTI
DperGL15800/02*  128 VCTTLPTHWRSNKTLPIAFKVLALGEVMDGTIVTIRAGNDENFCGELRNCTAVMKNQVAKFNDLRFVGRSGR-KDSTPVA
DwilGK19810/11*  119 VCTTLPTHWRSNKTLPIAFKVLALGEVMDGTIVTIRAGNDENFCGELRNCTAVMKNQVAKFNDLRFVGRSGRGKSFTLTI
DereGG19698      112 LCSALPNHWRSNKSLPGAFKVIALDDVPDGTLVSIKCGNDENYCGELRNCTTTMKNQVAKFNDLRFVGRSGRGKSFTLTI
DmojGI15491      119 LCSALPNHWRSNKSLPGAFKVIALDDVPDGTLVSIKCGNDENYCGELRNCTTTMKNQVAKFNDLRFVGRSGRGKSFTLTI
DereGG18309      148 LCSALPAHWRSNKTLPMAFKVVALAEVGDGTYVTIRAGNDENCCAELRNFTTQMKNDVAKFNDLRFVGRSGRGKSFTLTI
DgriGH11831*     116 LCSALPTHWRSNKTLPLAFKVVALADVGDGTYVTIRAGNDENCCAELRNYTAQMKNGVAKFNDLRLRGTQWSRNGSDRRR
DperGL16499      143 LCSALPSHWRSNKTLPLAFKVVALAEVGDGTYVTIRAGNDENCCAELRNCTAQMKNDVAKFNDLRFVGRSGRGKSFTLTI
DwilGK19815      136 LCSALPNHWRSNKSLPGAFKVIALDDVPDGTLVSIKCGNDENYCGELRNCTTTMKNQVAKFNDLRFVGRSGRGKSFTLTI
DereGG17578      114 VCTVLPPHWRSNKTLPVAFKVVSLGDIMDGTMVTVRAGNDENYCAELRNCTAVMKNQVAKFNDLRFVGRSGRGKSFTLTI
DmojGI11519      115 VCTVLPPHWRSNKTLPVAFKVVSLGDIMDGTMVTVRAGNDENYCAELRNCTAVMKNQVAKFNDLRFVGRSGRGKSFTLTI
DpseGA25684       31 VCTVLPPHWRSNKTLPVAFKVVSLGDIMDGTMVTVRAGNDENYCAELRNCTAVMKNQVAKFNDLRFVGRSGRGKSFTLTI
DanaGF15967      146 VCTTLPTHWRSNKTLPIAFKVLALGEVMDGTIVTIRAGNDENFCGELRNCTAVMKNQVAKFNDLRFVGRSGRGKSFTLTI
DsiGD17495       130 VCTTLPTHWRSNKTLPIAFKVLALGEVMDGTIVTIRAGNDENFCGELRNCTAVMKNQVAKFNDLRFVGRSGRGKSFTLTI
DgriGH17737/38*  128 VCTTLPTHWRSNKTLPIAFKVLALGEVMDGTIVTIRAGNDENFCGELRNCTAVMKNQVAKFNDLRFVGRSGRGKSFTLTI
DseGM22478       140 LCSALPAHWRSNKTLPMAFKVVALAEVGDGTYVTIRAGNDENCCAELRNFTTQMKNDVAKFNDLRFVGRSGRGKSFTLTI
DanaGF21771      137 LCSALPSHWRSNKTLPLAFKVVALAEVGDGTYVTIRAGNDENCCAELRNCTAQMKNDVAKFNDLRFVGRSGRGKSFTLTI
DvirGJ18742      122 LCSALPSHWRSNKTLPLAFKVVALADVGDGTYVTIRAGNDENCCAELRNYTAQMKNGVAKFNDLRFVGRSGRGKSFTLTI
DseGM23050       110 LCSALPNHWRSNKSLPGAFKVIALDDVPDGTLVSIKCGNDENYCGELRNCTTTMKNQVAKFNDLRFVGRSGRGKSFTLTI
DanaGF15970      115 LCSALPNHWRSNKSLPGAFKVIALDDVPDGTLVSIKCGNDENYCGELRNCTTTMKNQVAKFNDLRFVGRSGRGKSFTLTI
DpseGA14956      118 LCSALPNHWRSNKSLPGAFKVIALDDVPDGTLVSIKCGNDENYCGELRNCTTTMKNQVAKFNDLRFVGRSGRGKSFTLTI
DperGL15927       97 VCTVLPPHWRSNKTLPVAFKVVSLGDIMDGTMVTVRAGNDENYCAELRNCTAVMKNQVAKFNDLRFVGRSGRGKSFTLTI
DwilGK20052/54*  116 VCTVLPPHWRSNKTLPVAFKVVSLGDIMDGTMVTVRAGNDENYCAELRNCTAVMKNQVAKFNDLRFVGRSGRGASNTHWL
DseGM23044/45*   116 VCTTLPTHWRSNKTLPIAFKVLALGEVMDGTIVTIRAGNDENFCGELRNCTAVMKNQVAKFNDLRFVGRSGRGKSFTLTI
DereGG19691      143 VCTTLPTHWRSNKTLPIAFKVLALGEVMDGTIVTIRAGNDENFCGELRNCTAVMKNQVAKFNDLRFVGRSGRGKSFTLTI
DmeCG34145       108 VCTVLPPHWRSNKTLPVAFKVVSLGDIMDGTMVTVRAGNDENYCAELRNCTAVMKNQVAKFNDLRFVGRSGRGKSFTLTI
DmojGI15484/86*   89 VCTTLPTHWRSNKTLPIAFKVLALGEVMDGTIVTIRAGNDENFCGELRNCTAVMKNQVAKFNDLRFVGRSGRGKSFTLTI
DsiGD17500       110 LCSALPNHWRSNKSLPGAFKVIALDDVPDGTLVSIKCGNDENYCGELRNCTTTMKNQVAKFNDLRFVGRSGRGKSFTLTI
DyakGE17894      117 LCSALPNHWRSNKSLPGAFKVIALDDVPDGTLVSIKCGNDENYCGELRNCTTTMKNQVAKFNDLRFVGRSGRGKSFTLTI
Dmelz            142 LCSALPAHWRSNKTLPMAFKVVALAEVGDGTYVTIRAGNDENCCAELRNFTTQMKNDVAKFNDLRFVGRSGRGKSFTLTI
DsiGD16951*       10 LCSALPAHWRSNKTLPMAFKVVALAEVGDGTYVTIRAGNDENCCAELRNFTTQMKNDVAKFNDLRFVGRSGRGKSFTLTI
DgriGH17740      116 LCSALPNHWRSNKSLPGAFKVIALDDVPDGTLVSIKCGNDENYCGELRNCTTTMKNQVAKFNDLRFVGRSGRGKSFTLTI
DmojGI14398      140 LCSALPSHWRSNKTLPLAFKVVALADVGDGTYVTIRAGNDENCCAELRNYTAQMKNGVAKFNDLRFVGRSGRGKSFTLTI
```

```
DmeRun            190 TIATYPVQIASYSKAIKVTVDGPREPRSKQS-YGYPHPGAFNPFMLNPAWLDAAYMTYGYADYFRHQAAAQAAQVHHPAL
DvirGJ19252       183 TIATYPVQIASYSKAIKVTVDGPREPRSKQS-YGYPHPGAFNPFMLNPAWLDAAYMTYGYADYFRHQQAA-AAAVHHPAL
DanaGF15983       198 TVSTNPPHIATYNKAIKVTVDGPREPRSKT-RQQQQFHFAFGQRPFHFSTDPLSGFR-MPPIG--------NCQS----A
DvirGJ18917*      191 TVSTNPPHIATYNKAIKVTVDGPREPRSKT-RQQQQFHFAFGQRPFHFSTDPLSGFR-MPPIG--------NCQS----A
DseGM22662/63*    187 TVSTNPPHIATYNKAIKVTVDGPREPRSKT-RQQQQFHFAFGQRPFHFSTDPLSGFR-MPPIG--------NCQS----A
DpseGA13742/42*   223 VIPTNQHEIATYTKAIKVTVDGPREPRSKV-RHQGFHPFAFG--PQRFGPDPLMAGLPFKLPGFAHHLVGMSHLHAPDWR
DyakGE17890       216 VISTNPIQIATYTKAIKVTVDGPREPRSKV-RHQGFHPFAFG--PQRFGPDPLMAGLPFKLPGFAHHLVGMSHLHAPDWR
DvirGJ19245/46*   217 VISTNPIQIATYTKAIKVTVDGPREPRSKV-RHQGFHPFAFG--PQRFGPDPLMAGLPFKLP------------------
DyakGE17788       226 TVATSPPQVATYAKAIKVTVDGPREPRSKTSPTGGPHYRALGLGQRPYIDGFPSTKALHELETLRRSAKVAAVTTAAAAA
DpseGA22609       228 TVATSPPQVATYAKAIKVTVDGPREPRSKTSPTGGPHYRALGLGQRPYIDGFP--KTLHELETLRRSAKVAAATTAAAAA
DwilGK25373       212 TVATSPPQVATYAKAIKVTVDGPREPRSKTSPTGGPHYR-IGLTP----LGFP--KTLHELESLRRSAKVAAATTAVASV
DperGL15806       200 TIATYPVQIASYSKAIKVTVDGPREPRSKQS-YGYPHPGAFNPFMLNPAWLDAAYMTYGYADYFRHQAAA-QAAVHHPAL
DyakGE15340/41*   193 TVSTNPPHIATYNKAIKVTVDGPREPRSKT-RQQQQFHFAFGQRPFHFSTDPLSGFR-MPPIG--------NCQS----A
DgriGH17774/75*   191 ---------ATYNKAIKVTVDGPREPRSKT-RQQQQFHFAFGQRPFHFSTDPLSGFR-MPPIG--------NCQS----A
DsimGD15530*      182 TVSTNPPHIATYNKAIKVTVDGPREPRSKTSKQQQQFHFAFGQRPFHFSTDPLSGFR-MPPIG--------NCQS----A
DmeCG42267        211 VISTNPIQIATYTKAIKVTVDGPREPRSKV-RHQGFHPFAFG--PQRFGPDPLMAGLPFKLPGFAHHLVGMSHLHAPDWR
DperGL15800/02*   207 VVPTNKNEIATYTKAIKVTVDGPREPRSKV-RHQGFHPFAFG--PQRFGPDPLMAGLPFKLPGFAHHLVGMSHLHAPDWR
DwilGK19810/11*   199 VISTNPIQIATYTKAIKVTVDGPREPRSKV-RHQGFHPFAFG--PQRFGPDPLMAGLPFKLPDVS---------------
DereGG19698       192 TIATYPVQIASYSKAIKVTVDGPREPRSKQS-YGYPHPGAFNPFMLNPAWLDAAYMTYGYADYFRHQAAAQAAQVHHPAL
DmojGI15491       199 TIATYPVQIASYSKAIKVTVDGPREPRSKQS-YGYPHPGAFNPFMLNPAWLDAAYMTYGYADYFRHQQAA-AAAVHHPAL
DereGG18309       228 TVATSPPQVATYAKAIKVTVDGPREPRSKTSPTGGPHYRALGLGQRPYIDGFPSTKALHELETLRRSAKVAAVTTAAAAA
DgriGH11831*      196 RMDRLDQQRVDQAAEARLLISSQVAHQS----------------------------IHKFLN-RQNGKLAEIAKQVADF
DperGL16499       223 TVATSPPQVATYAKAIKVTVDGPREPRSKTSPTGGPHYRALGLGQRPYIDGFP--KTLHELETLRRSAKVAAATTAAAAA
DwilGK19815       216 TIATYPVQIASYSKAIKVTVDGPREPRSKQS-YGYPHPGAFNPFMLNPAWLDAAYMTYGYADYFRHQAAA--AAVHHPAL
DereGG17578       194 TVSTNPPHIATYNKAIKVTVDGPREPRSKT-RQQQQFHFAFGQRPFHFSTDPLSGFR-MPPIG--------NCQS----A
DmojGI11519       195 TVSTNPPHIATYNKAIKVTVDGPREPRSKT-RQQQQFHFAFGQRPFHFSTDPLSGFR-MPPIG--------NCQS----A
DpseGA25684       111 TVSTNPPHIATYNKAIKVTVDGPREPRSKT-RQQQQFHFAFGQRPFHFSTDPLSGFR-MPPIG--------NCQS----A
DanaGF15967       226 VISTNPIQIATYTKAIKVTVDGPREPRSKV-RHQGFHPFAFG--PQRFGPDPLMAGLPFKLPGFAHHLVGMSHLHAPDWR
DsiGD17495        210 VISTNPIQIATYTKAIKVTVDGPREPRSKV-RHQGFHPFAFG--PQRFGPDPLMAGLPFKLPGFAHHLVGMSHLHAPDWR
DgriGH17737/38*   208 VISTNPIQIATYTKAIKVTVDGPREPRSKV-RHQGFHPFAFG--PQRFGPDPLMAGLPFKLPGFAHHLVGMSHLHAPDWR
DseGM22478        220 TVATSPPQVATYAKAIKVTVDGPREPRSKTSPTGGPHYRALGLGQRPYIDGFPSTKALHELETLRRSAKVAAVTTAAAAA
DanaGF21771       217 TVATSPPQVATYAKAIKVTVDGPREPRSKTSPTGGPHYRALGLGQRPYIDGFP--KSLHELEALRRTTK--AVTTAAAAA
DvirGJ18742       202 TVATSPPQVATYAKAIKVTVDGPREPRSKTSPPGGPQYRALGLGQRPFIDSFP--KTFHELETLRRSAKVAAATTAAAAA
DseGM23050        190 TIATYPVQIASYSKAIKVTVDGPREPRSKQS-YGYPHPGAFNPFMLNPAWLDAAYMTYGYADYFRHQAAAQAAQVHHPAL
DanaGF15970       195 TIATYPVQIASYSKAIKVTVDGPREPRSKQS-YGYPHPGAFNPFMLNPAWLDAAYMTYGYADYFRHQAAAQAAQVHHPAL
DpseGA14956       198 TIATYPVQIASYSKAIKVTVDGPREPRSKQS-YGYPHPGAFNPFMLNPAWLDAAYMTYGYADYFRHQAAA-QAAVHHPAL
DperGL15927       177 TVSTNPPHIATYNKAIKVTVDGPREPRSKT-RQQQQFHFAFGQRPFHFSTDPLSGFR-MPPIG--------NCQS----A
DwilGK20052/54*   196 SSCTTPTSAQFNNPALGFTCSSPMLPDSTASDLDQHLSSLVGSTSGQMTHHSLLGATGQTSISSTVNGASANVHSSQNGA
DseGM23044/45*    196 VISTNPIQIATYTKAIKVTVDGPREPRSKV-RHQGFHPFAFG--PQRFGPDPLMAGLPFKLPGFAHHLVGMSHLHAPDWR
DereGG19691       223 VISTNPIQIATYTKAIKVTVDGPREPRSKV-RHQGFHPFAFG--PQRFGPDPLMAGLPFKLPGFAHHLVGMSHLHAPDWR
DmeCG34145        188 TVSTNPPHIATYNKAIKVTVDGPREPRSKT-RQQQQFHFAFGQRPFHFSTDPLSGFR-MPPIG--------NCQS----A
DmojGI15484/86*   169 VISTNPIQIATYTKAIKVTVDGPREPRSKV-RHQGFHPFAFG--PQRFGPDPLMAGLPFKLPGFAHHLVGMSHLHAPDWR
DsiGD17500        190 TIATYPVQIASYSKAIKVTVDGPREPRSKQS-YGYPHPGAFNPFMLNPAWLDAAYMTYGYADYFRHQAAAQAAQVHHPAL
DyakGE17894       197 TIATYPVQIASYSKAIKVTVDGPREPRSKQS-YGYPHPGAFNPFMLNPAWLDAAYMTYGYADYFRHQAAAQAAQVHHPAL
Dmelz             222 TVATSPPQVATYAKAIKVTVDGPREPRSKTSPTGGPHYRALGLGQRPYIDGFPSTKALHELESLRRSAKVAAVTTAAAAA
DsiGD16951*        90 TVATSPPQVATYAKAIKVTVDGPREPRSKTSPTGGPHYRALGLGQRPYIDGFPSTKALHELETLRRSAKVAAVTTAAAAA
DgriGH17740       196 TIATYPVQIASYSKAIKVTVDGPREPRSKQS-YGYPHPGAFNPFMLNPAWLDAAYMTYGYADYFRHQQAA-AAAVHHPAL
DmojGI14398       220 TVATSPPQVATYAKAIKVTVDGPREPRSKTSPTGGPQYRALGLGQRPFIDGFP--KTFHELETLRHSAKVAAATTAAAAA
```

```
DmeRun          269 AKSSASSVSPNPNPSVATSS------------SSAVQP------SEYPHPAAAVAAAA------GQPAAMMPSPPGAAPA
DvirGJ19252     261 SKAATSPNGSASGVVSPGTA-------AAGAAVPAAAA------ADYPPPPSSVGVGVG----AVAPGSMMPSPPGGPPA
DanaGF15983     264 SNTHWGYGSAASAYSPYLASSG--------LSSCTTPTSAQFNNPALGFTCSSNDQTNNQDFGGATNRDCVP-MLPDSTA
DvirGJ18917*    257 SNTHWGYGSAASAYSPYLASSG--------LSSCTTPTSAQFNNPALGFTCSSNDQSNNQDFGGATNRDCVP-MLPDSTA
DseGM22662/63*  253 RYTHWGYGSAASAYSPYLASSG--------LSSCTTPTSAQFNNPALGFTCSSNDQSNNQDFGGATNRDCVP-MLPDSTA
DpseGA13742/42* 300 AHMALGGRSAGFPAGPFFGHH-----AAFPAASGLRGLSADGQQQQQHQQQQ--HQQQLATVGAAHSTTSPEGSPTTTTS
DyakGE17890     293 AHMALGGRPAAFTAAPFFGHH----AAAFPTASGLRGLSGD-----NQQHQQQQQ--QLATVGAAHSTTSPEGSPTTTTT
DvirGJ19245/46* 276 ---------------------------------------------------DNQQQLPTVGAAHSTTSPEGSPTTTTS
DyakGE17788     306 ATAASAANAVAVAAAAVAVTPTGGGVG---GVGGVAGGQGAGVGVGAGLVQQLSSNYSSP------NSTINSDCQVYKPN
DpseGA22609     306 ATAATAATAVAAAASVALPGSTTTTG---GVGVGVGVGGGSSGSGAGLVQQLSSNYSSP------NSTINSDCQVYKPN
DwilGK25373     285 A------------AASVVTAAAVSTTSG-------------STSTTSLVQQLSSNYSSS------NSTINSDCPGYKPN
DperGL15806     278 SKASPSSSSSIVSPSASAAASSG-------AGNGAAGP------ADYHHVSQITPPPSRG-TNGRWPTGCHDALAAQEPL
DyakGE15340/41* 259 SNTHWGYGSAASAYSPYLASSG--------LSSCTTPTSAQFNNPALGFTCSSNDQSNNQDFGGATNRDCVP-MLPDSTA
DgriGH17774/75* 248 SNTHWGYGSAASAYSPYLASSG--------LSSCTTPTSAQFNNPALGFTCSSNDQSNNQDFGGATNRDCVP-MLPDSTA
DsimGD15530*    249 SNTHWGYGSAASAYSPYLASSG--------LSSCPTPTSAQFHNPPLGFTCSSNDQSNNQDFGGATNRDCVPAVLPDSTA
DmeCG42267      288 AHMALGGRPAAFTAAPFFGHH----AAAFPTASGLRGLSGD-----SQQHQQQQQQHQLATVGAAHSTTSPEGSPTTTTT
DperGL15800/02* 284 AHMALGGRSAGFPAGPFFGHH-----AAFPAASGLRGLSADGQQQQQHQQQQQQHQQQLATVGAAHSTTSPEGSPTTTTS
DwilGK19810/11* 261 -------------------------------------------QEQQQQQQHQHLATVGPAHSTTSPEGSPTATPT
DereGG19698     271 AKSSASSVSPNPNPSVATSS------------SSAVQP------SEYPHPAAAVAAAAAA----GQPAAMMPSPPGAAPA
DmojGI15491     277 SKAATSPNGSNSGVISPGAAGGAAMPTAASAVVAAAAA------ADYPPVPPVVGVPVGG---VVPPASMMPSPPGGPPA
DereGG18309     308 ATAASAANAVAAAAAAVAVAPAGGGVA-----GGVAGG------AGAGLVPQLSSNYSSP------NSTINSDCQVYKPN
DgriGH11831*    246 E---------------------RESN--------------------ALTEELGEVPKSQ------LCPL-ADWRAWLSQ
DperGL16499     301 ATAATAATAVAAAASVALPGSTTTGGVGVGVGVGVGVGGGSSGSGAGLVQQLSSNYSSP------NSTINSDCQVYKPN
DwilGK19815     293 AKTSPGGGAPPPSGGGGAAP---------------TPP-------DYHHVSQLTPPPAG------APSNQPATGSATALA
DereGG17578     260 SNTHWGYGSAASAYSPYLASSG--------LSSCTTPTSAQFNNPALGFTCSSNDQSNNQDFGGATNRDCVP-MLPDSTA
DmojGI11519     261 SNTHWGYGSAASAYSPYLASSG--------LSSCTTPTSAQFNNPALGFTCSSNDQTNNQDFSGATNRDCVP-MLPDSTA
DpseGA25684     177 SNTHWGYGSAASAYSPYLASSG--------LSSCTTPTSAQFNNPALGFTCSSNDQSNNQDFGGATNRDCVP-MLPDSTA
DanaGF15967     303 AHMALGGRPAGFPAGPFFGHHAAAAAAAFPTASGLRGLSAA-----DNQQQQQQQ---QQQQQLAHSTTSPEGSPTTTTT
DsiGD17495      287 AHMALGGRPAAFTAAPFFGHH----AAAFPTASGLRGLSGD-----SQQHQQQQ---HLATVGAAHSTTSPEGSPTTTTT
DgriGH17737/38* 285 AHMALGGR-PGFPAGPFFAHH-----HGAAFPATAA----------SGIRGLSENQQPLATVGAAHSTTSPEGSPTTTTS
DseGM22478      300 ATAASAANAVAAAAAAVAVTPTGGGGG----AGGVAGG------AGAGLVQQLSSNYSSP------NSTINSDCQVYKPN
DanaGF21771     293 ATAATAATAVAAAAAVAVSPAGGGGG---GTVGVVGG------SGAGLVQQLNSNYSSP------NSTINSDCQVYKPN
DvirGJ18742     280 A-------------VSVASAGNTLTTN-------------------ASIAQQLGSNYSSS------NSTINSDCQGYKPN
DseGM23050      269 AKSSASSVSPNPNPSVATSS------------SSAVQP------SEYPHPAAAVAAAA------GQPAAMMPSPPGAAPA
DanaGF15970     274 AKAAAAQVSPVSAGSNSIPT------------AASVGAGAPGAGNVAPPPTGGDYHSGGA---PGQPAAMMPSPPGAAPA
DpseGA14956     276 SKASPSSSSSIVSPSASAAASSG-------AGNGAAGP------ADYHHVSQITPPPSGAPTAAGQPAAMMPSPPGAAPA
DperGL15927     243 SNTHWGYGSAASAYSPYLASSG--------LSSCTTPTSAQFNNPALGFTCSSNDQSNNQDFGGATNRDCVP-MLPDSTA
DwilGK20052/54* 276 SNTHWGYGSAASAYSPYLASSG--------LSSCTTPTSAQFNNPALGFTCSSNDQSNNQDFGGATNRDCVP-MLPDSTA
DseGM23044/45*  273 AHMALGGRPAAFTAAPFFGHH----AAAFPTASGLRGLSGD-----SQQHQQQQQ--HLATVGAAHSTTSPEGSPTTTTT
DereGG19691     300 AHMALGGRPAAFTTAPFFGHH----AAAFPTAGGLRGLSGD-----SQQHQQQQQQQQLATVGAAHSTTSPEGSPTTTTT
DmeCG34145      254 SNTHWGYGSAASAYSPYLASSG--------LSSCTTPTSAQFNNPALGFTCSSNDQSNNQDFGGATNRDCVP-MLPDSTA
DmojGI15484/86* 246 AHMALGGR-PGFPAGPFFAHH-----HGASFPGGAT----------GGLRGLSDNQQQMATVGAAHSTTSPEGSPTTTTS
DsiGD17500      269 AKSSASSVSPNPNPSVATSS------------SSAVQP------SEYPHPAAAVAAAA------GQPAAMMPSPPGAAPA
DyakGE17894     276 AKSSASSVSPNPNPSVATSS------------SSAVQP------AEYPHPAAAVAAAAAA---AGQPAAMMPSPPGAAPA
Dmelz           302 ATAASAANAVAAAAAAVAVTPTGGGGGV--AAGGVAGG------AGAGLVQQLSSNYSSP------NSTINSDCQVYKPN
DsiGD16951*     170 ATAASAANAVAAAAAAVAVTPTGGGGG----AGGVAGG------AGAGLVQQLSSNYSSP------NSTINSDCQVYKPN
DgriGH17740     274 SKAATSPNGSVSGVVSPAMP-------------NAGAS------SDYPPPMPSASSSSS---IVPPASMMPSPPGGAPA
DmojGI14398     298 A-------------VSVASAGNALTTN-------------------ASIAQQLGSNYSSS------NSTINSDCQGYKPN
```

```
DmeRun          325 --TPYAIPQFPFNHVAAAAAAKA---------ATPHAFHPYNFA--AAAGLRARN---AALHHQSEP------VHVSPAS
DvirGJ19252     324 --SAYAMPQFPFNHVAAAAAAAAHQNQ----KSTPHAFHPYNFA--AAAGLRARNAAAAALHHGGLSA--AEAAHMSPAS
DanaGF15983     335 SDLDQHLSSLVGSTSGQMTHHSL------LGATGQTSISSTVNGASGGSAGGGAGGAGGGAGAAGAGAAGGGGSNSILVP
DvirGJ18917*    328 SDLDQHLSSLVGSSSGQMSHHSL------LGASGQTSITSTVNGAGAGSGAT---------AGSATGAGGGGANSILVP
DseGM22662/63*  324 SDLDQHLSSLVGSTSGQMTHHSL------LGAGGQTSISSTVNGASGGGSAG--AGTAGGGAGSGGGAGGGAGGNSILVP
DpseGA13742/42* 373 GT----LSAFVQPAIHCPLGS---------PRSPPTILTSLQHDNNNN---SSNIDAGFESDSISVTGSPRKSISSPLTH
DyakGE17890     362 SG--TQLSAFVQPPMTSSPP------------PVTSLQHDNNNNNSNNNNSSSHIDAGFESDSISVTGSPRKSLASPLTH
DvirGJ19245/46* 303 AT----LSAFVQPPA-CNASA---------GGSPPLTLSSMQHHDNNNNNNSSSNIDAGFESDSISVTGSPRKSICS-LTH
DyakGE17788     377 APHIQGADMMGAGEWT-GSSSS-----------AAAYYHSHA-HHPHAHHAHAHLQ-----------HQMALPPPPPPPA
DpseGA22609     377 APHIQETDLMGAGEWTGSSSS------------AAAYYHSHAHHPHAH---HAHAHAHAHAHAHHLQHQMALPPPPPPPA
DwilGK25373     333 APHIQETE---SGEWTGSASSS---NVAYTVGVGVPYTHHHATH---HTHPHSHSHAAAAHHLQH-HHQMTLPPPPQPPA
DperGL15806     344 RQPPYAIPQFPFNHVAAAAAAAAQQQQQHHAKSTPHAFHPYNFA--AAAGLRARNAAAAVLHHGGDATG---ISHISPAS
DyakGE15340/41* 330 SDLDQHLSSLVGSTSGQMTHHSL------LGAGGQTSISSTVNGASGGGSAG--AGTAGGGAGSGGGAGGGAGGNSILVP
DgriGH17774/75* 319 SDLDQHLSSLVGSTSGQMSHHSL------LGASGQTSITSTVNGASAGSGAT---------AGSAAGAGSGGGGNSILVP
DsimGD15530*    321 SDLDQHLSSLVGSTSGQMTHHSL------LGAGGQTSISSTVNGASGGGSAG--AGTAGGGAGSGGGAGGGAGGNSILVP
DmeCG42267      359 SG--TQLSAFVQPPMTSSPP------------PVTSLQHDNNNNNSNNNNSSSHIDAGFESDSISVTGSPRKSLGSPLTH
DperGL15800/02* 359 GT----LSAFVQPAIHCPLGS---------PRSPPTILTSLQHDNNNN---SSNIDAGFESDSISVTGSPRKSISSPLTH
DwilGK19810/11* 294 TT-TTTSSAFVQPNSGSSLGLGLGVGVVGLGAGSPTVLTSLSSQDDNNN--HNIDDAGFESDSISVTGSPRKSLSS-LTQ
DereGG19698     329 --TPYAMPQFPFNHVAAAAAAKA---------ATPHAFHPYNFA--AAAGLRARN---AALHHQSEP------VHVSPAS
DmojGI15491     348 --SAYAMPQFPFNHVAAAAAAAAHQSQ----KATPHAFHPYNFA--AAAGLRARNAAAAALHHGGLSG--AETAHMSPAS
DereGG18309     371 APHIQGADMMGAGEWT-GSSSS-----------AAAYYHSHA-HHP-----HAHLQ-----------HQMALPPPPPPPA
DgriGH11831*    277 ALSVLQTQ------------------------------------------------------------------------
DperGL16499     375 APHIQETDLMGAGEWTGSSSS------------AAAYYHSHAHHPHAHHA-HAHAHAHAHAHAHHLQHQMALPPPPPPPA
DwilGK19815     345 -----STPQQPQHHHPAASSGMMP--------SPPPGAAAVPVP------------------------------ATAAAA
DereGG17578     331 SDLDQHLSSLVGSTSGQMTHHSL------LGAGGQTSISSTVNGASGGGSAG--AGTAGGGAGSGGGAGGGAGGNSILVP
DmojGI11519     332 SDLDQHLNSLP---------------------------------------------------------------------
DpseGA25684     248 SDLDQHLSSLVGSTSGQMTHHSL------LGATGQTSISSTVNGASAGSAGT--GGTGAGGAGS-----GGAGGNSILVP
DanaGF15967     375 TSGHTQLSAFVQPPIGCSSPV---------LAPLTSLQ-DNENDNNNNTNNNSNIDAGFESDSISVTGSPRKSLGSPLTH
DsiGD17495      355 SG--TQLSAFVQPPMTSSPP------------PVTSLQHDNNNNNSNNNNSSSHIDAGFESDSISVTGSPRKSLGSPLTH
DgriGH17737/38* 349 AT----LSAFVQPAA-CNASV---------GSSPPLTLSSMQHHDNNNNNNSSSNIDAGFESDSISVTGSPRKSISS-LTH
DseGM22478      364 APHIQAAEMMGAGEWTNGSSSS-----------AAAYYHSHA-HHPHAHHAHAHLQ-----------HQMALPPPPPPPA
DanaGF21771     358 APHIQETDLMGAGEWT-GSTST-----------AAAYYHSHAHHHPHAHHPHAHPHA------HLHQHQMALPPPPPPPA
DvirGJ18742     322 APQIQETDLIGAAEWTGSASSGANVAYPVGVGVGVPYHHAHHSHTHSHTHGHSAHH----HHAHHLQHQVALPPPPPPPA
DseGM23050      325 --TPYAIPQFPFNHVAAAAAAKA---------ATPHAFHPYNFA--AAAGLRARN---AALHHQSEP------VHVSPAS
DanaGF15970     339 --AAYAMPQFPFNHVAAAAAAAAHQHP----KATPHAFHPYNFATAAAVGLRARN---AALHHQSEAGVVAPLNHVSPAS
DpseGA14956     343 --AAYAIPQFPFNHVAAAAAAAAQQQQQHHAKSTPHAFHPYNFA--AAAGLRARNAAAAVLHHGGDATG---ISHISPAS
DperGL15927     314 SDLDQHLSSLVGSTSGQMTHHSL------LGATGQTSISSTVNGASAGSAGT--GGTGAGGAGS-----GGAGGNSILVP
DwilGK20052/54* 347 SDLDQHLSSLVGSTSGQMTHHSL------LGATGQTSISSTVNGASAGGATAG-SVTGGGTAGTGGSGGGGGATNSILVP
DseGM23044/45*  342 SG--TQLSAFVQPPMTSSPP------------PVTSLQHDNNNNNSNNNNSSSHIDAGFESDSISVTGSPRKSLGSPLTH
DereGG19691     371 SG--TQLSAFVQPPMTSSPP------------PVTSLQHDNNNNNNGNNNISSHIDAGFESDSISVTGSPRKSLGSPLTH
DmeCG34145      325 SDLDQHLSSLVGSTSGQMTHHSL------LGAGGQTSISSTVNGASGGGSAG--AGTAGGGAGSGGGAGGGAGGNSILVP
DmojGI15484/86* 310 AT----LSAFVQPPA-CNASA---------GGSPPLMLSSLQHHDNNNNNSSSNIDAGFESDSISVTGSPRKSISS-LTH
DsiGD17500      325 --TPYAMPQFPFNHVAAAAAAKA---------ATPHAFHPYNFA--AAAGLRARN---AALHHQSEP------VHVSPAS
DyakGE17894     335 --TPYAMPQFPFNHVAAAAAAKA---------ATPHAFHPYNFA--AAAGLRARN---AALHHQSEP------VHVSPAS
Dmelz           368 APHIQAAEMMGAGEWTNGSSSS-----------AAAYYHSHA-HHPHAHHAHAHLQ-----------HQMALPPPPPPPA
DsiGD16951*     234 APHIQDLIQFPR----VSKAST-----------TTAFFAIT---------------------------------------
DgriGH17740     332 --AAYAMPQFPFNHVAAAAAAAAHQSQ----KSTPHAFHPYNFA--AAAGLRARNAAAAALHHGGVP---ADAAHMSPAS
DmojGI14398     340 APQIQETDLIGAAEWTGSASSGANIAYPVGVGVGVPYHHAHHTHSHSHSHSHSHSHSHAAHHHHHLQHQVALPPPPPPPA
```

```
DmeRun            383 SRPSSSSPT--------------------------------------------------------------QQHVLLKLNT
DvirGJ19252       394 SRPSSSSPT--PTPTS-------------------------------------------------------MPHVLLKLNT
DanaGF15983       409 RYHTNTSN-EYNVHSSQN----------GPRSLSDSSQAESPVQEDLLSTNTPNLGS--------------TGAAGANGG
DvirGJ18917*      393 RYHTNASN-EYNVHSSQN----------GPRSLSDSSQAESPVQEDLLSTNTPNLGS---------------GSGAGASN
DseGM22662/63*    396 RYHTNASN-EYNVHSSQN----------GPRSLSDSSQAESPVQEDLLTTNTPNLGS--------------TAGGGAANG
DpseGA13742/42*   437 DEEEAEADAEAEAEAEAEAEPD-----GEAEAEAGTQLSGDARSLATISGRSAAGSL-------TETAPGSGGAFTALIQ
DyakGE17890       428 DEE--EAEAEAEADADAEA---------EEAEVGGLSLNGGGIQGTLHSESSPGS----------------GGAFTALIQ
DvirGJ19245/46*   368 DEEEPDP--------------------EAGRSRSPTLLSADASSVATLADPAPGS----------------GGAFTALIQ
DyakGE17788       433 AAPVSVGVGGNGATMG--MGMGVG----MGMNHYG---GGYDSANSLEAGQYAA---------------HLPAVLPEMHG
DpseGA22609       442 AAPVTNGAG--GMGVGVGMGVGMGMGVGMSMNHYG---SGYETASSLDAGNYA----------------HLPAVLPEMHG
DwilGK25373       403 ATPVAMGGQASGMGMGMGMGMGMGMSMGMTMNHYGSTMGGYDASY-----DTS----------------HLPTVLQDMHG
DperGL15806       419 SRPSSSSPT--------------------------------------------------------------QQHVLLKLNT
DyakGE15340/41*   402 RYHTNASN-EYNVHSSQNXXXXVHSSQNGPRSLSDSSQAESPVQEDLLTTNTPNLGS--------------TAGGGAANG
DgriGH17774/75*   384 RYHTNASN-EYNVHSSQN----------GPRSLSDSSQAESPVQEDLLSTNTPNLGS---------------GSGGGATN
DsimGD15530*      393 RYHTNASN-EYNVHSSQN----------GPRSLSDSSQAESPVQEDLLTTNTPNLGS--------------TAGGGAANG
DmeCG42267        425 DEEEAEAEAEAEAEAEAEA---------EEAEVGGLSRNGGGIQGPLHSESSPGS----------------GGAFTALIQ
DperGL15800/02*   423 DEEEAEADAEAEAEAEAEAEPD-----GEAEAEPGTQLSGDARSLATISGRS----------------------------
DwilGK19810/11*   370 EDE--------------------------EADADGDGTEAESTLLGHQTEFSSGS----------------GGAFTALIQ
DereGG19698       387 SRPSSSSPT--------------------------------------------------------------QQHVLLKLNT
DmojGI15491       418 SRPSSSSPT--PTPTS-------------------------------------------------------MPHVLLKLNT
DereGG18309       422 AAPVSVGVGGNGATMG--MGMGMGVGMGMGMNHYG---GGYDSANSLEAGQYAA---------------HLPTVLPEMHG
DgriGH11831*      285 ------------------------------------------------------------------------AKYLELHS
DperGL16499       442 AAPVTNGAGGMGVGVGVGMGVGMGMGVGMSMNHYG---SGYETASSLDAGNYA----------------HLPAVLPEMHG
DwilGK19815       382 AAAAMAMPQ------------------------------------------------------------------FPFN-
DereGG17578       403 RYHTNASN-EYNVHSSQN----------GPRSLSDSSQAESPVQEDLLTTNTPNLGS--------------TAGGGAANG
DmojGI11519       343 --------------------------------------------------------------------------------
DpseGA25684       315 RYHTNASN-DYNVHSSQN----------GPRSLSDSSQAESPVQEDLLSTNTPNLGSG-------------TGGGGGAAN
DanaGF15967       445 DEE----------ETDSIS---------REENTN----HNNGMAGSLG-ESSPGS----------------GGAFTALIQ
DsiGD17495        421 DEE--EAEAEAEAEAEAEA---------EEAEVGGLSLNGGGIQGTLHSESSPGS----------------GGAFTALIQ
DgriGH17737/38*   414 DDEESQPNLDPEAETDAEA--------EAGRSRSPTLLSADASSVATPTEPAPGS----------------GGAFTALIQ
DseGM22478        421 AAPVSVGVGGNGATMG--MGMGVGV--GMGMNHYG---GGYDSANSLEAGQYAA---------------HLPAVLPEMHG
DanaGF21771       420 AAPVSV--TGNGATMG--VGMGMGVGVGMGMGAMNP-YGGYDATNSLEAGNYAA---------------HLPAVLPEMHG
DvirGJ18742       398 AA-----------------PVPVPVATASAMNHYSSAMGGYDTTT-LDAGNY-----------------HISSVLPDMH-
DseGM23050        383 SRPSSSSPT--------------------------------------------------------------QQHVLLKLNT
DanaGF15970       410 SRPSSSSPT--------------------------------------------------------------QQHVLLKLNT
DpseGA14956       416 SRPSSSSPT--------------------------------------------------------------QQHVLLKLNT
DperGL15927       381 RYHTNASN-DYNVHSSQN----------GPRSLSDSSQAESPVQEDLLSTNTPNLGSG-------------TGGGGGTAN
DwilGK20052/54*   420 RYHTNASNNDYNVHSSQN----------GPRSLSDSSQAESPVQEDLLSTNTPNLGG--------------SGTGAANGG
DseGM23044/45*    408 DEE--EAEAEAEAEAEAEA---------EEAEVGGLSLNGGGIQGTLHSESSPGS----------------GGAFTALIQ
DereGG19691       437 DEE--EAEAEAEAEAEAEA---------EEAEVGGLSLNGGGIQGPLHSESSPGS----------------GGAFTALIQ
DmeCG34145        397 RYHTNASN-EYNVHSSQN----------GPRSLSDSSQAESPVQEDLLTTNTPNLGS--------------TAGGGAANG
DmojGI15484/86*   375 DEEEPDP--------------------EAGRSRSPTLLSADASSVATLVEPAPGAGSGALDGTGTSGSGGGGGAFTALIQ
DsiGD17500        383 SRPSSSSPT--------------------------------------------------------------QQHVLLKLNT
DyakGE17894       393 SRPSSSSPT--------------------------------------------------------------QQHVLLKLNT
Dmelz             425 AAPVSVGVGGNGATMG--MGMGVGV--GMGMNHYG---GGYDSANSLEAGQYAA---------------HLPAVLPEMHG
DsiGD16951*           --------------------------------------------------------------------------------
DgriGH17740       401 SRPSSSSPTPMPTPTA-------------------------------------------------------TPHVLLKLNT
DmojGI14398       420 AAPV---------------PVPVPVGTASAMNHYSSAMSGYETTT-LDAANY-----------------HISTVLPEMH-
```

```
DmeRun              402 SIETSSIHEQSASDGDSDDE------------------------------------QIDVVKSEFDLDKSLD--------
DvirGJ19252         418 SIETSSIHEQSASDADSDDE------------------------------------QIDVVKSEYELDKSIDSTRSSP--
DanaGF15983         464 AANAGGSGGAAGTGSSSGA----------------GGAAIPAGNP-SMLGANQNFPGIVNQAQHAS-ASAYGGGAGVGVG
DvirGJ18917*        447 GGAANGAASAAGAGSGASA-----------------GASAGAGNP-AMLGANQNFPGIVNQAQHAS-ASAYGGGVGVG--
DseGM22662/63*      451 GAGSNAGSGAAGSGAGGAG-----------------VASSAVGNP-AMLGANQNFPGIVNQAQHSA-ASAYGG--GTGV-
DpseGA13742/42*     505 RS-KNPSELFGGFAAAGAN---------------HFAP-SHSFNPALAAQLFLQSP-LLPQSSQWLYTQLYGSYSD-LPW
DyakGE17890         481 RSGKNPTELFGGFAGGAGGN--------------HFAHSGHSFNPALAAQLFLQSP-LLPQSSQWLYTQLYGSYSD-LPW
DvirGJ19245/46*     412 RS-KNPSELFGGFAAAGAG---------------HFAPAAHSFNPALAAQLFLQSP-LLPQSSQWLYTQLYGSYSD-LPW
DyakGE17788         489 HGFATDPYQTAG-YGGGN---------------TAGGS-------ASKSELDYGGGYNQAWSNGYQNY-QYGSCLATA--
DpseGA22609         501 HGFATDPYQTAGGYGTGAGA--------------GKSDLENLGGGGYGAG---GYNNAAAWSNGYNNY-QYGNCSAS--A
DwilGK25373         462 HGIATDPYQMAGGYGSASGNPG-----------KTTTELETLNVTGYGSGAGSGYNN--AWSNGYNNYPQYGSCSAVATG
DperGL15806         438 SIETSSIHEQSASDADSDDE------------------------------------QIDVVKSEYDLDKSIDSSRSSP--
DyakGE15340/41*     467 GAGSNAGSGAAGSGAGGAG-----------------GASSAVGNP-SMLGANQNFPGIVNQAQHSA-ASAYGG--GTGV-
DgriGH17774/75*     438 GGAANGSGSAAVAGSVASS-----------------VASAGAGNP-GMLGTNQNFPGIVNQAQHAS-ASAYGGGVGVG--
DsimGD15530*        448 GAGSNAGSGAAGSGAGGAG-----------------GASSAVGNP-KGLGANQNFPGM----------------------
DmeCG42267          480 RSGKNPTELFGGFAA-AGGN--------------HFAPSGHSFNPALAAQLFLQSP-LLPQSSQWLYTQLYGSYSD-LPW
DperGL15800/02*     470 --------------------------------------------------LFMQSP-LLPQSSQWLYTQLYGSYSD-LPW
DwilGK19810/11*     408 RS-KNHTDLFGGFSANGSASGAAGGGGGAGNPFPSSTPHTPTFNPALAAQLFLQSPALLPQSSQWLYSQLYGSYTDHLPW
DereGG19698         406 SIETSSIHEQSASDGDSDDE------------------------------------QIDVVKSEFDLDKSLD--------
DmojGI15491         442 SIETSSIHEQSASDADSDDE------------------------------------QIDVVKSEYELDKSIDSTRSSP--
DereGG18309         482 HGFATDPYQTAG-YGGGN---------------AAGGS-------ASKSELDYGGGYNQAWSNGYQNY-QYGSCLATA--
DgriGH11831*        293 HSLLPKLVQAST--------------------------------------------------------------------
DperGL16499         503 HGFATDPYQTAGGYGTGAGA--------------GKSDLENLGGGGYGAG---GYNNAAAWSNGYNNY-QYGNCSAS--A
DwilGK19815         395 ------------HDADSDDE------------------------------------QIDVVKSEYDLDKSIDSTRSSP--
DereGG17578         458 GAGTNAGSGAAGSGAGGAG-----------------GAASAVGNP-SMLGANQNFPGIVNQAQHSA-ASAYGG--GTGV-
DmojGI11519         343 ------------------------------------------------AGRQRNFPGIVNQAQHAS-ASAYGG--GVGVG
DpseGA25684         371 GGAANGGAGAAGAGAGSASSA-------------GAAAAVGVGNPGAMLGANQNFPGIVNQAQHAS-ASAYGG--GAG--
DanaGF15967         485 RS-KNPTDLFGGFAGAGSGN--------------HFPP-AHSFNPALAAQLFLQSP-LLPQSSQWLYSQLYGSYSD-LPW
DsiGD17495          474 RSGKNPTELFGGFAG-AGGN--------------HFAPSGHSFNPAQAAQLFLQSP-LLPQSSQWLYTQLYGSYSD-LPW
DgriGH17737/38*     470 RS-KNPTELFGGFAAAGAAS--------------HFAPAAHSFNPALAAQLFLQSP-LLPQSSQWLYTQLYGSYSD-LPW
DseGM22478          479 HGFATDPYQTAG-YGGGN---------------TGGGS-------ASKSELDYGGSYNQAWSNGYQNY-QYGSCLATA--
DanaGF21771         480 HGFATDPYQTAG-YATGTGGVA-----------TGGGGNAVGNAGGSKSELDYGGGYNQAWSNGYQNY-QYGSCSATS--
DvirGJ18742         442 -GFSADPYQTAAASGYCGTS--------------SKSDLESIN-ASYGATP-AAYNNPAAWSNGYNNY-QYGSCSATA-A
DseGM23050          402 SIETSSIHEQSASDGDSDDE------------------------------------QIDVVKSEFDLDKSLD--------
DanaGF15970         429 SIETSSIHEQSASDADSDDE------------------------------------QIDVVKSEYDLDKSIDSTRSSPP-
DpseGA14956         435 SIETSSIHEQSASDADSDDE------------------------------------QIDVVKSEYDLDKSIDSSRSSP--
DperGL15927         437 GGAANGGAGAAGGGAGSASSA-------------GAAAAVGVGNPGAMLGANQNFPGIVNQAQHAS-ASAYGG--GAG--
DwilGK20052/54*     476 SGATGGGTAGTGSGISSPG----------------SGAVGAGGVASMLVGSNQNFQAHVSQHVSASSAYVSGGGGGVGGG
DseGM23044/45*      461 RSGKNPTELFGGFAG-AGGN--------------HFAPSGHSFNPALAAQLFLQSP-LLPQSSQWLYTQLYGSYSD-LPW
DereGG19691         490 RSGKNPTELFGGFAG-AGGN--------------HFAPSGHSFNPALAAQLFLQSP-LLPQSSQWLYTQLYGSYSD-LPW
DmeCG34145          452 GAGSNAGSGAAGSGAGGAG-----------------GASSAVGNP-AMLGANQNFPGIVNQAQHSA-ASAYGG--GTGV-
DmojGI15484/86*     435 RS-KNPSELFGGFAAAGGG---------------HFAPTAHSFNPALAAQLFLQSP-LLPQSSQWLYTQLYGSYSE-LPW
DsiGD17500          402 SIETSSIHEQSASDGDSDDE------------------------------------QIDVVKSEFDLDKSLD--------
DyakGE17894         412 SIETSSIHEQSASDGDSDDE------------------------------------QIDVVKSEFDLDKSLD--------
Dmelz               483 HGFATDPYQTAG-YGGGN---------------TGGGS-------ASKSELDYGGSYNQAWSNGYQNY-QYGSCLATA--
DsiGD16951*             --------------------------------------------------------------------------------
DgriGH17740         427 SIETSSIHEQSASDADSDDE------------------------------------QIDVVKSEYELDKSIDSTRSSP--
DmojGI14398         466 -GFSADPYQTAAASGYCGTS--------------SKSDLETLN-ASYGATP-TAYNNAAAWSNGYNNY-QYGSCSATA-A
```

```
DmeRun            438 -------------------------------------VAPLRMRCDLKAPSAMKPLYHES---------GPGAVANSRQP
DvirGJ19252       460 ---------------------------------TQQISVPLRMRCDLKAPSALKPLYHET-------ASAAAAVAAPRQA
DanaGF15983       526 VGVGVGGGHGS-AAAAAAG----------CNGSLYPVLPASLLYSQLYTAANQSAHGFHSHTLP-AHASPNSSVHG-ELQ
DvirGJ18917*      506 ----VGGGHGGGAGATAAG----------CNGSLYPAYLT----------------------------------------
DseGM22662/63*    509 ----VGGGHGN-AAAAAAG----------CNGSLYPVLPASLLYSQLYTAANQSAHGFHSHTLP-AHASPNSSVHG-ELQ
DpseGA13742/42*   566 LRSAAAAAAASAVPAQVQAQAQTQAQPQ---DSATSLMSSGGDHDGVN--LIKRCVTLITH---NPPDAENANPNS--SP
DyakGE17890       545 LRNAAAAAAAN-INPGQENAG-------------VPPLGSNPDHDGVN--LIKRCVTLITHXXXNPPDAENANPNA--SP
DvirGJ19245/46*   474 LRSAAAAAAANTAPQQHQQQQQESG-------SVLPAAAN--DADGVN--LIKRCVTLITH---NPPDAENANPNA--SP
DyakGE17788       543 QYGPQAAPPPQPPP--PPPVV--------LCPQLYSTVNQNQIHLHLHSSEKLEQYLGTATG---ADHLTIGSLTGSSRS
DpseGA22609       561 QYGGTGGHAAAPPPPPPPPVV--------LYPQLYSTVNQNQIHLHLHSSEKLEQYLGSASD----QQLTISSLTGS-RS
DwilGK25373       529 QYGGATAAAPPPP---PPPVV--------LYPQLYSTVNQNQIHLHLHSSEKLEQYLGTTAD----QQLTISSLTGN-RS
DperGL15806       480 --------------------------------LAQHISVPLRMRCDLKAPSALKPLFHES-----------AAAATNRQA
DyakGE15340/41*   525 ----VGGGHGS-AAAAAAG----------CNGSLYPVLPASLLYSQLYTAANQSAHGFHSHTLP-AHASPNSSVHG-ELQ
DgriGH17774/75*   497 ----VAGGHGGGAGATTAG----------CNGSLYPVLPASLLYSQLYTAANQSAHGFHSHTLP-AHASPNSSVHG-ELQ
DsimGD15530*          --------------------------------------------------------------------------------
DmeCG42267        543 LRNAAAAAAAN-INPGQENSG-------------IPPLGSNPDHDGVN--LIKRCVTLITH---NPPDAENANPNA--SP
DperGL15800/02*   498 LRSAAAAAAASAVPAQVQAQAQTQAQPQ---DSATSLMSSGGDHDGVN--LIKRCVTLITH---NPPDAENANPNS--SP
DwilGK19810/11*   487 LRSAAAAAAASSSSSASSSQQQPLTTTLTTVPAASTPAGASGGAVAVASDLIKRCVTLITH---NPPDVENSNPNAAASP
DereGG19698       442 -------------------------------------VAPLRMRCDLKAPSAMKPLYHES---------GPGAVASSRQP
DmojGI15491       484 ---------------------------------TQQISVPLRMRCDLKAPSALKPLYHESSAAA-AAAAAVAAVHAPRQA
DereGG18309       536 QYGPQAAPPPQPPP--PPPVV--------LCPQLYSTVNQNQIHLHLHSSEKLEQYLGTATG---AEHLTIGSLTGSSRS
DgriGH11831*      305 -----------------------------------VKQFKEDLQLAQHYEASFRMGLATAERR--PELLPMSMAIAN---
DperGL16499       563 QYGGTGGHSAAPPPPPPPPVV--------LYPQLYSTVNQNQIHLHLHSSEKLEQYLGSASD----QQLTISSLTGS-RS
DwilGK19815       425 ---------------------------------TQQISMPLRMRCDLKAPSSLKPLLHES--------TSTSPTAGLLQT
DereGG17578       516 ----VGGGHGS-AAAAAAG----------CNGSLYPVLPASLLYSQLYTAANQSAHGFHSHTLP-AHASPNSSVHG-ELQ
DmojGI11519       372 VGVGVGGGHGSGAGATAAG----------CNGSLYPVLPASLLYSQLYTAANQSAHGFHSHTLP-AHASPNSSVHG-ELQ
DpseGA25684       433 ----VGGGHGS-AAAAAAG----------CNGSLYPVLPASLLYSQLYTAANQSAHGFHSHTLP-AHASPNSSVHG-ELQ
DanaGF15967       547 LRNAAAAAAASNLNPNTTPTP-------------EGVPPSSQDHDGVN--LIKRCVTLITH---NPPDAENANPNA--SP
DsiGD17495        537 LRNAAAAAAAN-INPGQENPG-------------VPTLGSNPDHDGVN--LIKRCVTLITH---NPPDAENANPNA--SP
DgriGH17737/38*   533 LRSAAAAAAASTTPQQQQQQQQQQQQQQQLQDSVVPLVASG-DADGVN--LVKRCVTLITH---NPPDAENANPNA--SP
DseGM22478        533 QYGPQAAPPPQPPP--PPPVV--------LCPQLYSTVNQNQIHLHLHSSEKLEQYLGTATS---ADHLTIGSLTGSSRS
DanaGF21771       545 QYGPQTAAAPPPPP--PPPVV--------LYPQLYSTVNQNQIHLHLHSSEKLEQYLGTAAGG--GEQLTISSLTGSSRS
DvirGJ18742       503 QYGGAGAHATAP----PPPVV--------LYPQLYSTVNQNQIHLHLHSSEKLEQYL--ATD----QQLTISSLASN-RS
DseGM23050        438 -------------------------------------VAPLRMRCDLKAPSAMKPLYHES---------GPGAVANSRQP
DanaGF15970       472 --------------------------------VAHHHLGPLRMRCDLKAPSAMKPLYHEP---------SVVAVN-PRQA
DpseGA14956       477 --------------------------------LAQHISVPLRMRCDLKAPSALKPLFHES-----------AAAATNRQA
DperGL15927       499 ----VGGGHGS-AAAAAAG----------CNGSLYPVLPASLLYSQLYTAANQSAHGFHSHTLP-AHASPNSSVHG-ELQ
DwilGK20052/54*   540 GGGGGGGGGGGGGGSGAGGGNVQHGSTN-GGGSLYPVLPASLLYSQLYTAANQSAHGFHSHTLAAAAHSPNSSVHGAELQ
DseGM23044/45*    524 LRNAAAAAAAN-INPGQENPG-------------VPPVGSNPDHDGVN--LIKRCVTLITH---NPPDAENANPNA--SP
DereGG19691       553 LRNAAAAAAAN-INPGQENHG-------------APPLGSNPDHDGVN--LIKRCVTLITH---NPPDAENANPNA--SP
DmeCG34145        510 ----VGGGHGS-AAAAAAG----------CNGSLYPVLPASLLYSQLYTAANQSAHGFHSHTLP-AHASPNSSVHG-ELQ
DmojGI15484/86*   497 LRSAAAAAAASSTAAPQQQPQQQQSLPQQHEPSMLAAAAAGADADGVN--LIKRCVTLITH---NPPDAENANPNA--SP
DsiGD17500        438 -------------------------------------VAPLRMRCDLKAPSAMKPLYHES---------GPGAVANSRQP
DyakGE17894       448 -------------------------------------VAPLRMRCDLKAPSAMKPLYHES---------GPGAVASSRQP
Dmelz             537 QYGPQAAPPPQPPP--PPPVV--------LCPQLYSTVNQNQIHLHLHSSEKLEQYLGTATS---ADHLTIGSLTGSSRS
DsiGD16951*           --------------------------------------------------------------------------------
DgriGH17740       469 ---------------------------------THHISVPLRMRCDLKAPSALKPLYHETSAAA-AAAAAAVAAVVPRQA
DmojGI14398       527 QYGGAAGHATAP----PPPVV--------LYPQLYSTVNQNQIHLHLHSSEKLEQYL--ATD----QQLTISSLASN-RS
```

```
DmeRun          472 SPETT-------------------------------------------------------------------------
DvirGJ19252     500 SPETTLP-----------------------------------------------------------------------
DanaGF15983     593 SVMDHISNVGV-----RQQHNIM---AGGGVTHPGDLTLIGNCGASVRNIEDGNS-----------------------
DvirGJ18917*        ------------------------------------------------------------------------------
DseGM22662/63*  572 SVMDHISNVGV-----RQQHNIM---AGGGVTHPGDLTLIGNCGASVRNIEDGNS-----------------------
DpseGA13742/42* 636 PVSSTRRSPSPS------------------PVETIDLDDVSTTSRSASGSASG-------------------------
DyakGE17890     607 PVSSTRRSPSP--------------------VETIDLDDVSTTSRSASGSSGL------------------------G
DvirGJ19245/46* 538 PVSSSRRSPSP--------------------VETIDLDDVSTTSRSASGSSG--------------------------
DyakGE17788     610 SIEIGQD-------------------QYHQQVHHAQQQQQQQQQQ--VHHPQQQQQQ--------QVESAGEVG----
DpseGA22609     628 SIEIGPAGGLLGLTGEQEQ-HQHQNQHQQQQQLQQQPLEQSSEASNQS-YHLSHHQQHPHQ------QQQQQQQQQ----
DwilGK25373     593 SIEIGLGGGGGTVGGGVGG---AGALTVSSQDQDQQVAVSNEAVANQS-YHLASQEVQ---------QQQQQEVG----
DperGL15806     517 SPETTLP-----------------------------------------------------------------------
DyakGE15340/41* 588 SVMDHISNVGV-----RQQHNIM---AGGGVTHPGDLTLIGNCGASVRNIEDGNS-----------------------
DgriGH17774/75* 561 SVMDHISNVGV-----RQQHNIM---AGGGVAHPGDLTLIGNCGASVRNIEDGNT-----------------------
DsimGD15530*        ------------------------------------------------------------------------------
DmeCG42267      602 PVSSTRRSPSP--------------------VETIDLDDVSTTSRSASGSS--------------------------G
DperGL15800/02* 568 PVSSTRRSPSPS------------------PVETIDLDDVSTTSRSASGSASG-------------------------
DwilGK19810/11* 564 PVSSTRRSPSP--------------------VETIDLDDVSTTSRSASVSGSS-------------------------
DereGG19698     476 SPETT-------------------------------------------------------------------------
DmojGI15491     530 SPETTLP-----------------------------------------------------------------------
DereGG18309     603 SIEIGQD-------------------QYHQQVHHSQQQQQQQQQ---VHHPQQQQQQ--------QVESAGEVG----
DgriGH11831*        ------------------------------------------------------------------------------
DperGL16499     630 SIEIGPAGGLLGLTGEQEH-HQHQ-QQQQQQHLQQQPLEQSSEASNQS-YHLSHHQQHPHQ------QQQQQQQQQQQ--
DwilGK19815     464 SPETTSLT----------------------------------------------------------------------
DereGG17578     579 SVMDHISNVGV-----RQQHNIM---AGGGVTHPGDLTLIGNCGASVRNIEDGNS-----------------------
DmojGI11519     440 SVMDHISNVGV-----RQQHNIM---AGGGVAHPGDLTLIGNCGASVRNIEDGNT-----------------------
DpseGA25684     496 SVMDHISNVGV-----RQQHNIM---AGGGVTHPGDLTLIGNCGASVRNIEDGNS-----------------------
DanaGF15967     607 PVSSSRRSPSP--------------------VETIDLDDVSTTSRSASGASSGEAGGGASG----------------G
DsiGD17495      596 PVSSTRRSPSP--------------------VETIDLDDVSTTSRSASGSS--------------------------G
DgriGH17737/38* 605 PVSSTRRSPSP--------------------VETIDLDDVSTTSRSASGSSG--------------------------
DseGM22478      600 SIEIGQD-------------------QYHQQVHHAQQQQQQQ-----VHHPQQQQ-----------VESAGEVG----
DanaGF21771     613 SIEIAQD-------------------PYQ---HHQQQHHQHP-------HPQQTEQQ---------VVPGGGTG----
DvirGJ18742     564 SIEIGLGTGVCGGGGGGSSVSGGVALGEQEQQAAVSIAEQAAEGASQN-YHLHHHHH---------ETQRQMEAQ----
DseGM23050      472 SPETT-------------------------------------------------------------------------
DanaGF15970     510 SPETTIP-----------------------------------------------------------------------
DpseGA14956     514 SPETTLP-----------------------------------------------------------------------
DperGL15927     562 SVMDHISNVGV-----RQQHNIM---AGGGVTHPGDLTLIGNCGASVRNIEDGNS-----------------------
DwilGK20052/54* 619 SVMDHISNVGVGVVRHQQHHHNIMSAAGGGLTHPGDLTLIGNCGASVRNIEDGNVT----------------------
DseGM23044/45*  583 PVSSTRRSPSP--------------------VETIDLDDVSTTSRSASGSS--------------------------G
DereGG19691     612 PVSSTRRSPSP--------------------VETIDLDDVSTTSRSASGSS--------------------------G
DmeCG34145      573 SVMDHISNVGV-----RQQHNIM---AGGGVTHPGDLTLIGNCGASVRNIEDGNS-----------------------
DmojGI15484/86* 570 PVSSSRRSPSP--------------------VETIDLDDVSTTSRSASGSSG--------------------------
DsiGD17500      472 SPETT-------------------------------------------------------------------------
DyakGE17894     482 SPETT-------------------------------------------------------------------------
Dmelz           604 SIEIGQD-------------------QYHQQVHHAQQQQQQQQQQQQVHHPQQQQ-----------VESAGEVG----
DsiGD16951*         ------------------------------------------------------------------------------
DgriGH17740     515 SPETTLP-----------------------------------------------------------------------
DmojGI14398     588 SIEIGLGTGLSGG----------VSLGEQDQQAAVAIAEQAAEGASQNNYHLHHHHQQQQQQQQLEAQRQLEAQHQQQ
```

```
DmeRun              477 ------------------TKIKSAAVQQKTVWRPY
DvirGJ19252         507 ----------------AATKLKNAVVQQKTVWRPY
DanaGF15983         640 --------NRQVAALAAHRGHHNPTDNGGSVWRPY
DvirGJ18917*            -----------------------------------
DseGM22662/63*      619 --------NRQVAALAAHRGHHNPTDNGGSVWRPY
DpseGA13742/42*     671 -----------SGSSGGGPIRTRTPKPSADVWRPY
DyakGE17890         641 LGGGVGGMGGVGGAVG--PIRTRTPKPSADVWRPY
DvirGJ19245/46*     570 ---------------AGGPIRTRTPKPTADVWRPY
DyakGE17788         655 -------GSVAGGG--VESAREEDVGDLTQVWRPY
DpseGA22609         696 ----AVEQVQQGEGVGVEAPRDEDVGDLTQVWRPY
DwilGK25373         655 ---------------GEVSTRQED--DLTQVWRPY
DperGL15806         524 ----------------AATKLKNSAVQQKTVWRPY
DyakGE15340/41*     635 --------NRQVAALAAHRGHHNPTDNGGSVWRPY
DgriGH17774/75*     608 --------NRQVAALAAHRGHHNPTDNGGSVWRPY
DsimGD15530*            -----------------------------------
DmeCG42267          634 HGGGVG----GGGAVG--PIRTRTPKPSADVWRPY
DperGL15800/02*     603 -----------SGSSGGGPIRTRTPKPSADVWRPY
DwilGK19810/11*     597 ---------GGGPGSGGGPIRTRTPKPTADVWRPY
DereGG19698         481 ------------------TKIKSAAVQQKTVWRPY
DmojGI15491         537 ----------------AATKLKNAVVQQKTVWRPY
DereGG18309         647 -------GSGAGG---VESAREEDVGDLTQVWRPY
DgriGH11831*            -----------------------------------
DperGL16499         699 ----AVEQVQQGEGVGVEAPRDEDVGDLTQVWRPY
DwilGK19815         472 ---------------TAATKLKSAAVQQKTVWRPY
DereGG17578         626 --------NRQVAALAAHRGHHNPTDNGGSVWRPY
DmojGI11519         487 --------NRQVAALAAHRGHHNPTDNGGSVWRPY
DpseGA25684         543 --------NRQVAALAAHRGHHNPTDNGGSVWRPY
DanaGF15967         649 LAGGVGGAAAAAAVSGGGPIRNRTPKPSADVWRPY
DsiGD17495          628 HGGGVG----VGGAVG--PIRTRTPKPSADVWRPY
DgriGH17737/38*     637 ---------------AGGPIRTRTPKPTSDVWRPY
DseGM22478          639 -------GSGAGG---VESAREEDVGDLSQVWRPY
DanaGF21771         651 -------GGGGGSGDVVESPREEDVGDLTPVWRPY
DvirGJ18742         629 --------QQHQSDVSNEAAREEDVGDMTQVWRPY
DseGM23050          477 ------------------TKIKSAAVQQKTVWRPY
DanaGF15970         517 ----------------AATKLKSASVQQKTVWRPY
DpseGA14956         521 ----------------AATKLKNSAVQQKTVWRPY
DperGL15927         609 --------NRQVAALAAHRGHHNPTDNGGSVWRPY
DwilGK20052/54*     676 --------NRQVAALAAHRGHHNPTDNGGSVWRPY
DseGM23044/45*      615 HGGGVG----VGGAVG--PIRTRTPKPSADVWRPY
DereGG19691         644 HGGGVG------GAVG--PIRTRTPKPSADVWRPY
DmeCG34145          620 --------NRQVAALAAHRGHHNPTDNGGSVWRPY
DmojGI15484/86*     602 ---------------AGGPIRTRTPKPTADVWRPY
DsiGD17500          477 ------------------TKIKSAAVQQKTVWRPY
DyakGE17894         487 ------------------TKIKSAAVQQKTVWRPY
Dmelz               648 -------GSGAGG---VESAREEDVGDLSQVWRPY
DsiGD16951*             -----------------------------------
DgriGH17740         522 ----------------AATKLKNAVVQQKTVWRPY
DmojGI14398         658 QQQQHQQQQQHPSDVSNEAAREEDVGDMTQVWRPY
```
