# Supplementary material for: Evolutionary origin and genomic organisation of runt-domain containing genes in arthropods
Source: BMC Genomics. 2008 Nov 25;9:558. doi: 10.1186/1471-2164-9-558 (PMC2631020; doi:10.1186/1471-2164-9-558)
Supplement: Additional file 2 — Phylogeny and multiple sequence alignment of endopterygotan insects full-length RD protein sequences. ClustalX alignment and Bayesian phylogeny of full-length RD protein sequences from a six endopterygotan insects: Drosophila melanogaster, Aedes. aegypti, Tribolium castaneum, Nasonia vitripennis, Apis mellifera and Bombyx mori. Alignment includes the outgroup Strongylocentrotus purpuratus. [file 1471-2164-9-558-S2.pdf]

Additional File A2: Phylogeny and multiple sequence alignment of endopterygotan insects full length RD protein sequences.

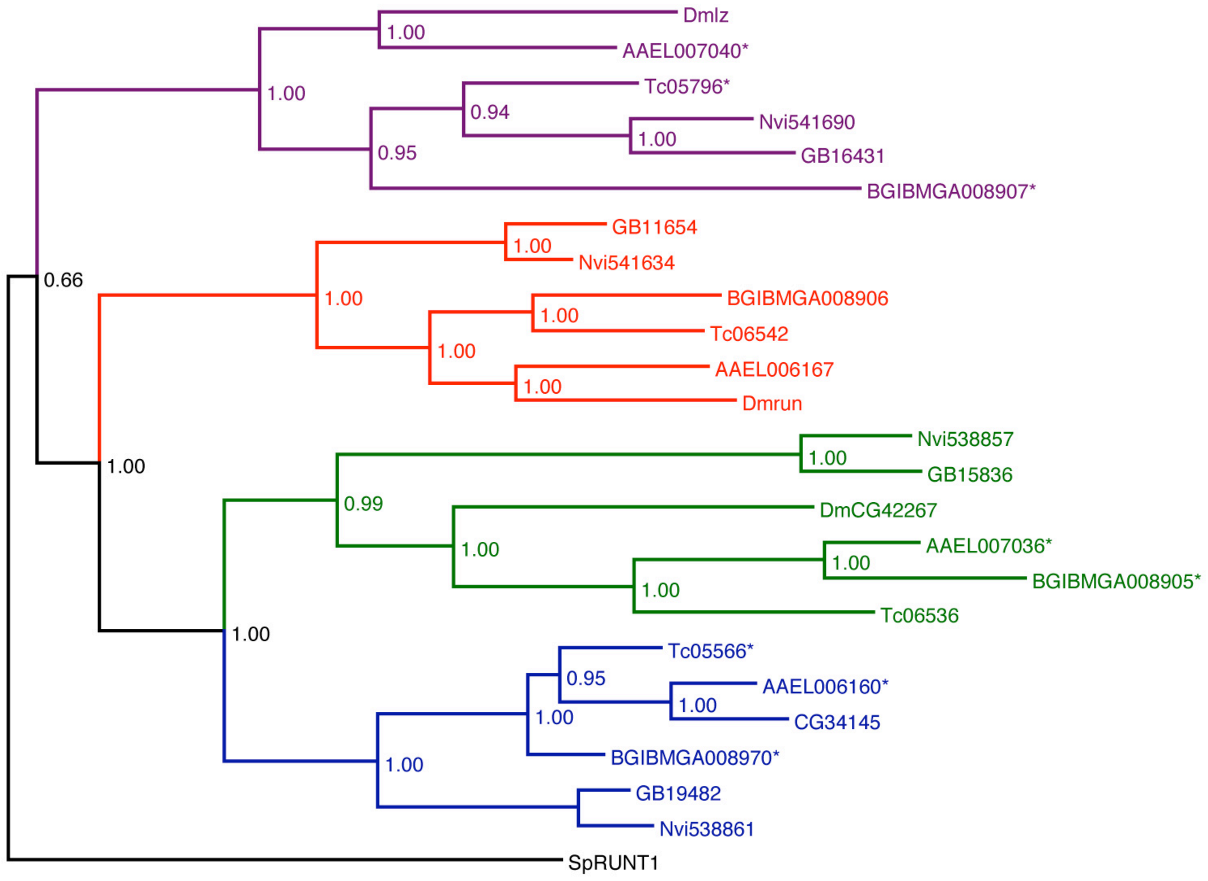

```
GB19482             1 ------------------------------------------------------------
Nvi538857           1 ------------------------------------------------------------
CG42267             1 ------------------------------------------------------------
GB11654             1 ------------------------------------------------------------
Dmlz                1 MHLHLLAAEKTPPSPSPNPTPTPSASPSGHTGAAGESQLDLASQTESQLQLGLPLASGYG
Tc05796*            1 MHL---------PVS-----LSSCTVS---------------------------------
AAEL007040*         1 MHL---------PSGGPTAIIPPISNNEANVTPTASTEWDIYKDEE--------------
BGIBMGA008907*      1 ------------------------------------------------------------
AAEL007036*         1 ------------------------------------------------------------
BGIBMGA008906       1 ------------------------------------------------------------
BGIBMGA008905*      1 ------------------------------------------------------------
Tc05566*            1 ------------------------------------------------------------
AAEL006160*         1 ------------------------------------------------------------
Nvi538861           1 ------------------------------------------------------------
Tc06536             1 ------------------------------------------------------------
AAEL006167          1 ------------------------------------------------------------
Nvi541634           1 ------------------------------------------------------------
Nvi541690           1 MHL---------PVG-----SVSCSGESFGSLSQQHHHHQHHQQHHQHHQQHHQLQ----
BGIBMGA008970*      1 ------------------------------------------------------------
GB15836             1 ------------------------------------------------------------
GB16431             1 MKS---------AVPRPPRITTDCRGSLWAIYGDGDDRQADIRKPDSGGSSATLLEYPGH
SpRUNT1             1 ------------------------------------------------------------
Tc06542             1 ------------------------------------------------------------
Dmrun               1 ------------------------------------------------------------
CG34145             1 ------------------------------------------------------------


GB19482             1 ------------------------------------------------------------
Nvi538857           1 ------------------------------------------------------------
CG42267             1 ------------------------------------------------------------
GB11654             1 ------------------------------------------------------------
Dmlz               61 LGLGLGLGLGLGLGQELVADHSTTVAPVSVAGPGRLGRSINGSGGSHHHHHLHHHYSPYH
Tc05796*           14 ------------------------------------------------------------
AAEL007040*        38 ------------------------------------------------------------
BGIBMGA008907*      1 ------------------------------------------------------------
AAEL007036*         1 ------------------------------------------------------------
BGIBMGA008906       1 ------------------------------------------------------------
BGIBMGA008905*      1 ------------------------------------------------------------
Tc05566*            1 ------------------------------------------------------------
AAEL006160*         1 ------------------------------------------------------------
Nvi538861           1 ------------------------------------------------------------
Tc06536             1 ------------------------------------------------------------
AAEL006167          1 ------------------------------------------------------------
Nvi541634           1 ------------------------------------------------------------
Nvi541690          43 ---------QEQDNGNLVALQP--------------------------------------
BGIBMGA008970*      1 ------------------------------------------------------------
GB15836             1 ------------------------------------------------------------
GB16431            52 PSCEPEAEKQLLEKKLLVAAIAE-------------------------------------
SpRUNT1             1 ------------------------------------------------------------
Tc06542             1 ------------------------------------------------------------
Dmrun               1 ------------------------------------------------------------
CG34145             1 ------------------------------------------------------------
```

```
GB19482              1 ------------------MDIFGRCNGGGGG--------------SSTVSGSG--AGSVR
Nvi538857            1 ---------MTNPARDARGGVAVENYAGGGSDPSSRLQQSHHQQHLPHHSSHHHHHHHHH
CG42267              1 ----------------------MHISAEVSSTTSNQIQQQQQQHQQQQQHQLLQHQQQQT
GB11654              1 ------------------------------------------------------------
Dmlz               121 HAHPYHPPHPHAPHHHHHHHPPYPYPPAGPHPPAMVTSSSTSPTGNGWSSSTGDFKGITA
Tc05796*            14 -------------------------EEAWPKSSGMCEP----------------------
AAEL007040*         38 ----------------------MLEPPAG-------------------------------
BGIBMGA008907*       1 ------------------------------------------------------------
AAEL007036*          1 ----------------------MHLAT----------------------------PEVTS
BGIBMGA008906        1 ------------------------------------------------------------
BGIBMGA008905*       1 ----------------------MHLAS-----------------------------EVSS
Tc05566*             1 ------------------------------------------------------------
AAEL006160*          1 ------------------------------------------------------------
Nvi538861            1 ------------------MDIFGRCNGGAGLGVNSVPATAAVVAQSATTRSANNLAGPVV
Tc06536              1 ----------------------MGQSESCG-----------------------PPPQHHS
AAEL006167           1 ------------------------------------------------------------
Nvi541634            1 ------------------------------------------------------------
Nvi541690           56 ------------------AQYQQQIQEQHQQQQQQVEG----------------------
BGIBMGA008970*       1 ------------------------------------------------------------
GB15836              1 ---------MTNPLRDAR----PEDRPLGLDYPDPRLVRNGHQEHQGNTTAHQ-------
GB16431             75 -----------------ESPWSVQIEEASEITTSCPEGTCLGDEGRQLADGMHLPVGSVG
SpRUNT1              1 ------------------------------------------------------------
Tc06542              1 ------------------------------------------------------------
Dmrun                1 --------------------------MHLPAGPTMVANNTQVLAAAAAAAAAAAAAVAQG
CG34145              1 -------------------------MEHEGANSNNYKMHLTTTSSNSTASNANNNNNNTA

GB19482             27 GSGNESSCGTASG-----------ASESGGTSDRMQLTGASAPGVAASPESGVSP-----
Nvi538857           52 QQQQHQHRHQQDQQHSSSGSNRLRRSRTSSLRRNGSRTMHLSGETQAQQQAAAPA-----
CG42267             39 ATTTTTKRRNAESSASSNNNNNNNTSTTNNNNTNNNNNSTTNNNNNNNNNNVKTK-----
GB11654              1 ----------------------------------------------------MHL-----
Dmlz               181 VATATGGGVGGATQGATASTGATAAEVLAVSSSASVGSSSPTGGASNGTAHSGHSGHTGG
Tc05796*            27 -----------------------------------------SSGYVS-------------
AAEL007040*         45 -------------------------------ANNNAENSSPSPGGSG-------------
BGIBMGA008907*       1 ----------------------------------MRFGLKGGAAVVT-------------
AAEL007036*         11 ---TTNPTSMELNGNSGGGLNTDSVNT---NGTTNDGTSKHQP-----------------
BGIBMGA008906        1 ----------------------------------------------------MHL-----
BGIBMGA008905*      10 --------------------TTNGLGHEAPSSSYNGGSLPSQP-----------------
Tc05566*             1 ------------------------------------------------------------
AAEL006160*          1 ------------------------------------------------------------
Nvi538861           43 GPEHASTVGNTGGGGNGNNNGPAAPAEAATSQSRMQLTGASAPGNAASPESGVSP-----
Tc06536             16 RDFSSHFRAVRWPRHAAKKLTSMHLATGEVTSTTGHYDRSYGN-----------------
AAEL006167           1 ------------------------------------------------------------
Nvi541634            1 ----------------------------------------------------MHL-----
Nvi541690           76 ----------------------------QRQEEAAEPGPTGAAGEMS-------------
BGIBMGA008970*       1 ------------------------------------MHLTGANSGAVSPDTSSTL-----
GB15836             41 -----------------IGAARIRRTRTSSTRRR----MHLANEVPPQGSTGG-------
GB16431            118 CGENYATVGSHEGAGPCGPSLAPLQGVNTRYQQADDADAGGGGGEMS-------------
SpRUNT1              1 -------------MHITDVNVDHLLSSTAPLANHPSKDPVRRNNLHNSYKMAEGG-----
Tc06542              1 ----------------------------------------------------MHL-----
Dmrun               35 PGPQQSSNATTASAIAINPAQSLANTSTHSASSTGSSTPDLSTNNTSSSSNATTS-----
CG34145             36 NNNNTSSNNNTTN--------TNGSSNNNTSGNNNNSSGNNSNTEQNTPPTPAQL-----
```

```
GB19482           71 ------------------LTDAYTKMTSDILAERTLGDFVSEHPGELVRTGSPHLVCTVL
Nvi538857        107 ------------------SQLPDYALTADLLAERTLDGLLAEHPGELVRTGSPHVVCTVL
CG42267           94 ------------------PVDTSPYLTPENLIERTVDVLLAEHPGELVKTGSPHVVCTTL
GB11654            4 ------------------PEG----PLGMD--INAMHETLQACHGDLVRTGSPAILCSAL
Dmlz             241 HSSSTASNNNNNGASNSNSNNNNAVHQDLLWMERLVQKRQQEHPGELVRTSNPYFLCSAL
Tc05796*          33 ---------------------NNQ---DIWWTEHLIHEIQTEHPGELVRTGSPYFLCSAL
AAEL007040*       61 ---------------------------DLWWIERMVMEAQQEYPGELVRTGSPYFLCSAL
BGIBMGA008907*    14 ------------------------ILETLELISQGEWRIGGLTFLVAVKTGSPDYMCSIL
AAEL007036*       48 -------------------------LTAESLAERTIEGLLADHPGELIRTGSPHVVCTVL
BGIBMGA008906      4 ------------------PHASPAV--RMADVYAHIHEYYRQSHGELVQTGSPAVLCSAL
BGIBMGA008905*    33 -------------------------LTAELLAERTLEGLLADHPGELVKTGSPH-VCTVL
Tc05566*           1 -------------------------MTSDILAERTLNDFLSEHPGELIRTGSPLFVCTVL
AAEL006160*        1 -------------------------MTSDILAERTLGDFLSEHPGELIRTGSPLFVCTVL
Nvi538861         98 ------------------LADAYTKMTSDIFAERTLGDYMSEHPGELVRTGSPHLVCTVL
Tc06536           59 -------------------------LTAEMLAERTIDGLLAEHPGELVRTGSPHVVCTVL
AAEL006167         1 ----------------------------MADMFSNLHDMLQEYHGELVQTGSPAVLCSAL
Nvi541634          4 ------------------PEG----PIGMENHFSAIHDALRACHGELVQTGSPAILCSPL
Nvi541690         95 ---------------------EAAAVGELWWTERLVGEAQAEHPGELVRTGSPYFLCSQL
BGIBMGA008970*    20 ------------------LHETYTKMTSDILAERTLGDFLSEHPGELVRTGSPHFVCTVL
GB15836           73 ------------------NNLPDYALTADLLTERTLDGLFAEHPGELVRTGSPHVVCTVL
GB16431          165 ---------------------EGAAVGELWWTERLVGEAQAEHPGELVRTGSPYFLCSQL
SpRUNT1           43 --------------------QRNKASSVFKGGERSIVDALSEYPGELVKTESPNFACSVL
Tc06542            4 ------------------PINAQQP--SIMDMYASLQETLQEYHGELVQTGSPAVLCSVL
Dmrun             90 ------------------PQNSAKMPSSMTDMFASLHEMLQEYHGELAQTGSPSILCSAL
CG34145           83 ------------------LNEAYTKMTSDILAERTLGDFLTEHPGELIRTSSPLFVCTVL

GB19482          113 PAHWRSNKTLPVAFKVVALGEVGDGTLVTVRAGNDENCCAELRNSTALMKNQVAKFNDLR
Nvi538857        149 PSHWRSNKTLPVAFKVVALGEVGDGTLVTVRAGNDENCCAELRNSTALMKNQVAKFNDLR
CG42267          136 PTHWRSNKTLPIAFKVLALGEVMDGTIVTIRAGNDENFCGELRNCTAVMKNQVAKFNDLR
GB11654           40 PSHWRSNKSLPVAFKVVALDDVSDGTLVTIRAGNDENCCGELRNCTAVMKNQVAKFNDLR
Dmlz             301 PAHWRSNKTLPMAFKVVALAEVGDGTYVTIRAGNDENCCAELRNFTTQMKNDVAKFNDLR
Tc05796*          69 PTHWRSNKTLPVAFKVVALGDIGDGTIVTVRAGNDENCCAELRNSTAVMKNQVAKFNDLR
AAEL007040*       94 PNHWRSNKTLPSAFKVVSLGDVCDGTMVTIRAGNDENFCGELRNCTAVMRNQVAKFNDLR
BGIBMGA008907*    50 PQHWRSNKTLPGGFKVVALGDVLDGTLVTVRAGNDENCSAELRNNSAVMKNRVAKFNDLR
AAEL007036*       83 PNHWRSNKTLPVAFKVVALGDVGDGTMVTVMAGNDENFCGELRNCTAIMKNQVAKFNDLR
BGIBMGA008906     44 PGHWRSNKSLPLAFKVVALDDVQDGTLVTIKAGNDENVMAELRNCTAVMKNQVAKFNDLR
BGIBMGA008905*    67 PPHWRSNKTLPVAFKVVALGDVGDGTLVTVRAGNDENCSAELRNCTAVMKNQVAKFNDLR
Tc05566*          36 PPHWRSNKTLPVAFKVVALGDVGDGTVVTVKAGNDENYCAELRNSTAVMKNQVAKFNDLR
AAEL006160*       36 PPHWRSNKTLPVAFKVVALGDVGDGTMVTVRAGNDENYCAELRNCTAVMKNQVAKFNDLR
Nvi538861        140 PAHWRSNKTLPVAFKVVALGEVGDGTVVTVRAGNDENCCAELRNSTALMKNQVAKFNDLR
Tc06536           94 PPHWRSNKTLPVAFKVVALGDVGDGTVVTVRAGNDENYCAELRNCTAVMKNQVAKFNDLR
AAEL006167        33 PTHWRSNKSLPCAFKVIALDDIQDGTVVTIKAGNDENCHAELRNCTAIMKNQVAKFNDLR
Nvi541634         42 PSHWRSNKSLPVAFKVVALDEVSDGTLVTISAGNDENFCGELRNCTAVMKNQVAKFNDLR
Nvi541690        134 PTHWRSNKTLPAAFKVVALGEIGDGTLVTVRAGNDENCCAELRNSTALMKNQVAKFNDLR
BGIBMGA008970*    62 PPHWRSNKTLPVAFKVVALGDIGDGTLVTVRAGNDENCCAELRNSSAVMKNQVAKFNDLR
GB15836          115 PAHWRSNKTLPVAFKVVALGEVGDGTLVTVRAGNDENCCAELRNSTAVMKNQVAKFNDLR
GB16431          204 PTHWRSNKTLPVAFKVVALGEVVDGTLVTVRAGNDENCCAELRNSTTLMKNQVAKFNDLR
SpRUNT1           83 PNHWRCNKSLPVAFKVVSLGETKDGTMVTIAAGNDENYCAELKNNTAVMKNQVARFNDLR
Tc06542           44 PSHWRSNKSLPIAFKVVALDEVRDGTTVTLKAGNDENYCAELRNCTAVMKNQVAKFNDLR
Dmrun            132 PNHWRSNKSLPGAFKVIALDDVPDGTLVSIKCGNDENYCGELRNCTTTMKNQVAKFNDLR
CG34145          125 PPHWRSNKTLPVAFKVVSLGDIMDGTMVTVRAGNDENYCAELRNCTAVMKNQVAKFNDLR
```

```
GB19482          173 FVGRSGR--------------------------------------------------GKS
Nvi538857        209 FVGRSGR--------------------------------------------------GKS
CG42267          196 FVGRSGR--------------------------------------------------GKS
GB11654          100 FVGRSGR--------------------------------------------------GKS
Dmlz             361 FVGRSGR--------------------------------------------------GKS
Tc05796*         129 FVGRSGR--------------------------------------------------GKS
AAEL007040*      154 FVGRSGR--------------------------------------------------GKS
BGIBMGA008907*   110 FVGRSGRSYVREIIQY--------------------------------------FVPGKS
AAEL007036*      143 FVGRSGR--------------------------------------------------GE-
BGIBMGA008906    104 FVGRSGR--------------------------------------------------GKS
BGIBMGA008905*   127 FVGRSGR--------------------------------------------------GKS
Tc05566*          96 FVGRSGRGAHWALSRLWARVPHLTVRVYRRSGVGESVTRTPPPPPAATGWYEPGSRSGKS
AAEL006160*       96 FVGRSGR--------------------------------------------------GKS
Nvi538861        200 FVGRSGR--------------------------------------------------GKS
Tc06536          154 FVGRSGR--------------------------------------------------GKS
AAEL006167        93 FVGRSGR--------------------------------------------------GKS
Nvi541634        102 FVGRSGR--------------------------------------------------GKS
Nvi541690        194 FVGRSGR--------------------------------------------------GKS
BGIBMGA008970*   122 FVGRSGR------------------EMYLRN----------------------SIVTGKS
GB15836          175 FVGRSGR--------------------------------------------------GKS
GB16431          264 FVGRSGR--------------------------------------------------GKS
SpRUNT1          143 FVGRSGR--------------------------------------------------GKS
Tc06542          104 FVGRSGR--------------------------------------------------GKS
Dmrun            192 FVGRSGR--------------------------------------------------GKS
CG34145          185 FVGRSGR--------------------------------------------------GKS

GB19482          183 FTLTITVSTTP-PQVATYTKAIKVTVDGPREPRSKTR----------QQQQFHAFAFASQ
Nvi538857        219 FTLTIMLQTSP-PQIATLSKAIKVTVDGPREPRSKTR-----------HQAFHPFHFGPR
CG42267          206 FTLTIVISTNP-IQIATYTKAIKVTVDGPREPRSKVR-----------HQGFHPFAFGPQ
GB11654          110 FSLTIQISTVP-FQVATYTKAIKVTVDGPREPRSKSN-----------------YQYGH-
Dmlz             371 FTLTITVATSP-PQVATYAKAIKVTVDGPREPRSKTSPT--------GGPHYRALGLGQR
Tc05796*         139 FSITITVSTTP-PQVATYTKAIKVTVDGPREPRSKTTG---------QHTAYRAIGLGQR
AAEL007040*      164 FTLSITVSTTP-PQVTTYTKAIKVTVDGPREPRSKTNTP--------QLRPPPLHRFLEQ
BGIBMGA008907*   132 FSLTITISTNP-PQVATYQKAIKVTVDGPREPRSKTKQT---------CAQVRGAWVADG
AAEL007036*      152 ---------------WNFSSSIYVTIY---------------------------------
BGIBMGA008906    114 FTLTITISSFP-SQVATYTKAIKVTVDGPREPRTKQN-----------------YGYGH-
BGIBMGA008905*   137 FTLTIMLATSP-PQVATYQKAIKVTVDGPREPRERVQ-----------ALGVSSLDAPCR
Tc05566*         156 FTLTIMVSTSP-PQVATYNKAIKVTVDGPREPRSKTR----------QQQQFH-FAFG-Q
AAEL006160*      106 FTLTITISTSP-PQVATYNKAIKVTVDGPREPRSKTR----------QQQQFH-FAFG-Q
Nvi538861        210 FTLTITVSTTP-PQVATYAKAIKVTVDGPREPRSKTR----------QQQPFHPFAFASQ
Tc06536          164 FTLSIIVSTTP-PQIATYNKAIKVTVDGPREPRSKTR-----------QQGFHHFPFGPR
AAEL006167       103 FSITITISTYP-CQIATYTKAIKVTVDGPREPRSKQN-----------------FAYGH-
Nvi541634        112 FSLTIQISSVP-FQVATYTKAIKVTVDGPREPRSKSN-----------------YQYGP-
Nvi541690        204 FSITITVSTTP-PQVATYTRAIKVTVDGPREPRSKTNALLDLVPGQ-QHQQFRALGLGQR
BGIBMGA008970*   142 FTLTITVSTTP-PQVTTYNKAIKVTVDGPREPRSKTMLSLLG-----QQQQFH-FAFG-Q
GB15836          185 FTLTIMLQTSP-PQVATLSKAIKVTVDGPREPRSKTR-----------HQAFHPFHFGPR
GB16431          274 FSITITVSTTP-PQVATYTRAIKVTVDGPREPRSKTRQTHIPGLAR-VPALPRVPGLTRG
SpRUNT1          153 FTLSIFIYTNP-PQIATYNRAIKVTVDGPREPRRPKPKDQESRLMPPPIINTG-------
Tc06542          114 FTLTITISSEPYYQIATYNKAIKVTVDGPREPRTKSN-----------------YQYGYG
Dmrun            202 FTLTITIATYP-VQIASYSKAIKVTVDGPREPRSKQS-----------------YGYPH-
CG34145          195 FTLTITVSTNP-PHIATYNKAIKVTVDGPREPRSKTR----------QQQQFH-FAFG-Q
```

```
GB19482          232 RGGPFFASPLVDPLQPLPNPLQPLPNPLQPRDPLSSFRHAMPANCQNMSQFGLTASN---
Nvi538857        267 ------------------------PFPFGHPQDPLGFKLS------GLQHLGLDQGA---
CG42267          254 R-----------------------FGPDPL---MAGLPFKLPGFAHHLVGMHSHLHA---
GB11654          151 -----------------------------------GFPGLGLLNPWVDVAYLGHAW----
Dmlz             422 PYIDGFPS-------------------TKALHELESLRRSAKVAAVTTAAAAAATAASAA
Tc05796*         189 PFLDG------------------------------SFSTHLRDLEAYKKQR---------
AAEL007040*      215 PAFSHFP---------------------------ARFNSSANKSSINTYSTSASHHQ---
BGIBMGA008907*   182 GIGRPSE----------------------------TVPAAARRSVGDLNLG---------
AAEL007036*          ------------------------------------------------------------
BGIBMGA008906    155 ---------------------------------PGPFSPFLLNPGWLDAAYLNYAWA---
BGIBMGA008905*   185 R-----------------------WIIKLQRFDTGGIETNEITGELARKGAETSSAG---
Tc05566*         203 R-----------------------PFPFAASDPLSGFRMPPIGNCNNMPQFGLSSTN---
AAEL006160*      153 R-----------------------PFHFPA-DPLSSFRMPPID----MSQFGLGTAN---
Nvi538861        259 RGGPFFASPLVDPLQPLPNPLQSLPNPLQPRDPLSSFRHAMPGNCQNMSQFGLTAGN---
Tc06536          212 T-----------------------FAPDPL---AGSLPFKLSVGQRSRAAGDKATEV---
AAEL006167       144 ---------------------------------PGAFNPFMLNPGWIDAAYMNYAWS---
Nvi541634        153 -----------------------------------GFPALGLLNPWLDAAYFSHAW----
Nvi541690        262 PYLDGPN----------------------------SFTNHLRELE-YRRSKHHPHHHHHH
BGIBMGA008970*   194 R-----------------------PFPFPP-DPLGGFRMPPITTCQNMSQFGLSSSN---
GB15836          233 ------------------------PFPFGHPQDPLGFKLTDPLDCTGLQHLGLEQG----
GB16431          332 TFPDAPS----------------------------PFTPYLRDSEPYRRNKHHVGNN---
SpRUNT1          205 -----------------------------HPHPFGEINPHHPNHHIGRQQSYQNQGR---
Tc06542          157 L-----------------------------PGMPAGFNPFLLNPGWLDAAYMSYTWP---
Dmrun            243 ---------------------------------PGAFNPFMLNPAWLDAAYMTYGYA---
CG34145          242 R-----------------------PFHFST-DPLSGFRMPPIG----NCQS---ASN---

GB19482          289 ---------------------------------S-WGYGSTA-GYAGYLPG-PLSSCAAQ
Nvi538857        294 ---------------------------------AAAAAAQGWGGLSRGGLPPHCLPPPPG
CG42267          285 ---------------------------------PDWRAHMALGGRPAAFTAAPFFGHHAA
GB11654          172 ----------------------------------HLPHPAFVKG--------------TI
Dmlz             463 NAVAAAAAAVAVTPTGGGGGVAAGGVAGGAGAGLVQQLSSNYSSPNSTINSDCQVYKPNA
Tc05796*         210 ----------------------------------------GGPSSQS--------SDGSQ
AAEL007040*      245 ----------------------------------------NSNSGSSSLNTSVESSSDYK
BGIBMGA008907*   205 ---------------------------------------VTTKLRPRKLSRQPVYYHPAY
AAEL007036*          ------------------------------------------------------------
BGIBMGA008906    179 ---------------------------------DYFRPPQMREPSTLIKGGAAPLTTPPV
BGIBMGA008905*   219 ---------------------------------LEPSTSTINIHWHPNSIGGSPLNADGS
Tc05566*         237 ---------------------------------SHWGYGAAG-AYSPYFTPSTLGSCAAP
AAEL006160*      182 ---------------------------------SHWSYSSTG-PYSPYLTS-----CATP
Nvi538861        316 ---------------------------------S-WSYGSTA-GYAGYLPG-PLSSCAAQ
Tc06536          243 ---------------------------------MSERVDSKTPHWS------------GG
AAEL006167       168 ---------------------------------DYFRQHQQLQAQQQASQPNPVTAAGKG
Nvi541634        174 ----------------------------------HLPHPALAKG--------------SI
Nvi541690        293 QQQQQQQL------------ATAQLQSPVSGDNANVNSSTTSPSSGSHLTNTTGGATAHQ
BGIBMGA008970*   227 ---------------------------------THWGYGGAS-AYPAYLP-----SCAAP
GB15836          265 -------------------------------------ASQGWGGLPRGSLPPHCLPPPPG
GB16431          361 --------------------VTANSVSGACT-NPNNTDPTASPASSAHLGPNSG-SSAHQ
SpRUNT1          233 -----------------------------------MPRSYPLSPTSGSYDNIQHQGQASK
Tc06542          185 ---------------------------------DYFRARTNVPTQTNLH---------AS
Dmrun            267 ---------------------------------DYFRHQAAAQAAQVHHPALAKSSASSV
CG34145          268 ---------------------------------THWGYGSAASAYSPYLASSGLSSCTTP
```

```
GB19482          313 ASFPPPPPPPPPPPP-----------------SSSSLASFAGAASMN-TPTAPDSTAG--
Nvi538857        321 HHSHAHPAAAAAAAA-------------VPQPTTAAAAADGTATAATAAAAAVAASAFNP
CG42267          312 AFPTASGLRGLSGDSQ-----------QHQQQQQQHQLATVGAAHSTTSPEGSPTTTTTS
GB11654          184 PMPSTDLFPPT---------------------FPPSVLPS--------YPF-DH-VKY--
Dmlz             523 PHIQAAEMMGAGEWTNGS-----------------SSSAAAYYHSHAHHPHAHHAHAHLQ
Tc05796*         222 SSYKQDSQDGSL-----------------------HPNPPPCPPATWP------------
AAEL007040*      265 PNLTLPDYSNATDWPNGL-----------------NSSSTSAYVSNYSP-----------
BGIBMGA008907*   226 FCPKAVMRFG--------------------------------------------------
AAEL007036*          ------------------------------------------------------------
BGIBMGA008906    206 TIPGADLFPFP---------------------PAVTNLPPGG------LIPPPGAFLPPN
BGIBMGA008905*   246 EETLLLGLYNRFYYAS-----------SLKVGGGIHGLRLPLAYQVDLEPVQRKNNMNSV
Tc05566*         263 TASQFNTPALGFSGS---------------TPDQTSTQDAF-GSTSNVTSLLPDTSTTDL
AAEL006160*      203 TAAQFNNPALGFTCS---------------SGEQNAGQDFT-STGRDCVPMLPDSTAADL
Nvi538861        340 TPFAAPPPPPPPPPTG-------------ISSTGSALTSFTGGTAMNNTPAAPDSTAGNN
Tc06536          258 AASDLIGIAHHL--------------------AGLPGPPPEWAMLGGRHPYPPGPFVPHH
AAEL006167       195 RNSSNTQLFSSSLFL-----------------AGSPTLPTPNGTTGPLLPTPPADFMSLS
Nvi541634        186 GMPHTDLFGPS---------------------FPPTVLPTG-------YPLTDHNVKYNS
Nvi541690        342 DCYKHSPQHGDTSVGTGGGGGGGSNSGATTATEWAYPSAAPSYPAPAGSAAGGAATAGGG
BGIBMGA008970*   248 AASQFNPPALGFAGT---------------VPDQTATQDFT-ANN----TVLPDTTGVDL
GB15836          288 HHSHTHPPAAGASAFNPFHHSIEQRSPRLPGPNSEPSRNDLGPISVSVREVTPTTSPGNP
GB16431          399 DCYKHSPQHGDT--GTAG------------SAEWTYPSAAPSYPAPPVSSGG--------
SpRUNT1          258 PWSYYNPYQSSVAQLS-----------------DTSILSAQIKTEPTELALLGQQNSTLQ
Tc06542          203 LIKGATQLPPT---------------------NGEFYLPPQP------QFHPPHNFLPPN
Dmrun            294 SPNPNPSVATS---------------------SSSAVQPSE-------YPHPAAAVAAAA
CG34145          295 TSAQFNNPALGFTCS---------------SNDQSNNQDFGGATNRDCVPMLPDSTASDL


GB19482          353 ---------STVTS----------------------------------------------
Nvi538857        368 FHHVAAAAAIEQRSPRLPATAN---------------GLHLLGATNSLFGSIFAPLLPQS
CG42267          361 GTQLSAFVQPPMTSSPPPVTSLQH-----------------------------------D
GB11654          211 ------------------------------------------------------------
Dmlz             566 HQMALPPPPPPPAAAPVSVGVGGN--------------------------GATMGMGMGV
Tc05796*         247 ----DYSYTP--------------------------------------------------
AAEL007040*      297 ----LQTTPPS-------------------------------------------------
BGIBMGA008907*   236 ------------------------------------------------------------
AAEL007036*          ------------------------------------------------------------
BGIBMGA008906    239 G------LLPFPPH----------------------------------------------
BGIBMGA008905*   295 ALYFGSSSWAKDRGYICDLGHSSL-----------------------------------V
Tc05566*         307 DQHLG-LVSSQNHTNHSQ------------------------------------------
AAEL006160*      247 DQHLSSLVGTQQTAQMTHPSLM----------------TTNGSSN--TPGNGTNGNGVTS
Nvi538861        387 GPATVPVPVANAAT----------------------------------------------
Tc06536          298 HPHFPPHMFA---------ALDRP-----------------------------------I
AAEL006167       238 GAQPTPNGLPTPTG----------------------------------------------
Nvi541634        218 T------------T----------------------------------------------
Nvi541690        402 SGSGSYSPIPAGA-----------------------------------------------
BGIBMGA008970*   288 DQQLSGLVGS--------------------------------------------------
GB15836          348 GPGLLTTATVAAPTPPAEPTTTTTTPSPSSGHHSHFQGLHLLGATGSIFGSIFAPLLPQS
GB16431          437 ----SFSPIPGGA-----------------------------------------------
SpRUNT1          301 QYPKPDSLYPTSITR---------------------------------------------
Tc06542          236 G------ILP--------------------------------------------------
Dmrun            326 G-QPAA-MMPSPPG----------------------------------------------
CG34145          340 DQHLSSLVG-STSGQMTHHSLLG------AGGQTSISSTVNGASGGGSAGAGTAGGGAGS
```

```
GB19482          358 ------GTGAGSTAQQDAFSAVSSLVPDSTTTG--QSDPLDPLSSLMSGT-PSQRYQDYV
Nvi538857        413 SWLYNPLYHTQQYILEPEWHALALRMAQQRLQRPDQARLEDLRKLRGSSASPEDVSKDK-
CG42267          386 NNNNNSNNNNSSSHIDAGFESDSISVTGSPRKSLGSPLTHDEEEAEAEAEAEAEAEAEAE
GB11654          211 ------PAEYATTLPPKSS----------SSSSAQAT---IPTSPSR-------------
Dmlz             600 GVGMGMNHYGGGYDSANSLEAGQYAAHLPAVLPEMHG--HGFATDP---YQTAGYGGGNT
Tc05796*         253 -----YSPQTGYYDHQDSATS----LHLPTVLPEP--GHNEFIN-----------TSLTS
AAEL007040*      304 --------YGG-YDPCPMAEHPNYN--LPAVLPDTAQTYQDYYSIP---PASGSTPHQAM
BGIBMGA008907*   236 --------LKGGAAVVTILETLELTSQVLPELPLGHADYSGFQSSTTS--YKGSPSGTSS
AAEL007036*          ------------------------------------------------------------
BGIBMGA008906    247 ------PAELALKSLPPELSLKSGLTPYEALRQFQNNVSSMDTSSAR-------------
BGIBMGA008905*   320 GWSTWRRMGSASPALPP-TNSASTSALHSTRAASSAYTAPRTPTASRKRVLKFLIFTDVT
Tc05566*         324 ------SSTHTSSLLVPRYST--NHSDFGLS----GPRSLSDNSSAAESP----VQDDIL
AAEL006160*      289 QANSITTTNGSNGLLVPRYQSNSSNNDYTLHTSQSGPRSLSD-SSQAESP----VQEDLL
Nvi538861        401 ------ANANNPAAQQDAFSSVSSLPDTTTPGV--QPDPLDHLSSLMSSTGSAQRYQDYV
Tc06536          314 NTSPRIANEPSSTSLGP-ISINCTPAHSTTSPKTSPTQVGLLTTAG--------------
AAEL006167       252 ------PVLPNGATYLPQFPFGPHADLHLKSPFLPYDISPLRANGLRGPHQLSATITLSS
Nvi541634        220 ------AADFASSMSHKSSSIHSMTPPPTTTPTAPPTSLLLPNSPSR-------------
Nvi541690        415 -----FSYPGEALAHHPTTEP----VPLPTVLPSD-GQQNAYTPNCVSMYPSGAASSTTS
BGIBMGA008970*   298 ------SPSHHG-TLLPRYN---NNADYSLST---GPRSLSDNSSQPESP----VQDDLL
GB15836          408 SWLYNPLYHTQQYVLEPEWHALALRMAQQRLQRPDQARVEETRKVRAGSSSPESALKEAK
GB16431          446 -----FSYPGEALSHHPTTEP----VPLPTVLPSDTSQQDTYTPNCVSMYPSHGASS-TS
SpRUNT1          316 -----SSEVQDPRFVYPSTPAAVSSVSFTPSSMSVLSSGVESPRTILPMTPNPFPLSSQD
Tc06542          240 ---------QLSLSDSP---------------LLRPSVGPMDQLSLR-------------
Dmrun            338 -------AAPATPYAIPQFPFNHVAAAAAAKAATPHAFHPYNFAAAAGLRARNAALHHQS
CG34145          393 GGGAGGGAGG-NSILVPRYHTNASN-EYNVHSSQNGPRSLSD-SSQAESP----VQEDLL

GB19482          409 SPRSLSTDS-----------------------------------------------STTE
Nvi538857        472 ----EEASRD---------------------------------------------LDSPD
CG42267          446 EAEVGGLSRNGGGIQ-----------------------------------GPLHSESSPG
GB11654          239 ------------------------------------------------------------
Dmlz             655 GGGSASKS----------------------------------------------ELDYGG
Tc05796*         291 PPPMLNSAK----------------------------------------------SELDP
AAEL007040*      350 SHGYVSKTT---------------------------------------------DLDTSY
BGIBMGA008907*   286 SLTELNPTTPVTTQR---------------------------------------YDSNYY
AAEL007036*          ------------------------------------------------------------
BGIBMGA008906    288 ------------------------------------------------------------
BGIBMGA008905*   379 SDNPISMRAHSTTSP-----------------------------------ANSRPCSPQD
Tc05566*         368 S----TQST-LG----VNHVN----------------------------------NTNFP
AAEL006160*      344 S----SNTPNLG-----GAAN-----------------------------------QNFS
Nvi538861        453 SPRSLSTDS-----------------------------------------------SATE
Tc06536          359 -----------------------------------------------------APLSPPE
AAEL006167       306 SD------------------------------------------------------FISS
Nvi541634        261 ------------------------------------------------------------
Nvi541690        465 SLALIQNPSSVKPLV---------------------------------------DTSSDM
BGIBMGA008970*   341 T----SNTTNIG----HNHSN----------------------------------TSNFP
GB15836          468 TRRDDEDNRDPARVRSG--------------------------------------PDSPD
GB16431          496 SLPLVPAKS----------------------------------------------SDPDI
SpRUNT1          371 IFSSSSTATP---------------------------------------------VTLTS
Tc06542          263 ------------------------------------------------------------
Dmrun            391 E----------------------------------------------------------P
CG34145          446 T----TNTPNLGSTAGGGAANGGAGSNAGSGAAGSGAGGAGGASSAVGNPAMLGANQNFP
```

```
GB19482          422 SPVHE-EQGFGQNY---------------------------------GNYFPTPGVLPSI
Nvi538857        483 GSIEVDDSEEPRPKIERR-----------------------------SQSEESLEQDSSP
CG42267          471 SGGAFTALIQRSGKNPTELFGG-------------FAAAGGNHFAPSGHSFNPALAAQLF
GB11654          239 --TPPKSPSESGS----------------------------------------ESAPEEV
Dmlz             669 SYNQAWSNGYQNYQYGSCLAT------------AQYGPQAA-------PPPQPPPPPPVV
Tc05796*         305 MRYSDNST-----YYPNNWTP------------NSYPNNYN-YYNT--PNNNQQYPSTMV
AAEL007040*      365 NPYSQWANGYGSYQYNAPCPP------------PLPPPQ----------PQYPSTAPAMV
BGIBMGA008907*   307 NSWPANSYNYNNYQYNNINNN--------PACIQSHAP--------------YINPNPQM
AAEL007036*          ------------------------------------------------------------
BGIBMGA008906    288 --LSPTSSRQSGSPRSMA----------------------------NASPDRSKTDSKSE
BGIBMGA008905*   404 DDISVTASMSPPPDERPGAFT--------------RPRKPLQPLPAPSSLFHSALAAQLF
Tc05566*         385 LHQNMTSSSYPSSN---------------------------------CNNSIYPVLPASL
AAEL006160*      360 SLVVNQSQSNYGASGG------------------------------SCNGSLYPVLPASL
Nvi538861        466 SPVHEQDQSFGQNCSA-------------------------------GNYFPAPSVFPTI
Tc06536          366 DDISVTASPTPSPQPPPAFPG--------------APPPPIQ----NNQLFNNALAASLF
AAEL006167       312 SRLSPASSRNSTSSPP-------------------------------STLNQSQIKMSIE
Nvi541634        261 --TPPRSPSDSGS----------------------------------------ESAPEEV
Nvi541690        486 YATATGSPGSTGYHHYGGWTTAPGPATPVPAPAN-YNPNYQGYYHQNPAAQNYIGQAPMV
BGIBMGA008970*   359 SLMGSQNSSYGSSN---------------------------------CNNSLYPVLPASL
GB15836          490 GSIEVDDRNEQESGKDR------------------------------ARSDESVGGQDSP
GB16431          510 FAGGGTGSGSPGYHYGPSWTS--GPATPVPVASHNYNPNYQGYYNN--PSQNYISPAPMV
SpRUNT1          386 PPYLPNSPPYPLYPHLYMSSP--------------------------SSQTYYDSSHLPM
Tc06542          263 --ISPVSNSQS------------------------------------------PTAQETK
Dmrun            393 VHVSPASSRPSSSSPT-------------------------------QQHVLLKLNTSIE
CG34145          502 GIVNQAQHSAASAYGGGTGVVG-------------GGHGSAAAAAAGCNGSLYPVLPASL

GB19482          448 LYSQLYGNQFQ---------------------NSES------------------------
Nvi538857        514 RTSPVRTDTELT-------QEETSSRLRPSLENVNEL---------DNSTTQLDHQLDQQ
CG42267          518 LQSPLLPQSSQWLYTQLYGSYSDLPWLRNAAAAAAANINPGQENSGIPPLGSNPDHDGVN
GB11654          257 R-SAFVPIRLNT------------LPPTTSVVTATS---------------------ASS
Dmlz             710 LCPQLYS-TVNQNQIHLHLHS---SEKLEQYLGTAT----------------------SA
Tc05796*         345 LYPPLYS---TQNQIHFHLHGS--SEKIDQYLSTES------------------------
AAEL007040*      403 LCPQMFS-TVNQNQIHVHLHGTG-SEKLEQYLGSENGF--------------------SI
BGIBMGA008907*   345 ILPNLYS-TVNQNQIHVHLHSSSDKYNLEQCIPSEIK-----------------------
AAEL007036*          ------------------------------------------------------------
BGIBMGA008906    318 ANSIHDATITD-------------ESDEEPIEVVKS---------------------AFH
BGIBMGA008905*   450 LNSPLLPTPPAWLYSQLYGG---------YDWWLRP----------PAPQE---DPSSSP
Tc05566*         412 LYSQLYSAANQ--------SHNFHSLHS---HTTQS-------------HHNDLQTVMDQ
AAEL006160*      390 LYSQLYTAANQ--------THGFHNHPLQNSHSTSQN---------ASMHAGELQSVMDH
Nvi538861        495 LYSQLYNNQFQ---------------------STEA------------------------
Tc06536          408 LNAPLLPPPGQWFYSQFYPHDWAWMNLRHHSLLPRS----------SSPLELGHNPNGST
AAEL006167       341 LNSTNEHSSGE-------------ESDDEHIDVVKS---------------------AFV
Nvi541634        279 VRSAFAPIRPNS------------N--NNNIHVPTS---------------------SSS
Nvi541690        545 LYPQVFTSTINQNQIHLHLS--------EETLANSN------------------------
BGIBMGA008970*   386 LYSQLYTAANQ--------THNFHPLHSNSIHSTQN-------------HHNELQTMMDQ
GB15836          520 RISPVRSDTEDTTADATAFQEDGTAHLRPSMENSNLD---------QEVLATDGHQVSRR
GB16431          566 LYPQLYS-TVNQNQIHLHLHGD--LSEEQVTIAGSN------------------------
SpRUNT1          420 LPSSTRPEDKQEIKHDNRPGEGIPHIPHPEMSLTVALN------------------YGSH
Tc06542          279 IHSTVDQSTSE-------------NSDDEDIDVVKS---------------------AFV
Dmrun            422 TSSIHEQSASDG------------DSDDEQIDVVKS---------------------EFD
CG34145          549 LYSQLYTAANQS-------AHGFHSHTLP--AHASPN---------SSVHG-ELQSVMDH
```

```
GB19482          463 ---------PEQ---------------RAVADS-CSVR-QEEVR----------------
Nvi538857        558 PASRGLTVKIRVEG-TVDLSTKTRTHHQDKENVGQDLTTTTRRKDDDVEISEREAIRPRR
CG42267          578 LIKRCVTLITHNPPDAENANPNASPPVSSTRRSPSPVETIDLDDVSTTSRSASGSSGHGG
GB11654          283 PERLP--TKKG--VVVEGSRNELKAP--------TALISRSMS--PKRSPSPTKIS-SPP
Dmlz             744 DHLTIGSLTGSSRSSIEIGQDQYHQQVHHAQQQQQQQQQQQQQVHHPQQQQVESAGEVGGS
Tc05796*         376 -------LSLTPTRAIELAQTVTTEVAQSHETLEDTERTHDP------------------
AAEL007040*      441 NSISSGGIRPSAVPGIEIGIATHDSSLVSNDGSHQAQQQPQ-----QQEQQHSAVEVSEQ
BGIBMGA008907*   381 ------ISDIDGGISITTELQGTGEPSGIVQTCEANDEVKHG------------------
AAEL007036*          ------------------------------------------------------------
BGIBMGA008906    344 PTRPANLELQEMKRVQAADSTVSDRPRTRNELKSTSQRTTRVLS--TSPTSTKIANGTIP
BGIBMGA008905*   488 DRE----EESSTPSVAGKKRP------ASPEWSDQSVR----------TRSKSLTTPEK-
Tc05566*         448 LS-------TSNQRQMNGST-----DLLLSNNGTCAAAAARQD---DARLTSNGAQRGPG
AAEL006160*      433 LTSSTANGGRQHPNLMTNN--PHT-DLSLIGNCGAMARGALEEPGARALAGGRGVPPQGQ
Nvi538861        510 ---------QEQQH-------------RALADVSCSVRVQEEVR----------------
Tc06536          458 KSEGVDDEVKSDDGKAKEEKK------GKDEGVNLSLHRCRKAAITLVRQKEEVSSSEKE
AAEL006167       367 PILRPAPTSSPPPPAPPSARCELKAPSSKKPQFHESAPSEPASPENTKLKSPNLIIKQS-
Nvi541634        304 PDRLS--NNNNSKRPIEGTRCELKAP--------KALISHRTKTLGKRSPSPTKIQPTTP
Nvi541690        573 -------LTISSNR-LEIGVLG-----------EENEQRND-------------------
BGIBMGA008970*   425 ISS------TTNNHRQHGNQ-----DLLLGGSSSCAAAAARGE---DGRVN-NLGQRGNP
GB15836          571 ES------KIRLEVGTVDLSTKNK--GQDKENVGQDLTT--KRKEEDTER--EAVVRGRR
GB16431          599 -------LTISSNR-LEIGVMG-----------EENEQRND-------------------
SpRUNT1          462 PQSQIELNTARTMGQSMGGMHNSGVALMQQHRNHSPVTNISPMQQSMGQMNINTTVGHLP
Tc06542          305 PIKPANLILQEVQ----PDSTVQDKELKKNELKAPSSRSIKSLAEEKSPNTKIHHQQEIT
Dmrun            449 -------LDKSLDVAPLRMRCDLKAPSAMKPLYHESGPGAVANSRQPSPETTTKIKSAAV
CG34145          590 ISN---VGVRQQHNIMAGGGVTHPGDLTLIGNCGASVR-NIEDGNSNRQVAALAAHRGHH

GB19482          481 ---------------PDNN---VWRPY--
Nvi538857        617 --------------ERSPKPRQVWRPY--
CG42267          638 GVGGGGAVGPIRTRTPKPSADVWRPY--
GB11654          328 ------------------PAKPVWRPY--
Dmlz             804 GAGG---VESAREEDVGDLSQVWRPY--
Tc05796*         411 --------------------ASVWRPY--
AAEL007040*      496 -------QDSREEAVTGDPSSVWRPY--
BGIBMGA008907*   417 -------------MYGAGSQEVWRPY--
AAEL007036*          ---------------------------
BGIBMGA008906    402 ------------------SHKSVWRPY--
BGIBMGA008905*   527 ------------------RPVDVWRPY--
Tc05566*         493 --------------QNSDA--VVWRPY--
AAEL006160*      490 --------------VQSENGNSVWRPY--
Nvi538861        532 ---------------PDNN---VWRPY--
Tc06536          512 -------------HLKCNRLGDVWRPY--
AAEL006167       426 -------------------AKTVWRPY--
Nvi541634        354 ------------------AVKTVWRPYPC
Nvi541690        595 ----------------------VWRPY--
BGIBMGA008970*   470 --------------QPDSN--TVWRPY--
GB15836          619 --------------ERSPKPRQVWRPY--
GB16431          621 ----------------------VWRPY--
SpRUNT1          522 -------------TVDDSRKEDVWRPY--
Tc06542          361 ------------------ATKSVWRPY--
Dmrun            502 ------------------QQKTVWRPY--
CG34145          646 --------------NPTDNGGSVWRPY--
```
