# Supplementary material for: Evolutionary origin and genomic organisation of runt-domain containing genes in arthropods
Source: BMC Genomics. 2008 Nov 25;9:558. doi: 10.1186/1471-2164-9-558 (PMC2631020; doi:10.1186/1471-2164-9-558)
Supplement: Additional file 3 — Multiple sequence alignment of full-length RD protein sequences from exopterygotan and endopterygotan insects. ClustalX alignment of full-length RD protein sequences from six insect species: Drosophila melanogaster, Tribolium castaneum, Acyrthosiphon pisum, Pediculus humanus, Bombyx mori and Apis mellifera. Alignment includes the outgroup Strongylocentrotus purpuratus. [file 1471-2164-9-558-S3.pdf]

Additional file A3: multiple sequence alignment of full-length RD protein sequences from a number of exopterygotan and endopterygotan insects.

```
BGIBMGA008970*     1 ------------------------------------------------------------
ApXP001948981*     1 ------------------------------------------------------------
Dmlz               1 MHLHLLAAEKTPPSPSPNPTPTPSASPSGHTGAAGESQLDLASQTESQLQLGLPLASGYG
Tc06542            1 ------------------------------------------------------------
PHUM008646         1 ------------------------------------------------------------
Tc06536            1 ------------------------------------------------------------
Tc05796*           1 ------------------------------------------------------------
PHUM003686*        1 ------------------------------------------------------------
PHUM003688*        1 ------------------------------------------------------------
SpRUNT1            1 ------------------------------------------------------------
GB15836            1 ------------------------------------------------------------
CG42267            1 ------------------------------------------------------------
Tc05566*           1 ------------------------------------------------------------
ApXP001947715      1 ------------------------------------------------------------
BGIBMGA008906      1 ------------------------------------------------------------
PHUM003687*        1 ------------------------------------------------------------
Dmrun              1 ------------------------------------------------------------
ApXP001950158*     1 -------MRVPLRRRRYRATTRRRGRADPRPRAATTNGGTVNQNPPGPHPAAVPTVATVG
GB19482            1 ------------------------------------------------------------
BGIBMGA008907*     1 ------------------------------------------------------------
BGIBMGA008905*     1 ------------------------------------------------------------
GB16431            1 --------MKSAVPRPPRITTDCRGSLWAIYGDGDDRQADIRKPDSGGSSATLLEYPGH-
Aphmm248704*       1 ------------------------------------------------------------
CG34145            1 ------------------------------------------------------------
GB11654            1 ------------------------------------------------------------


BGIBMGA008970*     1 ------------------------------------------------------------
ApXP001948981*     1 ------------------------------------------------------------
Dmlz              61 LGLGLGLGLGLGLGQELVADHSTTVAPVSVAGPGRLGRSINGSGGSHHHHHLHHHYSPYH
Tc06542            1 ------------------------------------------------------------
PHUM008646         1 ------------------------------------------------------------
Tc06536            1 ------------------------------------------------------------
Tc05796*           1 ------------------------------------------------------------
PHUM003686*        1 ------------------------------------------------------------
PHUM003688*        1 ------------------------------------------------------------
SpRUNT1            1 ------------------------------------------------------------
GB15836            1 ------------------------------------------------------------
CG42267            1 ------------------------------------------------------------
Tc05566*           1 ------------------------------------------------------------
ApXP001947715      1 ------------------------------------------------------------
BGIBMGA008906      1 ------------------------------------------------------------
PHUM003687*        1 ------------------------------------------------------------
Dmrun              1 ------------------------------------------------------------
ApXP001950158*    54 G-----------------------------------------------------------
GB19482            1 ------------------------------------------------------------
BGIBMGA008907*     1 ------------------------------------------------------------
BGIBMGA008905*     1 ------------------------------------------------------------
GB16431           52 ------------------------------------------------------------
Aphmm248704*       1 ------------------------------------------------------------
CG34145            1 ------------------------------------------------------------
GB11654            1 ------------------------------------------------------------
```

```
BGIBMGA008970*     1 ------------------------------------------------------------
ApXP001948981*     1 ------------------------------------------------------------
Dmlz             121 HAHPYHPPHPHAPHHHHHHHPPYPYPPAGPHPPAMVTSSSTSPTGNGWSSSTGDFKGITA
Tc06542            1 -------------------------------------------------MHLPINAQQPS
PHUM008646         1 -------------------------------------------------MHLPSPRDRAA
Tc06536            1 ------------------------------------------------------------
Tc05796*           1 ------------------------------------------------------------
PHUM003686*        1 ------------------------------------------------------------
PHUM003688*        1 ------------------------------------------------------------
SpRUNT1            1 ------------------------------------------------------------
GB15836            1 --------------------------------------------MTNPLRDARPEDRPLG
CG42267            1 ---------------------------------------------MHISAEVSSTTSNQI
Tc05566*           1 ------------------------------------------------------------
ApXP001947715      1 ------------------------------------------------------------
BGIBMGA008906      1 -------------------------------------------------MHLPHASPAVR
PHUM003687*        1 ------------------------------------------------------------
Dmrun              1 -------------------------------------------------MHLPAGPTMVA
ApXP001950158*    55 ---------------NRTAARSAVRWRRPHAFCGIKTPAARKEGKAGGRGVVYGAAAARG
GB19482            1 ----------------------------------------------MDIFGRCNGGGGGS
BGIBMGA008907*     1 ------------------------------------------------------------
BGIBMGA008905*     1 ------------------------------------------------------------
GB16431           52 ---------------------PSCEPEAEKQLLEKKLLVAAIAEESPWSVQIEEASEITT
Aphmm248704*       1 ------------------------------------------------------------
CG34145            1 ----------------------------------MEHEGANSNNYKMHLTTTSSNSTASN
GB11654            1 -------------------------------------------------MHLPEGPLGMD

BGIBMGA008970*     1 -------------------------------------MHLTGANSGAVSPDTSSTLLHET
ApXP001948981*     1 -------------------------------MVVKGAQMTGGVGGIAATGSGSDDGLSDA
Dmlz             181 VATATGGGVGGATQGATASTGATAAEVLAVSSSASVGSSSPTGGASNGTAHSGHSGHTGG
Tc06542           12 ------------------------------------------------------------
PHUM008646        12 MAG---------------------------------------------------------
Tc06536            1 MGQSESCGPPPQHHSRDFSSHFRAVRWP--RHAAKKLTSMHLATGEVTSTTGHYDRSYGN
Tc05796*           1 -------------------------MHLPVSLSSC--TVSEEAWPKSSGMCEPSSGYVSN
PHUM003686*        1 ---------------------------MHLPAPATSISTGKRRRKMAADQTGGDNTSANG
PHUM003688*        1 ------------------------------MHPGTDVDGSS---------------PADY
SpRUNT1            1 ------------------MHITDVNVDHLLSSTAPLANHPSKDPVRRNNLHNSYKMAEGG
GB15836           17 LDYPDPRLVRNGHQEHQGNTTAHQIGAARIRRTRTSSTRRRMHLANEVPPQGSTGGNNLP
CG42267           16 QQQQQQHQQQQQHQLLQHQQQQTATTTTTKRRNAESSASSNNNNNNNTSTTNNNNTNNNN
Tc05566*           1 ------------------------------------------------------------
ApXP001947715      1 ---------------------------MRLVRMTSDHSKKRRRGPSSADQHGVATASSSS
BGIBMGA008906     12 ------------------------------------------------------------
PHUM003687*        1 ----------------------------------MHLTGATPTPGTASSPEGTASVLNET
Dmrun             12 NNTQVLAAAAAAAAAAAAAVAQGPGPQQSSNATTASAIAINPAQSLANTSTHSASSTGSS
ApXP001950158*   100 AVGRRWSRAPRTHIIIIYIRGSVVGGAAAAVGARVKKNPDEKHTTVSGGRSGGGGGGGGG
GB19482           15 STVSGSGAGSVRGSGNESSCGTASGASESGGTSDRMQLTGASAPGVAASPESGVSPLTDA
BGIBMGA008907*     1 ------------------------------------MRFGLKGGAAVVTILETLELISQG
BGIBMGA008905*     1 ------------------------------MHLASEVSSTTNGLGHEAPSSSYNGGSLPS
GB16431           91 SCPEG-------TCLGDEGRQLADGMHLPVGSVGCGENYATVGSHEGAGPCGPSLAPLQG
Aphmm248704*       1 --------------------------------------------------MHLSEPMRNR
CG34145           27 ANNNNNNTANNNNTSSNNNTTNTNGSSNNNTSGNNNNSSGNNSNTEQNTPPTPAQLLNEA
GB11654           12 ------------------------------------------------------------
```

```
BGIBMGA008970*    24 YT----------------------KMTSDILAERTLGDFLSEHPGELVRTGSPHFVCTVL
ApXP001948981*    30 YA----------------------KMTADILAERSLGDFLSEHPGELVRTGSPFLVCTIL
Dmlz             241 HSSSTASNNNNNGASNSNSNNNNAVHQDLLWMERLVQKRQQEHPGELVRTSNPYFLCSAL
Tc06542           12 ----------------------------IMDMYASLQETLQEYHGELVQTGSPAVLCSVL
PHUM008646        15 -----------------------------TDLFSNVHEILQEYHGELVQTGSPAILCSAL
Tc06536           59 -------------------------LTAEMLAERTIDGLLAEHPGELVRTGSPHVVCTVL
Tc05796*          34 N-------------------------QDIWWTEHLIHEIQTEHPGELVRTGSPYFLCSAL
PHUM003686*       34 ---------------------------DLWWTERILHEIQAEHPGELVRTGSPYFLCSVL
PHUM003688*       16 HV-----------------------LTAEALAERTLDGLLAEHPGELVRTGSPHLVCTML
SpRUNT1           43 QRNKASS--------------------VFKGGERSIVDALSEYPGELVKTESPNFACSVL
GB15836           77 DY----------------------ALTADLLTERTLDGLFAEHPGELVRTGSPHVVCTVL
CG42267           76 NSTTNNNNNNNNNNVKTKPVDTSPYLTPENLIERTVDVLLAEHPGELVKTGSPHVVCTTL
Tc05566*           1 -------------------------MTSDILAERTLNDFLSEHPGELIRTGSPLFVCTVL
ApXP001947715     34 SSSSPPS-------------------EDLWWTEAAVNEVTAEHPGELVRTGCPYMLCSAL
BGIBMGA008906     12 ----------------------------MADVYAHIHEYYRQSHGELVQTGSPAVLCSAL
PHUM003687*       27 YT----------------------KMTSDILAERTLGDFLSEHPGELVRTGSPHFVCTVL
Dmrun             72 TPDLSTNNTSSSSNATTSPQNSAKMPSSMTDMFASLHEMLQEYHGELAQTGSPSILCSAL
ApXP001950158*   160 GGSGGSAAARGGDDITRGPVVRDIDAVYQKYVACILEDVRAIHGKEMVATGSPSVFCSVL
GB19482           75 YT----------------------KMTSDILAERTLGDFVSEHPGELVRTGSPHLVCTVL
BGIBMGA008907*    25 E-----------------------------------WRIGGLTFLVAVKTGSPDYMCSIL
BGIBMGA008905*    31 QP-----------------------LTAELLAERTLEGLLADHPGELVKTGSPH-VCTVL
GB16431          144 VNTRYQQADDADAGGGGGEMSEGAAVGELWWTERLVGEAQAEHPGELVRTGSPYFLCSQL
Aphmm248704*      11 DG----------------------FLKADVLAERTLDSLLAEHPGELVRTGCPHVVCTVL
CG34145           87 YT----------------------KMTSDILAERTLGDFLTEHPGELIRTSSPLFVCTVL
GB11654           12 --------------------------------INAMHETLQACHGDLVRTGSPAILCSAL

BGIBMGA008970*    62 PPHWRSNKTLPVAFKVVALGDIGDGTLVTVRAGNDENCCAELRNSSAVMKNQVAKFNDLR
ApXP001948981*    68 PTHWRSNKTLPVAFKVVALGEVPDGTAVTIRAGNDENFCAELRNCTALMKNQVAKFNDLR
Dmlz             301 PAHWRSNKTLPMAFKVVALAEVGDGTYVTIRAGNDENCCAELRNFTTQMKNDVAKFNDLR
Tc06542           44 PSHWRSNKSLPIAFKVVALDEVRDGTTVTLKAGNDENYCAELRNCTAVMKNQVAKFNDLR
PHUM008646        46 PNHWRSNKSLPIAFKVVALDDVVDGTLVTIRCGNDENFCGELRNCTAVMKNQVAKFNDLR
Tc06536           94 PPHWRSNKTLPVAFKVVALGDVGDGTVVTVRAGNDENYCAELRNCTAVMKNQVAKFNDLR
Tc05796*          69 PTHWRSNKTLPVAFKVVALGDIGDGTIVTVRAGNDENCCAELRNSTAVMKNQVAKFNDLR
PHUM003686*       67 PTHWRSNKTLPIAFKVVALGDVMDGTIVTVRAGNDENCCAELRNCTAVMKNQIAKFNDLR
PHUM003688*       53 PPHWRSNKTLPVAFKVVALGEVLDGTLVTVRAGNDENFCGELRNCTAVMKNQVAKFNDLR
SpRUNT1           83 PNHWRCNKSLPVAFKVVSLGETKDGTMVTIAAGNDENYCAELKNNTAVMKNQVARFNDLR
GB15836          115 PAHWRSNKTLPVAFKVVALGEVGDGTLVTVRAGNDENCCAELRNSTAVMKNQVAKFNDLR
CG42267          136 PTHWRSNKTLPIAFKVLALGEVMDGTIVTIRAGNDENFCGELRNCTAVMKNQVAKFNDLR
Tc05566*          36 PPHWRSNKTLPVAFKVVALGDVGDGTVVTVKAGNDENYCAELRNSTAVMKNQVAKFNDLR
ApXP001947715     75 PTHWRSNKTLPGAFKVVILSEVPDGTAVTLKAGNDENCSAELRNCSALVKNQIAKFNDLR
BGIBMGA008906     44 PGHWRSNKSLPLAFKVVALDDVQDGTLVTIKAGNDENVMAELRNCTAVMKNQVAKFNDLR
PHUM003687*       65 PPHWRSNKTLPVAFKVVALGDVIDGTLVTVRAGNDENYCAELRNGTAVMKNQVAKFNDLR
Dmrun            132 PNHWRSNKSLPGAFKVIALDDVPDGTLVSIKCGNDENYCGELRNCTTTMKNQVAKFNDLR
ApXP001950158*   220 PGHWRSNKSLPIPFKVVVLDEVPDGAVVVVQAGNDENPSADMRNYRALSATGIAVFNDLR
GB19482          113 PAHWRSNKTLPVAFKVVALGEVGDGTLVTVRAGNDENCCAELRNSTALMKNQVAKFNDLR
BGIBMGA008907*    50 PQHWRSNKTLPGGFKVVALGDVLDGTLVTVRAGNDENCSAELRNNSAVMKNRVAKFNDLR
BGIBMGA008905*    67 PPHWRSNKTLPVAFKVVALGDVGDGTLVTVRAGNDENCSAELRNCTAVMKNQVAKFNDLR
GB16431          204 PTHWRSNKTLPVAFKVVALGEVVDGTLVTVRAGNDENCCAELRNSTTLMKNQVAKFNDLR
Aphmm248704*      49 PTHWRSNKTLPVAFKVVALGEVPDGTPVTIRAGNDENFCAELRNCTALMKNQVAKFNDLR
CG34145          125 PPHWRSNKTLPVAFKVVSLGDIMDGTMVTVRAGNDENYCAELRNCTAVMKNQVAKFNDLR
GB11654           40 PSHWRSNKSLPVAFKVVALDDVSDGTLVTIRAGNDENCCGELRNCTAVMKNQVAKFNDLR
```

```
BGIBMGA008970*   122 FVGRSGR------------------EMYLRN----------------------SIVTGKS
ApXP001948981*   128 FVGRSGR--------------------------------------------------GKS
Dmlz             361 FVGRSGR--------------------------------------------------GKS
Tc06542          104 FVGRSGR--------------------------------------------------GKS
PHUM008646       106 FVGRSGR--------------------------------------------------GKS
Tc06536          154 FVGRSGRG--------------------------------------------------KS
Tc05796*         129 FVGRSGR--------------------------------------------------GKS
PHUM003686*      127 FVGRSGR--------------------------------------------------GKS
PHUM003688*      113 FVGRSGR-----------------------------------------------------
SpRUNT1          143 FVGRSGR--------------------------------------------------GKS
GB15836          175 FVGRSGR--------------------------------------------------GKS
CG42267          196 FVGRSGRG--------------------------------------------------KS
Tc05566*          96 FVGRSGRGAHWALSRLWARVPHLTVRVYRRSGVGESVTRTPPPPPAATGWYEPGSRSGKS
ApXP001947715    135 FVGRSGR--------------------------------------------------GKS
BGIBMGA008906    104 FVGRSGR--------------------------------------------------GKS
PHUM003687*      125 FVGRSGR-----------------------------------------------------
Dmrun            192 FVGRSGR--------------------------------------------------GKS
ApXP001950158*   280 FVGRSGR--------------------------------------------------GKL
GB19482          173 FVGRSGR--------------------------------------------------GKS
BGIBMGA008907*   110 FVGRSGRSYVREIIQY--------------------------------------FVPGKS
BGIBMGA008905*   127 FVGRSGRG--------------------------------------------------KS
GB16431          264 FVGRSGR--------------------------------------------------GKS
Aphmm248704*     109 FVGRSGR--------------------------------------------------GKS
CG34145          185 FVGRSGR--------------------------------------------------GKS
GB11654          100 FVGRSGR--------------------------------------------------GKS

BGIBMGA008970*   142 FTLTITVSTTP--PQVTTYNKAIKVTVDGPREPRSKTMLSLLGQQQQFH-FAFGQR----
ApXP001948981*   138 FTLTITVSCSP--PQVTTYNKAIKVTVDGPREPRSKTR-----QQQQFHAFAFGQR----
Dmlz             371 FTLTITVATSP--PQVATYAKAIKVTVDGPREPRSKTS------PTGGP-----------
Tc06542          114 FTLTITISSEPY-YQIATYNKAIKVTVDGPREPRTKSN----------------------
PHUM008646       116 FSLTILISSTP--FQIATYAKAIKVTVDGPREPRTKTS----------------------
Tc06536          164 FTLSIIVSTTP--PQIATYNKAIKVTVDGPREPRSKTR------QQGFHHFPFGPRTFAP
Tc05796*         139 FSITITVSTTP--PQVATYTKAIKVTVDGPREPRSKTT------GQHTA-----------
PHUM003686*      137 FTITITISSSP--PQVTTYTKAIKVTVDGPREPRSKTR------KNMNEILPRFFYYSG-
PHUM003688*      120 ----------------APEPKRILESGDGKLSSKRTSS------PIGN-----------I
SpRUNT1          153 FTLSIFIYTNP--PQIATYNRAIKVTVDGPREPRRPKP------KDQESRLMP-------
GB15836          185 FTLTIMLQTSP--PQVATLSKAIKVTVDGPREPRSKTR------HQAFHPFHFGPR----
CG42267          206 FTLTIVISTNP--IQIATYTKAIKVTVDGPREPRSKVR------HQGFHPFAFGPQRFGP
Tc05566*         156 FTLTIMVSTSP--PQVATYNKAIKVTVDGPREPRSKTR-----QQQQFH-FAFGQR----
ApXP001947715    145 FTITITVSTCP--PQVATYNKAIKVTVDGPREPRSKSL------INVHIYIGKFVLFVFP
BGIBMGA008906    114 FTLTITISSFP--SQVATYTKAIKVTVDGPREPRTKQN----------------------
PHUM003687*      132 -------------------------------------------DMSQFG-LSTG------
Dmrun            202 FTLTITIATYP--VQIASYSKAIKVTVDGPREPRSKQS----------------------
ApXP001950158*   290 LTLTITVQCKDQAVLVANYVKAIKITVDGPRLPRSNHR----------------------
GB19482          183 FTLTITVSTTP--PQVATYTKAIKVTVDGPREPRSKTR-----QQQQFHAFAFASQRGGP
BGIBMGA008907*   132 FSLTITISTNP--PQVATYQKAIKVTVDGPREPRSKTK------QTCAQVR---------
BGIBMGA008905*   137 FTLTIMLATSP--PQVATYQKAIKVTVDGPREPRERVQ------ALGVSSLDAPCRRWII
GB16431          274 FSITITVSTTP--PQVATYTRAIKVTVDGPREPRSKTR------QTHIPGL---------
Aphmm248704*     119 FTLTIMLNSCP--PQVATYSKAIKVTVDGPREPRSKTR------NQGFHPFHFGPR----
CG34145          195 FTLTITVSTNP--PHIATYNKAIKVTVDGPREPRSKTR-----QQQQFH-FAFGQR----
GB11654          110 FSLTIQISTVP--FQVATYTKAIKVTVDGPREPRSKSN----------------------
```

```
BGIBMGA008970*  195 --------------------PFPFPP-DPLGGFRMPPITTCQNMSQFGLSSSNTHWGYG-
ApXP001948981*  187 ----------PFLSTHFANPLDPLHRTADPLAFRMPAMANCQNMGQF---APHHSWGYS-
Dmlz            412 -------HYRALGLGQRPYIDG-FPSTKALHELESLRRSAKVAAVTTAAAAAATAASAAN
Tc06542         151 -------------------YQYGYGLPGMPAGFNPFLLN-PGWLDAAYMSYTWPDYFRAR
PHUM008646      152 -------------------FGYGLAGG--YPGFAPFAVVPPQWLDAAYMSYAWPEYFRRP
Tc06536         216 DPLAGSLPFKLS------VGQRS--RAAGDKATEVMSERVDSKTPHWSGGAASDLIGIAH
Tc05796*        180 --------YRAIGLGQRPFLDG--SFSTHLRDLEAYKKQRG-------------------
PHUM003686*     188 ----------------------------------TDSSVNCATTGTTEPAWGYSSSGYPT
PHUM003688*     147 SPKKHTSG------------PESPKKKSPSLSPGKRLL------NTIKQHKTNTGETPSK
SpRUNT1         198 -------PPIINTGHPHPFGEINPHHPNHHIGRQQSYQNQGRMPRSYPLSPTSGSYDNIQ
GB15836         233 --------------------PFPFGHPQDPLGFKLTDPLDCTGLQHLG-LEQGASQGWGG
CG42267         258 DPLMAGLPFKLPGFAHHLVGMHSHLHAPDWRAHMALGGRPAAFTAAPFFGHHAAAFPTAS
Tc05566*        204 --------------------PFPFAASDPLSGFRMPPIGNCNNMPQFGLSSTNSHWGYG-
ApXP001947715   197 VHQGPPHQFRGLGLSQRPFMEN-ASFSNHLRELDTYRRVKTEESPVLEQSYRSTNNQDEH
BGIBMGA008906   150 -------------------YGYGH-----PGPFSPFLLN-PGWLDAAYLNYAWADYFRPP
PHUM003687*     142 ----------------------------------------------------NAHWGYS-
Dmrun           238 -------------------YGYPH-----PGAFNPFMLN-PAWLDAAYMTYGYADYFR-H
ApXP001950158*  328 ------------------DVIHGYGGMFPMHGPLPPYGMIMEYKRLLEGAALPGSLNYQY
GB19482         236 FFASPLVDPLQPLPNPLQPLPNPLQPRDPLSSFRHAMPANCQNMSQFG-LTASNSWGYG-
BGIBMGA008907*  175 --------------GAWVADGGIGRPSETVPAAARRSVGDLNLGVTTKLRPRKLSRQPVY
BGIBMGA008905*  189 KLQRFDTGGIETNEITGELARKGAETSSAGLEPSTSTINIHWHPNSIGGSPLNADGSEET
GB16431         317 --ARVPALPRVPGLTRGTFPDAPSPFTPYLRDSEPYRRNKHHVGNNVTANSVSGACTNPN
Aphmm248704*    167 --------------------PFPFGTPLDPNRIAELPLKLSGLAHSWG-----TSFSRAL
CG34145         243 --------------------PFHFST-DPLSGFRMPPIGNCQ-------SASNTHWGYGS
GB11654         146 -------------------YQYGHG----FPGLG--LLN--PWVDVAYLGHAW-------

BGIBMGA008970*  233 --------------------------GASAYPAYLP------------------------
ApXP001948981*  233 --------------------------HSTAYSTWPGG-----------------------
Dmlz            464 -------------------------AVAAAAAAVAVTPT---------------------
Tc06542         191 ------------------------------------------------------------
PHUM008646      191 ------------------------------------------------------------
Tc06536         268 -------------------------HLAGLPGPPP-------------------------
Tc05796*        211 ------------------------------------------------------------
PHUM003686*     214 -------------------------YTSPTGYNYSNDYN---------------------
PHUM003688*     189 -------------------------TLKDVWRPYMHP-----------------------
SpRUNT1         251 -------------------------HQGQASKPWSYYNP---------------------
GB15836         272 LPRGSLPPHCLPPPPGHHSHTHPPAAGASAFNPFHHSIE---------------------
CG42267         318 -------------------------GLRGLSGDSQQHQQQ--------------------
Tc05566*        243 --------------------------AAGAYSPYFTP-----------------------
ApXP001947715   256 -------------------------SDIPVGVPESSNWC---------------------
BGIBMGA008906   185 ------------------------------------------------------------
PHUM003687*     149 --------------------------AATPYTSYLGS-----------------------
Dmrun           272 ------------------------------------------------------------
ApXP001950158*  370 ------------------------------------------------------------
GB19482         294 --------------------------STAGYAGYLP------------------------
BGIBMGA008907*  221 -------------------------YHPAYFCPKAVMRF---------------------
BGIBMGA008905*  249 -------------------------LLLGLYNRFYYASS---------------------
GB16431         375 -------------------------NTDPTASPASSAHL---------------------
Aphmm248704*    202 --------------------------PSTAYPPYLANNCGPPTFQHSNFTNIFPYNGTTT
CG34145         275 --------------------------AASAYSPYLAS-----------------------
GB11654         172 ------------------------------------------------------------
```

```
BGIBMGA008970*  243 -----------------SCAAPAASQFNPPALGFAGTVPDQTATQDFTANN-----TVLPD
ApXP001948981*  244 -------------GGCTNFTPP------ALATGFTGSVAASELHTSLSAGHQHDLFTTCTV
Dmlz            478 ------GGGGGVAAGGVAGGAGAGLVQQLSSNYSSPNSTINSDCQVYKPNAPHIQAAEMM
Tc06542         191 ---------TNVPTQTNLH---------ASLIKGATQLPPTNGEFYLPPQPQFHP-----
PHUM008646      191 ---------TNTELCKLPVPPCSTLSKGSSVPFPPDIHLGPSMATFSHSTN-PTP-----
Tc06536         278 -----------EWAMLGGRHPYPPGPFVPHHHPHFPPHMFAALDRPINTSP---------
Tc05796*        211 ------GPSSQSSDGSQSS--------YKQDSQDG-------------SLHPNPPPCPPA
PHUM003686*     228 ------SLESSATS-SIGHLS-----SVLPEHTPGSSMQQTGGDYVYGSMKPESE-----
PHUM003688*     201 ---------------GTDVDGSSPADYHVLTAEALAERTLDGLLAEHPGELV---------
SpRUNT1         265 ------YQSSVAQLSDTSILSAQIKTEPTELALLGQQNSTLQQYPKPDSLYPTSITRSSE
GB15836         311 -----QRSPRLPGPNSEPSRNDLGPISVSVREVTPTTSPGNPGPGLLTTATVAAPTPPAE
CG42267         333 -------QQQHQLATVGAAHSTTSPEGSPTTTTTSGTQLSAFVQPPMTSSPPPVTSLQHD
Tc05566*        254 -------------STLGSCAAPTASQFNTPALGFSGSTPDQTSTQDAFGSTS-NVTSLLPD
ApXP001947715   270 ------GYSSSSYSPSAAPLGGAAY-AAYPDTTGPAIMAMDAPSAMYGSGHPEVQQNNHM
BGIBMGA008906   185 ---------QMREPSTLIKGGAAPLTTPPVTIPGADLFPFPPAVTNLPPGGLIPP-----
PHUM003687*     160 -------------GALSSCGT---GSFN-PSLGFATSGEQTPVGHEGFGSS----------
Dmrun           272 ---------QAAAQAAQVHHPALAKSSASSVSPNPNPSVATSSSSAVQPSEYPHPAAAVA
ApXP001950158*  370 -------------IAANYFQLHNAMEASQMYCDLPPQSAFNFAGYNGLPGLPPTPPHTEEE
GB19482         304 -------------GPLSSCAAQ--ASFPPPPPPPPPPPPSSSSLASFAGAASMNTPTAPDS
BGIBMGA008907*  235 ------GLKGGAAVVTILET------LELTSQVLPELPLGHADYSGFQSSTTSYKGSPSG
BGIBMGA008905*  263 -----------LKVGGGIHGLRLPLAYQVDLEPVQRKNNMNSVALYFGSSS---------
GB16431         389 ------GPNSGSS-AHQDC--------YKHSPQHGDTGTAGSAEWTYPSAAPSYPAPPVS
Aphmm248704*    236 EVIQQQTINAGTDGNNSSTRTATEPLGPISVNVSVDSATTRSHRTGIRGHRGLLTTVSGS
CG34145         286 -------------SGLSSCTTPTSAQFNNPALGFTCSSNDQSNNQDFGGATNRDCVPMLPD
GB11654         172 ----------HLPHPAFVKG--------TIPMPSTDLFPPTFPPSVLPSYPFDHVK----

BGIBMGA008970*  282 TTGVDLDQQLSGLVGS-------------------SPSHHG-------------------
ApXP001948981*  286 NAQTTAAERVAYYCFCFLYKVMP------PFADTTVPTVNGPG-----------------
Dmlz            532 GAGEWTNGSSSSAAAYYHSHAHHPHAHHAHAHAHLQHQMALPPPPPPPPAAAPVSVGVGGNGA
Tc06542         228 -----PHNFLP-PNGILP-------QLSLSDSP---------------------------
PHUM008646      236 -----TSYLGPHFLGYSTS----MEKLLLPQLPKLN------------------------
Tc06536         318 ----RIANEPSSTSLGP------ISINCTPAHSTTSPKTS--------------------
Tc05796*        244 -----TWPDYSYTP---YSPQTGYYDHQDSATSLHLPTVLPEP-----------------
PHUM003686*     271 ---ILPCG----------------------------NTLVQTG-----------------
PHUM003688*     238 ----RTGSPHLVCTMLPP------HWR----SNKTLPVAFK-------------------
SpRUNT1         319 VQDPRFVYPSTPAAVSSVSFTPSSMSVLSSGVESPRTILPMTP-----------------
GB15836         366 PTTTTTTPSPSSGHHSHFQGLHLLGATGSIFGSIFAPLLPQSSWLYN-------------
CG42267         386 NNNNNSNNNNSSSHIDAGFESDSISVTGSPRKSLGSPLTHDEEEAEA-------------
Tc05566*        301 TSTTDLDQHLG-LVSSQN-----------HTNHSQSSTHTSS------------------
ApXP001947715   323 ---TDFCGTHQQHQHHQHQQQQQQQQHEHQQQHQQEILKPAL-----------------
BGIBMGA008906   231 -----PGAFLP-PNGLLPFPPHPA-ELALKSLPPELSLKSGL------------------
PHUM003687*     194 --TANVMDHLN-------------------------------------------------
Dmrun           323 AAAGQPAAMMPSPPGAAPATPYAIPQFPFNHVAAAAAAKAATPHAFHP------------
ApXP001950158*  418 ------------------------------------------------------------
GB19482         350 TAGSTVTSGTGAGSTAQQDAFS------AVSSLVPDSTTTGQS-----------------
BGIBMGA008907*  283 -------------------------TSSSLTELNPTTPVTTQR-----------------
BGIBMGA008905*  303 ----WAKDRGYICDLGHSSLVGWSTWRRMGSASPALPPTNS-------------------
GB16431         434 -----SGGSFSPIPGGAFSYPGEALSHHPTTEPVPLPTVLPSD-----------------
Aphmm248704*    296 GAHKRHHHHHHHHHKRHRDNRSEKSAVAAVAADRLSPTVVAAS-----------------
CG34145         334 STASDLDQHLSSLVGSTSGQMTHHSLLGAGGQTSISSTVNGASGGGSA-----GAGTAGG
GB11654         210 ----YPAEYAT--------------TLPPKSSS---------------------------
```

```
BGIBMGA008970*  304 ----------------TLLPRYN-NNADY----SLSTGPRSLSDNSSQPESPVQDDLLTS
ApXP001948981*  323 ----------------DLEHQLLLSSATSNCHQQHDRTGFPTVRGYHQQQSSVNDQSMPP
Dmlz            592 TMGMGMGVGVGMGMNHYGGGYDSANSLEAGQYAAHLPAVLPEMHGHGFATDPYQTAGYGG
Tc06542         248 -------------------LLRPSVGPMDQLS--LRISPVSNSQS--------------P
PHUM008646      263 --------------ETILSGTNGNSTARVISSSSSSSSPESNNNTTGN-----QQRIPSP
Tc06536         348 -------------------PTQVGLLTTAGAPLSPPEDDISVTAS--------PTPSPQP
Tc05796*        279 --------------GHNEFINT------------SLTSPPPMLNSAKSELDPMRYSDNST
PHUM003686*     283 ---------------NNSNISSG-------GTVIYSSGSCQSSYHTGWSTNNYQSYPNY-
PHUM003688*     265 -------------------VVALGEVLDGTLVTVRAGNDENFCGELRNCTAVMKNQVAKF
SpRUNT1         362 --------------NPFPLSSQDIFSSSSTATPVTLTSPPYLPNSPPYPLYPHLYMSSPS
GB15836         413 --PLYHTQQYVLEPEWHALALRMAQQRLQRPDQARVEETRKVRAGSSSPESALKEAKTRR
CG42267         433 ------EAEAEAEAEAEAEEAEVGGLSRNGGGIQGPLHSESSPGSGGAFTALIQRSGKNP
Tc05566*        331 ----------------LLVPRYSTNHSDF----GLS-GPRSLSDNSSAAESPVQDDILST
ApXP001947715   363 ---------------HHQDLETGV------GGGYYPSSWPTAEHHHHHHPSSYHHHHHHP
BGIBMGA008906   266 ------------TPYEALRQFQNNVSSMDTSS--ARLSPTSSRQSGSPRSMANASPDRSK
PHUM003687*     203 --------------------------------------QRQNSDDQTRLGSGVQR-----
Dmrun           371 ------------YNFAAAAGLRARNAALHHQSEPVHVSPASSRPSSSS---PTQQHVLLK
ApXP001950158*  418 ----------------PTAGTPAAQDDCGVPSKRQRLAAESARAVATATAAAAATATTTG
GB19482         387 ---------------DPLDPLSSLMSGTPSQRYQDYVSPRSLSTDSSTTESPVHEE----
BGIBMGA008907*  301 ----------------------------------YDSNYYNSWPANSYNYNNYQYNNINN
BGIBMGA008905*  340 -------------------ASTSALHSTRAASSAYTAPRTPTASRKRVLKFLIFTDVTSD
GB16431         472 --------------TSQQDTYTPNCVSMYPSHGASSTSSLPLVPAKSSDPDIFAGGGTGS
Aphmm248704*    339 -----------AAAAINNNDNNNNNGSGSGAGEEHDKNKRRTADDDHRVRRGARTTPSPP
CG34145         389 GAGSGGGAGGGAGGNSILVPRYHTNASNEYNVHSSQNGPRSLSD-SSQAESPVQEDLLTT
GB11654         225 ----------------------SSSAQATIPTSPSRTPPKSPSESGSE------------

BGIBMGA008970*  343 NTTNIGHNHSN--------------------------------------TSNFP----SL
ApXP001948981*  367 PAPDLHPTISSG-----------------------------------------P----RS
Dmlz            652 GNTGGGSASKSELD-------------------------------------YGGSYNQAW
Tc06542         273 TAQETKIHSTVDQ-----------------------------------------------
PHUM008646      304 EENATESNNTSTG-----------------------------------------------
Tc06536         381 PPAFPGAPPPP----------------------------------------------IQN
Tc05796*        313 -----YYPNNWTP-----------------------------------------------
PHUM003686*     320 ------YNS---------------------------------------------------
PHUM003688*     306 NDLRFVGRSGRAP------------------------------------------EPKRI
SpRUNT1         408 SQTYYDSSHLPMLP-------------------------------------SSTRPEDKQ
GB15836         471 DDEDNRDPARVRS------------------------------------GPDSPDGSIEV
CG42267         487 TELFGGFAAAGGN------------------------------------------HFAPS
Tc05566*        370 QST-LGVNHVN--------------------------------------NTNFP----LH
ApXP001947715   402 AAVAYQYNAAVVPP-------------------------------------PPPPPP---
BGIBMGA008906   312 TDSKSEANSIHDA-----------------------------------------------
PHUM003687*     220 ------------------------------------------------------------
Dmrun           416 LNTSIETSSIHEQ-----------------------------------------------
ApXP001950158*  462 PDDRLRPEPCKRP-----------------------------------------------
GB19482         428 --QGFGQNYGN----------------------------------------YFP------
BGIBMGA008907*  327 NPACIQ------------------------------------------------------
BGIBMGA008905*  381 NPISMRAHSTTSP------------------------------------------ANSRP
GB16431         518 GSPGYHYGPSWTSG-------------------------------------PATPVPVAS
Aphmm248704*    388 PPPTLRPPPPAQP---------------------------VAQLPPHIMAAAAATVSPRS
CG34145         448 NTPNLGSTAGGGAANGGAGSNAGSGAAGSGAGGAGGASSAVGNPAMLGANQNFPGIVNQA
GB11654         251 -SAPEEVRSAFVP-----------------------------------------------
```

```
BGIBMGA008970*   361 MGSQNSSYG----------------SSNCNNSLYPVLPASLLYSQLYTAANQTHNFHPLH
ApXP001948981*   382 TDSSTPDSP----------------TGAGGSTDDMMAIGHAHGYDQLGGGGGYHGGGHPT
Dmlz             675 SNGYQNYQYG------SCLATAQYGPQAAPPPQPPPPPPVVLCPQLYSTVNQNQIHLHLH
Tc06542          286 ----------------------------STSE-NSDDEDIDVVKSAFVPIKPANLILQEV
PHUM008646       317 ----------------------------EISDNSDNEETIDVVKSAFRQVKSNSRSHPYS
Tc06536          395 NQLFNNALA----------------ASLFLNAPLLPPPGQWFYSQFYP--HDWAWMN---
Tc05796*         321 -NSYPNNYN-------------YYN---TPNNNQQYPSTMVLYPPLYSTQN--QIHFHLH
PHUM003686*      323 ----PSGAT-------------------SQTPYLNPTPSVVLYPHLYSTVNQNQIHLHLH
PHUM003688*      324 LESGDGKLS----------------SKRTSSPIGNISP----------------------
SpRUNT1          431 EIKHDNRPG-------------------EGIPHIPHPEMSLTVALNYGSHPQSQIELNTA
GB15836          495 DDRNEQESG----------------KDRARSDESVGGQDSPRISPVRSDTEDTTADATAF
CG42267          505 GHSFNPALA----------------AQLFLQSPLLPQSSQWLYTQLYGSYSDLPWLRNAA
Tc05566*         387 QNMTSSSYP----------------SSNCNNSIYPVLPASLLYSQLYSAANQSHNFHSLH
ApXP001947715    422 -PHHPVQMT-------------------TEPPPVPPPTTMVLYPHLYSTVNQNQIHLHLH
BGIBMGA008906    325 ----------------------------TITD-ESDEEPIEVVKSAFHPTRPANLELQEM
PHUM003687*      220 --------------------------------------------TVHSAADSSG------
Dmrun            429 ----------------------------SASDGDSDDEQIDVVKSEFDLDKSLDVAPLRM
ApXP001950158*   475 ----------------------------KRESAPSPDIDIVSVGVVRSSSPEVQLKPRQE
GB19482          440 ---------------------------------TPGVLPSILYSQLYGNQFQNSE-----
BGIBMGA008907*   333 -----------------------------SHAPYINPNPQMILPNLYSTVNQNQIHVHLH
BGIBMGA008905*   399 CSPQDDDIS----------------VTASMSPPPDERPGAFTRPRKPLQPLPAPSSLFHS
GB16431          541 HNYNPNYQG-------------YYN---NPSQNYISPAPMVLYPQLYSTVNQNQIHLHLH
Aphmm248704*     421 QHPSASAPP--PPVTQPVPLVAPSLFSLFLNAPLLQPHTQWLYSQLYPNPYLSHIRNTMI
CG34145          508 QHSAASAYGGGTGVVGGGHGSAAAAAAGCNGSLYPVLPASLLYSQLYTAANQSAHGFHSH
GB11654          263 ----------------------------IRLNTLPPTTSVVTATSASSPERLPTKKGVVV

BGIBMGA008970*   405 SNSIHSTQN--HHNELQTMMDQISSTTNNHRQHGNQ--------DLLLGGSSSCAAAAAR
ApXP001948981*   427 TG-----MQLPASLQLYSQLYHASAAGNMTRHHHHHH-------HYHQPGDDEGGGGGVG
Dmlz             729 SS-----------------------EKLEQYLGTATSADHLTIGSLTGSSRSSIEIGQDQ
Tc06542          317 Q------------------------------PDSTVQDKELKKNELKAPSS----RSIKS
PHUM008646       349 S----------------------------SGKDSGIIRTCRKSSPIKSVSENKKKQETVV
Tc06536          434 -LRHHSLLP--RSSSPLELGHNPNGSTKSEGVDDEVKSDDGKAKEEKKGKDEGVNLSLHR
Tc05796*         362 GSS----------------------EKIDQYLSTES-------LSLTPTRAIELAQTVTT
PHUM003686*      360 G---------------------------DKAAESLQCSGEELAATIVANSN-----NLTI
PHUM003688*      346 --------------------------------------KKHTSGPESPKKKSPSLSPGKR
SpRUNT1          472 RTMG-------------------------QSMGGMHNSGVALMQQHRNHSPVTNISPMQQ
GB15836          539 QEDGTAHLRPSMENSNLDQEVLATDGHQVSRRESKIRLEVGTVDLSTKNKGQDKENVGQD
CG42267          549 AAAAANINPGQENSGIPPLGSNPDHDGVNLIKRCVTLITHNPPDAENANPNASPPVSSTR
Tc05566*         431 S---HTTQS--HHNDLQTVMDQLS-TSNQRQMNGST--------DLLLSNNGTCAAAAAR
ApXP001947715    462 APSSAAEHQLQQLHINNNNNNNNNSIKDPSAAEHHQYVVDDVAALAAVAAAGNPAAQLTI
BGIBMGA008906    356 KRV--------------------------QAADSTVSDRPRTRNELKSTSQ----RTTRV
PHUM003687*      230 ------------------------------------------------------------
Dmrun            461 R---------------------------------CDLKAPSAMKPLYHESG----PGAVA
ApXP001950158*   507 K-----------------------------------------------------------
GB19482          462 ------------SPEQRAVADSCS------------------------------------
BGIBMGA008907*   364 SSS-------------------------DKYNLEQCIPSEIKISDIDGGISITTELQGTG
BGIBMGA008905*   443 ALAAQLFLNSPLLPTPPAWLYSQLYGGYDWWLRPPAPQEDPSSSPDREEESSTPSVAGKK
GB16431          585 G-----------------------------DLSEEQ-------VTIAGS-----NLTISS
Aphmm248704*     479 FDNESTAAAVAAAAAQKAVGGGIGGVVDDADGSAAEN-------ESPKKSPADEGEADGG
CG34145          568 TLPAHASPNSSVHGELQSVMDHISNVGVRQQHNIMAGGGVTHPGDLTLIGN--CGASVRN
GB11654          295 E---------------------------------------GSRNELKAPTA----LISRS
```

```
BGIBMGA008970*  455 GEDG----RVN-NLGQRGNPQPDSN------------------------------TVWRP
ApXP001948981*  475 VDDDHLQVSGASQRPTQQAVAATDHG-----------------------------AVWRP
Dmlz            766 YHQQVHHAQQQQQQQQQQQQVHHPQQQQVESAGEVGGSGAGGVESAREEDVGDLSQVWRP
Tc06542         343 LAEEKSPNTKIHHQQEITATK----------------------------------SVWRP
PHUM008646      381 ISDCSTITTTTTTQTSTQPQK----------------------------------TVWRP
Tc06536         491 CRKAAITLVRQKEEVSSSEKEHLKCNRLG--------------------------DVWRP
Tc05796*        393 EVAQSHETLEDTER------------------------------------THDPASVWRP
PHUM003686*     388 SS-----GTRSSIEIGIV----------HNNQGGLMSEDDRFTHNDRQG----DPSVWRP
PHUM003688*     368 ------LLNTIKQHKTNTGETPSKT----------------------------LKDVWRP
SpRUNT1         507 SMGQMNINTTVGHLPTVD--------------------------------DSRKEDVWRP
GB15836         599 LTTKRKEEDTEREAVVRGRRERSPKPR----------------------------QVWRP
CG42267         609 RSPSPVETIDLDDVSTTSRSASGSSGHGGGVGGG------GAVGPIRTRTPKPSADVWRP
Tc05566*        477 QDDA----RLTSNGAQRGPGQNSDA------------------------------VVWRP
ApXP001947715   522 SAAGSGGGNARGIEIGLLPPPPAVSLDEHHQAGDIISIGGRYSDRQQQQQQHTDLAVWRP
BGIBMGA008906   386 LS--TSPTSTKIANGTIPSHK----------------------------------SVWRP
PHUM003687*     230 --------------------------------------------------------VWRP
Dmrun           484 NSRQPSPETTTKIKSAAVQQK----------------------------------TVWRP
ApXP001950158*  508 --------------------------------------------------------IWRP
GB19482         474 --------------VRQEEVRPDNN-------------------------------VWRP
BGIBMGA008907*  399 EPSGIVQTCEANDEVKHG------------------------------MYGAGSQEVWRP
BGIBMGA008905*  503 RPASPEWSDQSVRTRSKSLTTPEKR----------------------------PVDVWRP
GB16431         604 NRLEIGVMGEENEQ------------------------------------RND---VWRP
Aphmm248704*    532 VGADDEVDRDGADASPAISPKTTAKQT----------------------------DVWRP
CG34145         626 IEDGNSNRQVAALAAHRGHHNPTDNGG----------------------------SVWRP
GB11654         312 MSPKRSPSPTKIS--SPPPAK----------------------------------PVWRP

BGIBMGA008970*  480 Y-----
ApXP001948981*  506 Y-----
Dmlz            826 Y-----
Tc06542         369 Y-----
PHUM008646      407 Y-----
Tc06536         525 Y-----
Tc05796*        417 Y-----
PHUM003686*     429 Y-----
PHUM003688*     394 Y-----
SpRUNT1         535 Y-----
GB15836         631 Y-----
CG42267         663 Y-----
Tc05566*        503 Y-----
ApXP001947715   582 Y-----
BGIBMGA008906   410 Y-----
PHUM003687*     234 Y-----
Dmrun           510 Y-----
ApXP001950158*  512 YGATDE
GB19482         489 Y-----
BGIBMGA008907*  429 Y-----
BGIBMGA008905*  535 Y-----
GB16431         625 Y-----
Aphmm248704*    564 Y-----
CG34145         658 Y-----
GB11654         336 Y-----
```
