# Supplementary material for: Evolutionary origin and genomic organisation of runt-domain containing genes in arthropods
Source: BMC Genomics. 2008 Nov 25;9:558. doi: 10.1186/1471-2164-9-558 (PMC2631020; doi:10.1186/1471-2164-9-558)
Supplement: Additional file 4 — Multiple sequence alignment of the RD protein sequences from a number of metazoan species. ClustalX alignment of RD protein sequences from a number of metazoan species including representative species from the insects, crustaceans, chelicerates, cnidarians, lophotrochozoans, echinoderms and chordates. [file 1471-2164-9-558-S4.pdf]

Additional file A4: multiple sequence alignment of metazoan RD domain sequences

```
MMU3                1 HAGELVRTDSPNFLCSVLPSHWRCNKTLPVAFKVVALGDVPDGTVVTVMAGNDENYSAEL
TakifugufrRUNX3     1 HAGELVRTDSPNFLCSVLPSHWRCNKTLPVAFKVVALGDVPDGTLVTVMAGNDENYSAEL
CG42267             1 HPGELVKTGSPHVVCTTLPTHWRSNKTLPIAFKVLALGEVMDGTIVTIRAGNDENFCGEL
PHUM003688          1 HPGELVRTGSPHLVCTMLPPHWRSNKTLPVAFKVVALGEVLDGTLVTVRAGNDENFCGEL
GB16431             1 HPGELVRTGSPYFLCSQLPTHWRSNKTLPVAFKVVALGEVVDGTLVTVRAGNDENCCAEL
MMU2                1 HPAELVRTDSPNFLCSVLPSHWRCNKTLPVAFKVVALGEVPDGTVVTVMAGNDENYSAEL
GGA2                1 HPAELVRTDSPNFLCSVLPSHWRCNKTLPVAFKVVALGEVPDGTVVTVMAGNDENYSAEL
TakifuguCbfa1       1 HPAELVRTDSPNFLCSVLPSHWRCNKTLPVAFKVVALGDIPDGTVVTVMAGNDENYSAEL
BfRUNT              1 HPGELVRTDSPNFVCSVLPSHWRCNKTLPVPFKVVALGDIPDGTLVTVMAGNDENYSAEL
NvRUNT              1 YPGELVKTDSPNFVCSVLPSHWRCNKTLPVAFKVVSLGDIPDGVIVSIAAGNDENFAAEL
Trichoplax          1 YPGELVRTDSPNFVCSVLPSHWRCNKSLPVPFKVVALGYMPDGVVVSLAAGNDENCSAEL
Dmlz                1 HPGELVRTSNPYFLCSALPAHWRSNKTLPMAFKVVALAEVGDGTYVTIRAGNDENCCAEL
PHUM003686          1 HPGELVRTGSPYFLCSVLPTHWRSNKTLPIAFKVVALGDVMDGTIVTVRAGNDENCCAEL
CiRUNT              1 HQGELIKTDSPNFLCTPLPQHWRVNKSLQTPFKVVALSDIPDGTTVTVMAGNDENYSAEL
TakifugufrRunt      1 IPRGLVQTDSPNFLCTSLPQHWRCNKTLPRPFTVFALGDVPDGVVVTVMAGNEENSSAEL
Spu07852            1 YPGELVKTESPNFICSLLPPHWRSNKSLPVAFKVVSLGETKDGTVVTIGAGNDENCCAEL
GB19482             1 HPGELVRTGSPHLVCTVLPAHWRSNKTLPVAFKVVALGEVGDGTLVTVRAGNDENCCAEL
Pacifastacus        1 HPGELVRTGSPNFVCTILPPHWRSNKTLPVAFKVIALGEVGDGTLVTVRAGNDENFCAEL
GB15836             1 HPGELVRTGSPHVVCTVLPAHWRSNKTLPVAFKVVALGEVGDGTLVTVRAGNDENCCAEL
PHUM003687          1 HPGELVRTGSPHFVCTVLPPHWRSNKTLPVAFKVVALGDVIDGTLVTVRAGNDENYCAEL
CG34145             1 HPGELIRTSSPLFVCTVLPPHWRSNKTLPVAFKVVSLGDIMDGTMVTVRAGNDENYCAEL
Dp290556            1 HPGELVATGSPCVLVSPLPKHWRSNKTLPNTFRVVVLGGAEDGTLVTVRAGNDENWSPEL
Dp290557            1 HPGELVRTGSPCVLCSSLPTHWRSNKTLPAAFRVVCLGSIDDGTLVTVRAGNDENWSAEL
Dp290555            1 LGGELVRTGSPCVICTALPTHWRSNKTLPTAFRVVCLGGVEDGTLVTVRAGNDENCSSEL
Dmrun               1 YHGELAQTGSPSILCSALPNHWRSNKSLPGAFKVIALDDVPDGTLVSIKCGNDENYCGEL
PHUM008646          1 YHGELVQTGSPAILCSALPNHWRSNKSLPIAFKVVALDDVVDGTLVTIRCGNDENFCGEL
GB11654             1 CHGDLVRTGSPAILCSALPSHWRSNKSLPVAFKVVALDDVSDGTLVTIRAGNDENCCGEL
capitella           1 HPGELVRTGSPNFVCSVLPSHWRSNKTLPVSFKVVALGEVKDGTKVTLNVGNDENCCGEL
lottia              1 HPGELVRTGSPNFVCSVLPSHWRSNKTLPVAFKVVSLGDVKDGTKVIIRAGNDENFCGEI
Cupiennius1         1 LPSELVKTGSPCFVCSVLPGHWRSNKTLPLPFKVICLGEVADGTMVTIRAGNDENFCGEL
SpRUNT1             1 YPGELVKTESPNFACSVLPNHWRCNKSLPVAFKVVSLGETKDGTMVTIAAGNDENYCAEL
Heliocidaris        1 YPGELVKTESPNFACSVLPSHWRCNKSLPVAFKVVSLGETKDGTMVTIAAGNDENYCAEL
MMU1                1 HPGELVRTDSPNFLCSVLPTHWRCNKTLPIAFKVVALGDVPDGTLVTVMAGNDENYSAEL
GGAB2               1 HPGELVRTDSPNFLCSVLPTHWRCNKTLPIAFKVVALGDVPDGTLVTVMAGNDENYSAEL
TakifugufrRunx1     1 HPGELVKTDSPNFLCSVLPTHWRCNKTLPIAFKVVALGDIPDGTLVTVMAGNDENYSAEL
Dp290554            1 HQGELIQTGSPYFLCTALPTHWRSNKSLPVAFRVVALGEIADGTVVVIRAGNDENYCCEL
IsDS935894          1 HPGELMRTGSPNLVCSVLPSHWRSNKTLPVAFKVVTLGDVCDGTLVTLRAGNDENYCAEL
IsDS722357          1 HPGELMRTGSPNVVCSVLPTHWRSNKTLPMSFRVLALGDVCDGTLVTLRAGNDENYCGEL
Tetranychus         1 YPGEFVRTGSPNLVCSALPTHWRSNKTLPVAFKVVALGQVNDGTMVTIRAGNDENFCPEL
```

```
MMU3               61 RNASAVMKNQVARFNDLRFVGRSGRGKSFTLTITVFTNPTQVATYHRAIKVTVDGPREPR
TakifugufrRUNX3    61 RNASAVMKNQVARFNDLRFVGRSGRGKSFTLTITVFTGPPQVATYHRAIKVTVDGPREPR
CG42267            61 RNCTAVMKNQVAKFNDLRFVGRSGRGKSFTLTIVISTNPIQIATYTKAIKVTVDGPREPR
PHUM003688         61 RNCTAVMKNQVAKFNDLRFVGRSGRGKSFTLTIIINSSPPQVATYAKAIKVTVDGPREPR
GB16431            61 RNSTTLMKNQVAKFNDLRFVGRSGRGKSFSITITVSTTPPQVATYTRAIKVTVDGPREPR
MMU2               61 RNASAVMKNQVARFNDLRFVGRSGRGKSFTLTITVFTNPPQVATYHRAIKVTVDGPREPR
GGA2               61 RNASAVMKNQVARFNDLRFVGRSGRGKSFTLTITVLTNPPQVATYHRAIKVTVDGPREPR
TakifuguCbfa1      61 RNASGVMKNQVARFNDLRFVGRSGRGKSFTLTITVFTNPPQVATYHRAIKVTVDGPREPR
BfRUNT             61 RNNQAVMKNQVARFNDLRFVGRSGRGKSFTLTITVFTSPPQVATYHRAIKVTVDGPREPR
NvRUNT             61 RNATAVMKNQVARFNDLRFVGRSGRGKTFSLTITVKTEPPQVATYCRAIKVTVDGPREPR
Trichoplax         61 RNSTAVMKNQVARFNDLRFIGRSGRGKSFTLTITVGTNPPQVATYNKAIKITVDGPREPR
Dmlz               61 RNFTTQMKNDVAKFNDLRFVGRSGRGKSFTLTITVATSPPQVATYAKAIKVTVDGPREPR
PHUM003686         61 RNCTAVMKNQIAKFNDLRFVGRSGRGKSFTITITISSSPPQVTTYTKAIKVTVDGPREPR
CiRUNT             61 RNASATMKGCVARFNDLRFLGRSGRGKSFNLTITIFSSPPQVATYQRAIKITVDGPREPR
TakifugufrRunt     61 RNATATMKQGFAHFNDLRFIGRSGRGKSFTVSINVLMSPPQIATLQKAIKVTVDGQRQPR
Spu07852           61 KNNIAVMKNQVARFNDLRFVGKSGRGKSFTLSIFVYTNPPQIATCNRAIKVTVDGPR-PR
GB19482            61 RNSTALMKNQVAKFNDLRFVGRSGRGKSFTLTITVSTTPPQVATYTKAIKVTVDGPREPR
Pacifastacus       61 RNNLALMKNQIAKFNDLRFVGRSGRGESFNLTITISTSPPQVTTYCKAIKVTVDGPREPR
GB15836            61 RNSTAVMKNQVAKFNDLRFVGRSGRGKSFTLTIMLQTSPPQVATLSKAIKVTVDGPREPR
PHUM003687         61 RNGTAVMKNQVAKFNDLRFVGRSGRGKSFTLTITISSSPPQVATYTKAIKVTVDGPREPR
CG34145            61 RNCTAVMKNQVAKFNDLRFVGRSGRGKSFTLTITVSTNPPHIATYNKAIKVTVDGPREPR
Dp290556           61 RNSVAQVKNNVAKFNDLRFIGRSGRGKSFTLTITICTSPPVVTTYNKAIKISVDGPREPR
Dp290557           61 RNGTAVMKNHVAKFNDLRFIGRSGRGKSFTLTITVNSSPPQVATYTKAIKVTVDGPREPR
Dp290555           61 RNATAIVKNHVAKFNDLRFIGRSGRGKSFTLTITVSTTPPQIATYSKAIKVTVDGPREPR
Dmrun              61 RNCTTTMKNQVAKFNDLRFVGRSGRGKSFTLTITIATYPVQIASYSKAIKVTVDGPREPR
PHUM008646         61 RNCTAVMKNQVAKFNDLRFVGRSGRGKSFSLTILISSTPFQIATYAKAIKVTVDGPREPR
GB11654            61 RNCTAVMKNQVAKFNDLRFVGRSGRGKSFSLTIQISTVPFQVATYTKAIKVTVDGPREPR
capitella          61 RNAVTYMKNHVAKFNDLRFVGRSGRGKSFNLTICVQTNPPQVATFQKAIKVTVDGPREPR
lottia             61 RNYTAYMKNRVAKFNDLRFVGRSGRGKTFTLTITVSSNPPQIASYNKAIKVTVDGPREPR
Cupiennius1        61 RNASAVMKNQVAKFNDLRFVGRSGRGKSFSLTISISTSPPHVVTYNEAIKVTVDGPREPR
SpRUNT1            61 KNNTAVMKNQVARFNDLRFVGRSGRGKSFTLSIFIYTNPPQIATYNRAIKVTVDGPREPR
Heliocidaris       61 KNNTAVMKNQVARFNDLRFVGRSGRGKSFTLSIFIYTNPPQIATYNRAIKVTVDGPREPR
MMU1               61 RNATAAMKNQVARFNDLRFVGRSGRGKSFTLTITVFTNPPQVATYHRAIKITVDGPREPR
GGAB2              61 RNATAAMKNQVARFNDLRFVGRSGRGKSFTLTITVFTNPPQVATYHRAIKITVDGPREPR
TakifugufrRunx1    61 RNATAAIKNQVARFNDLRFVGRSGRGKSFTLTITVFTSPPQVATYQRAIKITVDGPREPR
Dp290554           61 RNHTAVMKNQVAKFSDLRFVGRSGRGKSFTLSIIVSSSPVQVTTYNKAIKVTVDGPREPR
IsDS935894         61 RNASAVVKNQVAKFNDLRFVGRSGRGKSFTITITLSTNPPQVATYTKAIKVTVDGPREPR
IsDS722357         61 RNASAVMKNQVAKFNDLRFVGRSGRGKSFTITITLSTNPPQVATYSKAIKVTVDGPREPR
Tetranychus        61 RNASAIMKNQVAKFNDLRFVGRSGRGKSFTLTITLSTNPPQVATYCKAIKVTVDGPREPR
```

MMU3	121	RHRQK
TakifugufrRUNX3	121	RHRVK
CG42267	121	SKVRH
PHUM003688	121	SKTRH
GB16431	121	SKTRQ
MMU2	121	RHRQK
GGA2	121	RHRQK
TakifuguCbfa1	121	RHRQK
BfRUNT	121	RHRQK
NvRUNT	121	RHRTR
Trichoplax	121	SKSIM
Dmlz	121	SKTSP
PHUM003686	121	SKTRK
CiRUNT	121	RHRQK
TakifugufrRunt	121	RQRQK
Spu07852	120	RPKSK
GB19482	121	SKTRQ
Pacifastacus	121	SKAHA
GB15836	121	SKTRH
PHUM003687	121	SKTIK
CG34145	121	SKTRQ
Dp290556	121	SKSRQ
Dp290557	121	SKTRS
Dp290555	121	SKTQD
Dmrun	121	SKQSY
PHUM008646	121	TKTSF
GB11654	121	SKSNY
capitella	121	SKTSK
lottia	121	CKTSK
Cupiennius1	121	RQQQQ
SpRUNT1	121	RPKPK
Heliocidaris	121	RPKPK
MMU1	121	RHRQK
GGAB2	121	NTRQV
TakifugufrRunx1	121	RHRQK
Dp290554	121	TKSPG
IsDS935894	121	SKTHP
IsDS722357	121	NLTQA
Tetranychus	121	SKTTM
