# Supplementary material for: Evolutionary origin and genomic organisation of runt-domain containing genes in arthropods
Source: BMC Genomics. 2008 Nov 25;9:558. doi: 10.1186/1471-2164-9-558 (PMC2631020; doi:10.1186/1471-2164-9-558)
Supplement: Additional file 5 — Multiple sequence alignment of arthropod RD domain sequences. ClustalX alignment of the RD from a number of arthropod species, including three insect species and four non-insect arthropods. [file 1471-2164-9-558-S5.pdf]

Additional File A5: multiple sequence alignment of arthropod RD domain sequences

```
CG34145        1  ERTLGDFLTEHPGELIRTSSPLFVCTVLPPHWRSNKTLPVAFKVVSLGDIMDGTMVTVRA
PHUM003687*    1  ERTLGDFLSEHPGELVRTGSPHFVCTVLPPHWRSNKTLPVAFKVVALGDVIDGTLVTVRA
GB19482        1  ERTLGDFVSEHPGELVRTGSPHLVCTVLPAHWRSNKTLPVAFKVVALGEVGDGTLVTVRA
GB15836        1  ERTLDGLFAEHPGELVRTGSPHVVCTVLPAHWRSNKTLPVAFKVVALGEVGDGTLVTVRA
PHUM003688*    1  ERTLDGLLAEHPGELVRTGSPHLVCTMLPPHWRSNKTLPVAFKVVALGEVLDGTLVTVRA
CG42267        1  ERTVDVLLAEHPGELVKTGSPHVVCTTLPTHWRSNKTLPIAFKVLALGEVMDGTIVTIRA
IsDS935894     1  ERALSQVLAEHPGELMRTGSPNLVCSVLPSHWRSNKTLPVAFKVVTLGDVCDGTLVTLRA
IsDS722357     1  ERALTQVLAEHPGELMRTGSPNVVCSVLPTHWRSNKTLPMSFRVLALGDVCDGTLVTLRA
Tetranychus    1  ERLLNEFIDEYPGEFVRTGSPNLVCSALPTHWRSNKTLPVAFKVVALGQVNDGTMVTIRA
Cupiennius1    1  ERLFTDVIDNLPSELVKTGSPCFVCSVLPGHWRSNKTLPLPFKVICLGEVADGTMVTIRA
GB11654        1  -NAMHETLQACHGDLVRTGSPAILCSALPSHWRSNKSLPVAFKVVALDDVSDGTLVTIRA
Dp290554       1  -SSAFDTMVDHQGELIQTGSPYFLCTALPTHWRSNKSLPVAFRVVALGEIADGTVVVIRA
Dmrun          1  FASLHEMLQEYHGELAQTGSPSILCSALPNHWRSNKSLPGAFKVIALDDVPDGTLVSIKC
PHUM008646     1  FSNVHEILQEYHGELVQTGSPAILCSALPNHWRSNKSLPIAFKVVALDDVVDGTLVTIRC
Dp290555       1  DRALTEIIGDAPGELVRTGSPCVICTALPTHWRSNKSLPTAFRVVCLGGVEDGTLVTVRA
Dmlz           1  ERLVQKRQQEHPGELVRTSNPYFLCSALPAHWRSNKTLPMAFKVVALAEVGDGTYVTIRA
GB16431        1  ERLVGEAQAEHPGELVRTGSPYFLCSQLPTHWRSNKTLPVAFKVVALGEVVDGTLVTVRA
PHUM003686*    1  ERILHEIQAEHPGELVRTGSPYFLCSVLPTHWRSNKTLPIAFKVVALGDVMDGTIVTVRA

CG34145       61  GNDENYCAELRNCTAVMKNQVAKFNDLRFVGRSGRGKSFTLTITVSTNPPHIATYNKAIK
PHUM003687*   61  GNDENYCAELRNGTAVMKNQVAKFNDLRFVGRSGRGKSFTLTITISSSPPQVATYTKAIK
GB19482       61  GNDENCCAELRNSTAIMKNQVAKFNDLRFVGRSGRGKSFTLTITVSTTPPQVATYTKAIK
GB15836       61  GNDENCCAELRNSTAVMKNQVAKFNDLRFVGRSGRGKSFTLTIMLQTSPPQVATLSKAIK
PHUM003688*   61  GNDENFCGELRNCTAVMKNQVAKFNDLRFVGRSGRGKSFTLTIIINSSPPQVATYAKAIK
CG42267       61  GNDENFCGELRNCTAVMKNQVAKFNDLRFVGRSGRGKSFTLTIVISTNPIQIATYTKAIK
IsDS935894    61  GNDENYCAELRNASAVVKNQVAKFNDLRFVGRSGRGKSFTITITLSTNPPQVATYTKAIK
IsDS722357    61  GNDENYCGELRNASAVMKNQVAKFNDLRFVGRSGRGKSFTITITLSTNPPQVATYSKAIK
Tetranychus   61  GNDENFCPELRNASAIMKNQVAKFNDLRFVGRSGRGKSFTLTITLSTNPPQVATYCKAIK
Cupiennius1   61  GNDENFCGELRNASAVMKNQVAKFNDLRFVGRSGRGKSFSLTISISTSPPHVVTYNEAIK
GB11654       60  GNDENCCGELRNCTAVMKNQVAKFNDLRFVGRSGRGKSFSLTIQISTVPFQVATYTKAIK
Dp290554      60  GNDENYCCELRNHTAVMKNQVAKFSDLRFVGRSGRGKSFTLSIIVSSSPVQVTTYNKAIK
Dmrun         61  GNDENYCGELRNCTTTMKNQVAKFNDLRFVGRSGRGKSFTLTITIATYPVQIASYSKAIK
PHUM008646    61  GNDENFCGELRNCTAVMKNQVAKFNDLRFVGRSGRGKSFSLTILISSTPFQIATYAKAIK
Dp290555      61  GNDENCSSELRNATAIVKNHVAKFNDLRFIGRSGRGKSFTLTITVSTTPPQIATYSKAIK
Dmlz          61  GNDENCCAELRNFTTQMKNDVAKFNDLRFVGRSGRGKSFTLTITVATSPPQVATYAKAIK
GB16431       61  GNDENCCAELRNSTTLMKNQVAKFNDLRFVGRSGRGKSFSITITVSTTPPQVATYTRAIK
PHUM003686*   61  GNDENCCAELRNCTAVMKNQIAKFNDLRFVGRSGRGKSFTITITISSSPPQVTTYTKAIK

CG34145      121  VTVDGPREPRSKT
PHUM003687*  121  VTVDGPREPRSKT
GB19482      121  VTVDGPREPRSKT
GB15836      121  VTVDGPREPRSKT
PHUM003688*  121  VTVDGPREPRSKT
CG42267      121  VTVDGPREPRSKV
IsDS935894   121  VTVDGPREPRSKT
IsDS722357   121  VTVDGPREPRNLT
Tetranychus  121  VTVDGPREPRSKT
Cupiennius1  121  VTVDGPREPRRQQ
GB11654      120  VTVDGPREPRSKS
Dp290554     120  VTVDGPREPRTKS
Dmrun        121  VTVDGPREPRSKQ
PHUM008646   121  VTVDGPREPRTKT
Dp290555     121  VTVDGPREPRSKT
Dmlz         121  VTVDGPREPRSKT
GB16431      121  VTVDGPREPRSKT
PHUM003686*  121  VTVDGPREPRSKT
```
